# Kinetic Dissection of the Reaction of Human GDP-l-Fucose Synthase

**DOI:** 10.1021/acscatal.5c02722

**Published:** 2025-07-29

**Authors:** Denis Smyshliaev, Martin Pfeiffer, Udo Oppermann, Bernd Nidetzky

**Affiliations:** † Institute of Biotechnology and Biochemical Engineering, Graz University of Technology, NAWI Graz, Petersgasse 12, A-8010 Graz, Austria; ‡ Austrian Centre of Industrial Biotechnology (acib), Krenngasse 37, A-8010 Graz, Austria; § Botnar Research Centre, Nuffield Department of Orthopaedics, Rheumatology and Musculoskeletal Sciences, National Institute of Health Research Oxford Biomedical Research Unit (BRU), 6396 University of Oxford, OX3 7LD Oxford, U.K.; ∥ Oxford Centre for Translational Myeloma Research, University of Oxford, OX3 7LD Oxford, U.K.

**Keywords:** GDP-l-fucose, fucosylation, GDP-l-fucose synthase (GFS), GDP-4″-keto-6″-deoxy-d-mannose epimerase/reductase, enzyme mechanism, multistep kinetic pathway, kinetic isotope effects

## Abstract

GDP-l-fucose is a universal sugar donor for the cellular biosynthesis of l-fucose-containing glycans. Its supply comes primarily from the reaction of GDP-l-fucose synthase (GFS), also known as GDP-4″-keto-6″-deoxy-d-mannose epimerase/reductase. GFS converts GDP-4″-keto-6″-deoxy-d-mannose by epimerization at both C-3″ and C-5″ followed by NADPH-dependent reduction of the carbonyl at C-4″. Here, we report kinetic and structural characterization of human GFS with the aim of dissecting the multistep pathway of the enzymatic reaction. Kinetic isotope effects due to [3″-^2^H] or [5″-^2^H] in GDP-4″-keto-6″-deoxy-d-mannose and [4*S*-^2^H] in NADPH were masked in the steady-state rate of the wild-type enzyme, indicating that the immediate catalytic steps were not rate-limiting for the overall reaction. An isotope effect, however, appeared with GFS variants defective in catalysis of an elementary step, when the isotope probe for that particular step was used in the reaction (C116S: C-3″ epimerization, [3″-^2^H]-substrate; Y143F: 4″-keto group reduction, [4*S*-^2^H]-NADPH). Evidence from steady-state and transient kinetic studies combined with reaction simulations revealed that GFS uses a random mechanism of substrate binding and product release, with the peculiarity that at saturating conditions of substrate and NADPH, the product dissociation happens from abortive GFS complexes with NADPH/GDP-l-fucose and NADP^+^/GDP-4″-keto-6″-deoxy-d-mannose and involves GDP-l-fucose release as the rate-determining step. GFS complex structures with NADP^+^ and NADP^+^/GDP suggest an induced-fit conformational change required to unbind the GDP moiety for dissociation from the enzyme as the molecular cause of the slow product release. Collectively, these results establish the basic kinetic framework of the GFS reaction, which is critical for understanding this important enzyme mechanistically and in its role as an inhibitor target to control glycan fucosylation *in vivo*.

## Graphical abstract

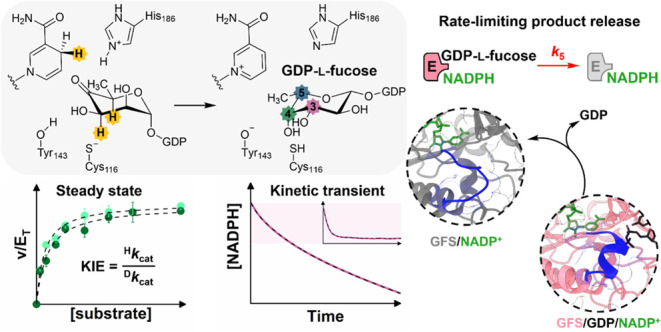

## Introduction

l-Fucose (**1**; Figure S1) is the most common 6-deoxy-hexose in nature.^1^ It is a key constituent of many biologically important glycans in diverse organisms from all domains of life.^1–3^ Within the human body, l-fucose is critical for the proper function of major glycan-mediated processes in different settings of the physiology.^3–5^ l-Fucose in protein- and lipid-linked glycans on cell surfaces is generally important for cell–cell interactions and plays key roles in signaling.^3,6^ l-Fucose is comprised in the glycans of the ABO blood group antigens^7–9^ as well as in glycans important for maintaining the gut microbiome.^10,11^ l-Fucose-containing glycans (e.g., the tetra-saccharide sialyl-Lewis^X^ present on the surface of leukocytes)^4,12^ are causative for initiating the inflammatory response and are critically involved in ontogenesis, cellular differentiation, and immune system development.^3^

Aberrant or defective expression of fucosylated glycans is connected with the onset and progression of major diseases, including cancer^13^ and chronic inflammatory conditions such as rheumatoid arthritis and pancreatitis.^4^ Concepts of fucose-targeted therapies are pursued with the aim of inhibiting^14–16^ or activating^17,18^ the crucial cellular functions of fucosylated glycans.

Another area where control of fucosylation has received strong attention is recombinant production of therapeutic glycoproteins, monoclonal antibodies in particular.^19^ Avoiding the so-called “core fucosylation” of protein *N*-glycans represents a promising strategy to enhance antibody potency for therapeutic uses.^20–22^ Approaches to manipulate the levels of l-fucose-containing glycans *in vivo* are often based on small-molecule inhibitors (e.g., structural analogues of l-fucose that are metabolically “activated” to the corresponding GDP derivative)^23–29^ that target the immediate protein machinery of cellular fucosylation. This machinery comprises a set of specific glycosyltransferases that attach l-fucose residues to nascent glycans.^3,30^ It additionally comprises enzymes for the supply of guanosine 5′-diphosphate (GDP)-l-fucose (**1a**; Figure 1a), which is the universal sugar donor for cellular fucosylation.^1,3,6^

**Figure 1:**
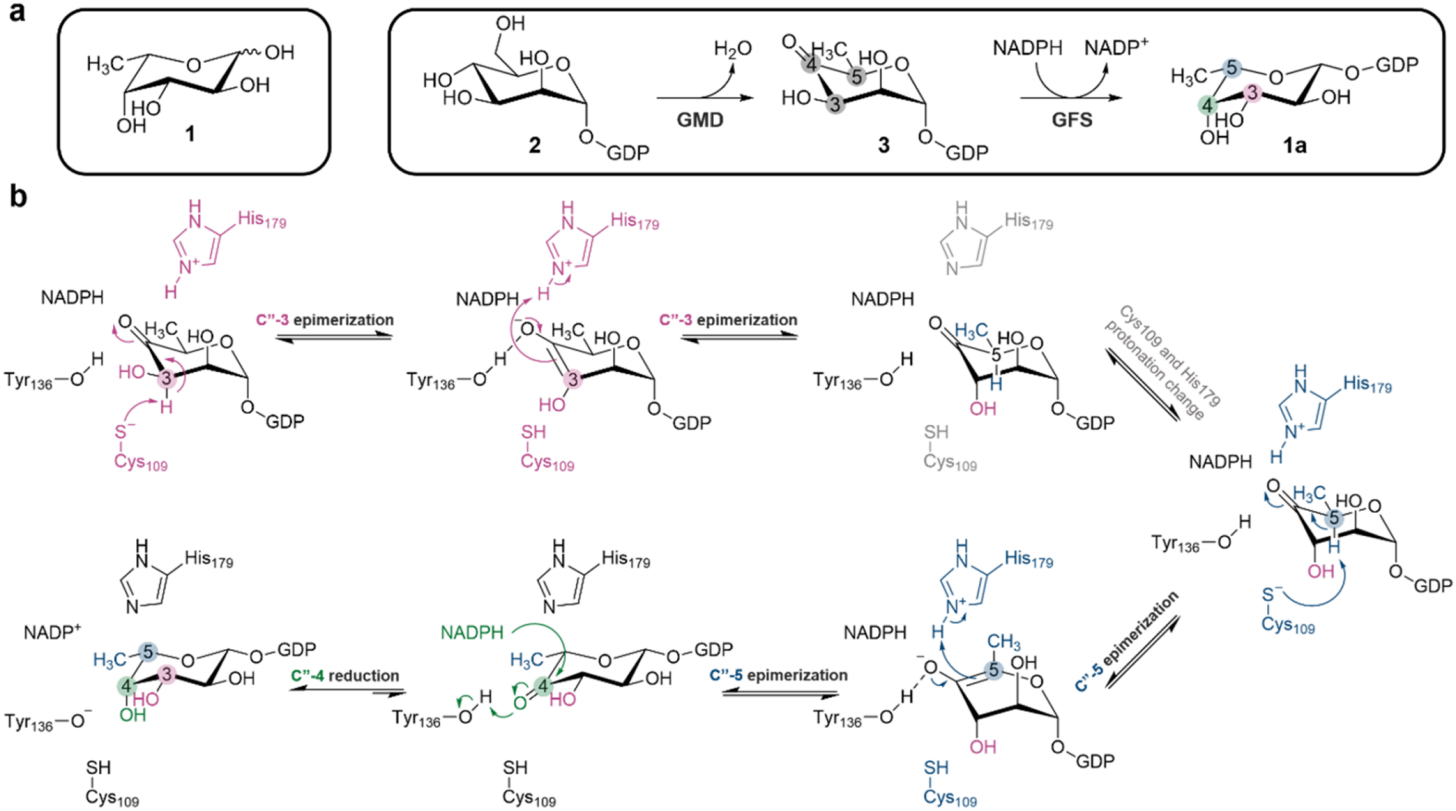
l-Fucose and its *de novo* biosynthesis involving GFS. (a) Biosynthetic pathway from GDP-d-mannose (**2**) by GMD and GFS. (b) Proposed mechanism of GFS based on studies of the enzyme from .^31^

In mammalian cells, GDP-l-fucose is derived primarily (≥90%) through biosynthesis from GDP-d-mannose (**2**).^6^ The pathway involves two enzymes (Figure 1a) and proceeds *via* GDP-6″-deoxy-4″-keto-d-mannose (GDP-6″-deoxy-α-d-*lyxo*-hexos-4″-ulose, **3**).^3^ GDP-l-fucose synthase (GFS), also known as GDP-4″-keto-6″-deoxy-d-mannose epimerase/reductase, converts the intermediate **3** into GDP-l-fucose.^3^

The GFS reaction is an astounding transformation to be performed by a single enzyme active site. It involves epimerization at both the C-3″ and C-5″ of substrate **3** followed by an NADPH-dependent reduction of the carbonyl at C-4″.^32,33^ GFS succeeds in the precise coordination of the steps of epimerization (C-3″ before C-5″), preceding the reduction step so that only a single isomeric product is released.^33–37^

Mechanistic studies of GFS have been performed with the enzyme from .^31^ It was shown that each epimerization proceeds under general base/general acid-catalytic assistance from a conserved cysteine/histidine dyad of residues (Figure 1b).^38^ The carbonyl reduction happens by pro-*S* stereospecific hydrogen transfer from NADPH,^34^ most probably under general acid-catalytic assistance from a conserved tyrosine (Figure 1b). It was further shown for GFS that NADPH is not required for the two epimerization reactions,^34^ yet the formation of a ternary GFS complex with NADPH and substrate **3** seems to precede the chemical steps of the enzymatic process. Product **1a** and NADP^+^ are released to complete the GFS catalytic cycle. A random kinetic mechanism was proposed for GFS based on product inhibition and fluorescence binding studies.^34^ The kinetic isotope effect (KIE) due to deuteration of substrate **3** (3″-^2^H or 5″-^2^H) was completely masked in the enzymatic rate,^31^ and the KIE due to [4*S*-^2^H]-NADPH was small (1.4),^34^ suggesting that there are physical steps in the enzymatic mechanism of GFS, which are considerably slower than the chemical steps. The rate-limiting step of the reaction of GFS was not identified. The kinetic mechanism of any other GFS, including the human form of the enzyme, has not been elucidated.

Detailed kinetic characterization of the human GFS reaction is however critical to understand this important enzyme mechanistically^31,32,38^ and as an inhibitor target.^24,26–29^ GFS inhibition by GDP-activated analogues of l-fucose was shown to be effective in modulating glycan fucosylation *via* depletion of the cellular GDP-l-fucose.^26,29^ The precise mode of GFS interaction with these inhibitors and the efficacy of the inhibitors to tune down the enzyme activity^24^ depend on the distribution of GFS forms under the reaction conditions at steady state, which in turn is determined by the kinetic mechanism.

Here, we present the results of kinetic and structural investigations of the human GFS.^39^ We use steady-state and transient kinetic analyses in combination with kinetic isotope effects (KIE) due to the specific deuteration of substrate **3** ([3″-^2^H] or [5″-^2^H]) or NADPH ([4*S*-^2^H]) to probe the individual chemical steps catalyzed by the enzyme. The wild-type GFS and enzyme variants featuring the site-directed replacement of one of the putative catalytic residues were studied. GFS complex structures with NADP^+^ and NADP^+^/GDP are reported. Collectively, evidence is presented that provides a detailed mechanistic-kinetic description of the multistep pathway of the GFS reaction. The results provide the essential kinetic framework for analyzing the inhibition of the human enzyme, showing that the major steady-state form of the enzyme is the abortive complex with GDP-l-fucose and NADPH. The findings suggest that GDP-activated analogues of l-fucose primarily compete with substrate **3** for binding to the GFS complex with NADPH. Mechanistically, the results support the suggestion for the role, and the interplay, of the active-site residues in catalysis to the three chemical steps of the GFS reaction. The human GFS is structurally similar to its homologue in^35,36^ and the central features of the active site are conserved in both enzymes (Figure S2). Results of the current study may have general relevance for a better understanding of the GFS class of enzymes.

## Materials and Methods

All materials and methods used are described in the Supporting Information. Determination and reporting of steady-state enzyme kinetic data followed the Standards for Reporting Enzymology Data (STRENDA; https://www.beilstein-institut.de/en/projects/strenda/) and kinetic parameters for the wild-type form of human GFS are deposited in the database under DOI: 10.22011/strenda_db.IGXI1L.

## Results

### Wild-Type and Variant Forms of GFS and Their Steady-State Kinetic Characterization

Putative catalytic residues of the human GFS (Tyr143, Cys116, and His186; Figure S2) were replaced individually by a residue incompetent to fulfill the proposed function of the original side chain (Y143F, C116A, H186A; see Figure 1b). They were additionally replaced by a residue that could be functionally conservative (C116S, H186K) in the enzymatic mechanism of epimerization. The lysine arguably can substitute the histidine as a general acid. The serine is certainly not a good general base, but earlier work on the GFS^31^ suggests its capacity to weakly restore the function of the original Cys109.

Purified human GFS enzymes (Figure S3) were assayed for conversion of substrate **3** in the presence of NADPH. The enzymes as-isolated contained bound NADPH in an occupancy that varied among the different enzymes between 0.24 and 0.90 (Table S1 and Figure S4). NADP^+^ was found in low amount (≤6 mol % of GFS subunit). The result is relevant later when discussing the kinetic mechanism of the wild-type GFS. The specific activity of C116A and H186A was below the detection limit of the activity assay used, which is ∼0.01% of the specific activity of the wild-type enzyme (0.44 ± 0.02 U/mg; *n* = 5). Y143F retained ∼3% wild-type specific activity and released a single reaction product, identified as GDP-l-fucose.

Retention of activity in Y143F may be explained tentatively by a water molecule binding in place, partially substituting the function of the phenolic hydroxy group of the original tyrosine. Precedence for the proposed mechanistic scenario of Y143F is provided by UDP-glucuronic acid 4-epimerase,^40^ which is related to GFS by common membership to the extended families of short-chain dehydrogenases/reductases.^33,41^ Biochemical^42^ and crystallographic^43^ studies of the relevant Y149F variant of the epimerase support the notion of activity retained (∼0.1%) due to a water molecule occupying a similar active-site position as the phenolic hydroxy group in the wild-type enzyme.

The C116S variant showed substantially lowered specific activity (∼4.4 × 10^2^-fold) and altered product specificity compared to the native GFS. It converted substrate **3** into GDP-l-fucose (∼30%) and another product (∼70%) that was identified in a separate study from this laboratory as GDP-d-altrose (**4**). The C116S-catalyzed path from substrate **3** to GDP-d-altrose (**4**) involves C-3″ epimerization, followed by reduction of the C-4″ carbonyl before the second epimerization at C-5″ could take place. The result is consistent with evidence for the corresponding C109S variant of GFS,^31^ reported by Lau and Tanner. H186K retained substantial activity only 4-fold lower than that of wild-type GFS. H186K released GDP-l-fucose as a single product of the conversion of substrate **3**.

Kinetic parameters of the three GFS variants are summarized in Table 1 along with parameters of the wild-type enzyme. Note that aqueous solutions of substrate **3** contain about ∼24% of the hydrated form (compound **3a**, featuring a geminal C-4″ diol instead of a C-4″ carbonyl; Figure S5). The substrate concentrations reported always included the portion of hydrate **3a**. Whenever substrate **3** is mentioned henceforth, it is done with the understanding that **3a** is also present. Apparent *K*_m_ for substrate **3** varied only little (≤4-fold) among the different enzymes, implying that the larger differences in the specific activity were entirely due to changes in the *k*_cat_. C116S showed the greatest defect in *k*_cat_ (∼10^3^-fold decrease) followed by Y143F (∼10^2^-fold decrease) and H186K (5.9-fold decrease).

**Table 1:** Summary of the Kinetic Characterization of GFS Enzymes^a^

		wild-type	H186K	C116S	Y143F
steady-state kinetic parameters	*k*_cat_ (s^–1^)	2.11 ± 0.03	0.36 ± 0.03	(2.9 ± 0.3) × 10^–3^	(2.5 ± 0.2) × 10^–2^
	*K*_m_^**3**^ (μM)	1.7 ± 0.2	0.8 ± 0.2	2.6 ± 0.7	3.3 ± 0.6
	*K*_m_^NADPH^ (μM)	0.75 ± 0.03	-^b^	-^b^	-^b^
kinetic isotope effects on *k*_cat_	[3″-^2^H]-**3**	0.98 ± 0.05	1.04 ± 0.03	1.6 ± 0.2	1.01 ± 0.07
	[5″-^2^H]-**3**	1.10 ± 0.04	0.99 ± 0.03	0.93 ± 0.07	1.1 ± 0.1
	[4*S*-^2^H]-NADPH	1.03 ± 0.05	1.12 ± 0.08	1.0 ± 0.2	3.9 ± 0.3
kinetic isotope effects on *k*_cat_/*K*_m_	[3″-^2^H]-**3**, ^D^ *k*_cat_/*K*_m_^**3**^	0.9 ± 0.2	1.0 ± 0.2	-^b^	1.2 ± 0.2
	[5″-^2^H]-**3**, ^D^ *k*_cat_/*K*_m_^**3**^	0.9 ± 0.1	1.2 ± 0.4	-^b^	0.9 ± 0.3
	[4*S*-^2^H]-NADPH, ^D^ *k*_cat_/*K*_m_^**3**^	1.4 ± 0.4	1.1 ± 0.5	-^b^	4 ± 1
	[4*S*-^2^H]-NADPH, ^D^ *k*_cat_/*K*_m_^NADPH^	1.6 ± 0.2	-^b^	-^b^	-^b^
rate constants from stopped-flow experiments	*k*_ss_ (s^–1^)^c^	2.17 ± 0.01	0.38 ± 0.01	(2.6 ± 0.2) × 10^–3^	(2.7 ± 0.3) × 10^–2^
	*k*_cat_^SF^ (s^–1^)	2.2 ± 0.1^d^	0.39 ± 0.04^e^	-^b^	-^b^

a The corresponding experimental data are shown in Figures S9–S10 and 2a–c.

b Not determined.

c *k*_ss_ was determined by linear fit of the steady-state phase of multiple-turnover stopped-flow progress curves.

d eqs S7, S8, and S10 were used to constrain the simulation fitting of the transient progress curves and to calculate the *k*_cat_^SF^ for the wild-type enzyme according to mechanism b.

e eq S13 was used to constrain the simulation fitting of the transient progress curve and to calculate the *k*_cat_^SF^ for H186K according to Scheme S2. For further details, see the Materials and Methods section of the Supporting Information.

### Kinetic Isotope Effects on Steady-State Kinetic Parameters

Substrate **3** deuterated at C-3″ or C-5″ and [4*S*-^2^H]-NADPH were synthesized as specific isotope probes of elementary chemical steps of the GFS reaction (Scheme S1). The degree of deuteration was shown to be ≥98% based on NMR data (Figures S6 and S7) and the NADP^+^ content of NADPH and [4*S*-^2^H]-NADPH was ≤1%, as indicated by HPLC (Figure S8). Initial rates were acquired at the steady state based on measurements of NADPH consumption by absorbance. A set of KIEs (^D^ *k*_cat_, ^D^ *k*_cat_/*K*_m_) was obtained for each enzyme (Figures S9,S10) and the results are summarized in Table 1. For wild-type GFS, the KIEs on *k*_cat_ were not different from unity within the limits of the error. The KIE on *k*_cat_/*K*_m_ (substrate) was also unity for both [3″-^2^H]- and [5″-^2^H]-substrate. When [4*S*-^2^H]-NADPH was used, the ^D^ *k*_cat_/*K*_m_ was slightly greater than unity (1.4–1.6) and the same for the substrate and coenzyme. For C116S, the ^D^ *k*_cat_ was elevated in the reaction with the [3″-^2^H]-substrate, whereas no KIE (^D^ *k*_cat_ = ∼1.0) was found in reactions with [5″-^2^H]-substrate or [4*S*-^2^H]-NADPH. H186K did not exhibit a KIE on *k*_cat_ and *k*_cat_/*K*_m_ (substrate) irrespective of the isotope probe used. Y143F showed no KIE when [3″-^2^H]- or [5″-^2^H]-substrate was used. A large KIE of ∼4 appeared, however, when [4*S*-^2^H]-NADPH was used. The KIE was the same on *k*_cat_ and *k*_cat_/*K*_m_ (substrate).

### Transient Kinetic Analysis

Rapid-mixing stopped-flow experiments were performed under “multiple-turnover” conditions where the substrate and coenzyme were present in molar excess (≥50-fold) over the enzyme used. Time courses from the reactions of wild-type GFS and H186K showed curves composed of an initial phase of rapid decrease in the NADPH absorbance (“transient burst”) followed by a slower steady-state phase in which the absorbance decreased linearly with time (Figure 2a,b).

**Figure 2:**
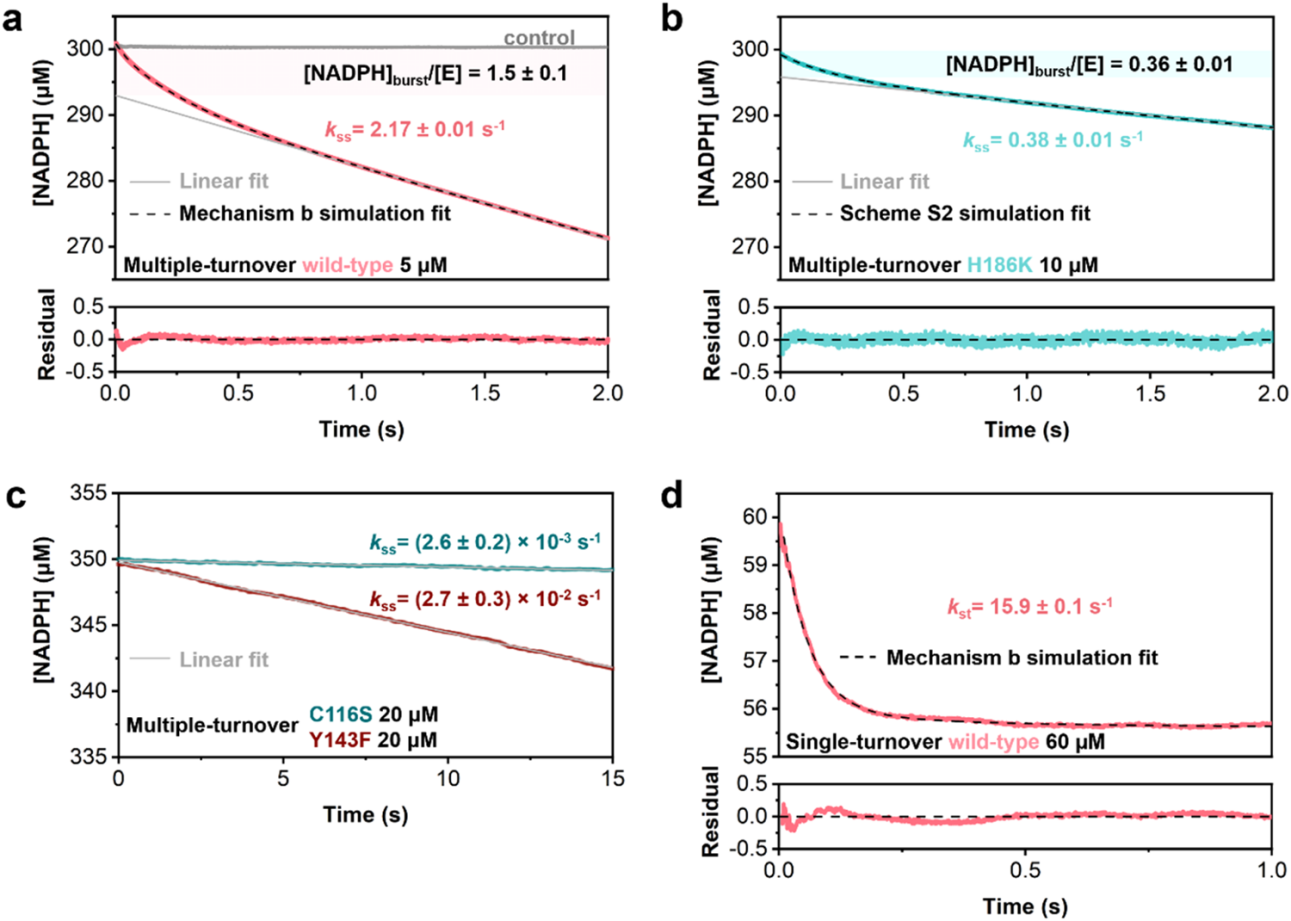
Transient kinetic analysis of the GFS reaction. Multiple-turnover progress curves for the wild-type enzyme (a), H186K (b), and C116S and Y143F (c). The enzyme solution was mixed with a solution of NADPH and substrate (reaction) or just NADPH (control, gray line in panel (a)). Conditions (concentrations after mixing): [GFS] (subunit) shown in the panels; [NADPH] = 300 μM (a, b) or 350 μM (c); [substrate 3] = 250 μM (a, b) or 300 μM (c). A Tris buffer (10 mM, pH 8.0) with 25 mM NaCl was used. Averaged data are shown from three experiments at 37 °C. (d) Single-turnover progress curves for the wild-type enzyme. Conditions: [GFS] (subunit) shown in the panel; [NADPH] = 60 μM; [substrate 3] = 4.0 μM; other conditions same as in panels (a–c). Data are shown in color, dashed lines are the global model fit (a, b, d), and gray lines are straight-line fit (eq S2) extrapolated back to zero time (a–c). The amplitude of NADPH consumed in the burst phase was calculated as [NADPH]_burst_/[E] where [E] is the molar concentration of the GFS subunit. For the global model fits, the distribution of residuals is shown additionally. For full details of the experimental methods and the fitting used, see the Materials and Methods section in the Supporting Information. The reported concentrations of substrate **3** include the ∼24% portion of the hydrated (geminal C-4″ diol) form.

Reactions of C116S and Y143F gave linear absorbance traces from which a burst phase was clearly absent (Figure 2c). The steady-state rate constants (*k*_ss_, s^–1^; eq S2) determined from the linear parts of the NADPH absorbance traces were in excellent agreement with the corresponding *k*_cat_ values from the initial-rate measurements (Table 1 and Figure S11).

Nonlinear fits of the progress curves with eq S3 yielded the NADPH consumed in the burst phase together with the associated transient rate constant (*k*_obs_, s^–1^). Common scenario of kinetic mechanism resulting in a presteady-state burst is that of a relatively fast chemical transformation that is followed by a slower physical step(s) postcatalysis.^44^ Neither of the reactions of wild-type GFS and H186K was consistent with a simple kinetic mechanism. In the wild-type reaction, the NADPH consumed in the burst (7.5 ± 0.1 μM; *n* = 3) exceeded the molar equivalent of enzyme used (5.0 μM) by 1.5-fold (Figure 2 and Table S2). In a linear sequence of microscopic steps, the burst equivalent (NADPH consumed/enzyme used) cannot be higher than unity.^44^ In the H186K reaction, the NADPH consumed in the burst (3.6 ± 0.1 μM; *n* = 3) was only ∼36% of the molar equivalent of enzyme (10 μM), yet the ratio of the rate constants *k*_obs_ and *k*_ss_ was even higher (∼11) than that for the wild-type reaction (∼2; Figure 2a,b; Table S2). The transient rate constant *k*_obs_ is composite of microscopic rate constants for the product release (*k*_P_), forward (*k*_1_), and reverse (*k*_–1_) directions of the chemical transformation (*k*_obs_ = *k*_1_ + *k*_–1_ + *k*_P_; Scheme S2; see ref 44). It cannot therefore be used directly to calculate the expected magnitude of the NADPH consumed in the burst ([NADPH]_burst_) relative to the enzyme used (E). However, the relationship [NADPH]_burst_/[E] = *k*_1_(*k*_1_ *+ k*_–1_)/(*k*_1_ *+ k*_–1_ *+ k*_P_)^2^ defines the portion of total enzyme turned over into product in the kinetic transient.^44^ These considerations imply that unless H186K embodied a significant change in the ratio of *k*_1_ and *k*_–1_ compared to wild-type GFS, the burst magnitude ([NADPH]_burst_/[E]) would be expected to be the same or similar for the two enzymatic reactions. The kinetic behavior of both enzymes, therefore, required explanation and was addressed in experiments described in the following sections.

To examine a possible contribution of the product release to rate limitation in the reaction of wild-type GFS, we performed stopped-flow experiments under “single-turnover” conditions (Figure 2d; *n* = 3) where the enzyme and NADPH (both 60 μM) were present in large excess over substrate **3** (4.0 μM). The observed decrease in NADPH absorbance was best-fitted by a single-exponential decay (eq S4) with an associated rate constant *k*_st_ of 15.9 ± 0.1 s^–1^. The *k*_st_ exceeded *k*_cat_ by ∼8-fold. Based on the NADPH used, the available substrate was converted fully in the reaction under these conditions. The observation of complete substrate consumption in a single kinetic phase is interesting considering that an aqueous solution of substrate **3** represents a mixture of free-carbonyl (∼76%) and hydrate (∼24%) forms (Figure S5). The hydrate cannot be a substrate for NADPH-dependent reduction, but it could arguably engage in the first epimerization at C-3″, where the deprotonation would initially generate a C-3″ carbanion-like species. In this configuration, the adjacent C-4″ remains a geminal diol, preventing the mechanistically relevant enediol formation; however, the resulting electronic destabilization might drive rapid dehydration at this stage to restore the carbonyl character at the C-4″.^45^ We have no way of determining whether the GFS is specific for binding only the reactive free-carbonyl form of the substrate. However, given that spontaneous dehydration of carbonyl hydrates in solution at neutral pH proceeds very slowly (<0.1 s^–1^),^46^ the observed rate coefficient *k*_cat_ supports the idea of an on-enzyme dehydration step, thus preventing that dehydration becomes limiting for substrate utilization.

The single-turnover *k*_st_ was higher considerably (∼4-fold) than the *k*_obs_ (= 3.8 s^–1^; Table S2) determined from the burst phase by single-exponential fit. We therefore examined fit by a double exponential (eq S5) and show that it gives improved description of the burst phase (Figure S11 and Table S2). The fast rate constant (*k*_obs1_ = 15.4 ± 0.1 s^–1^) was now consistent with *k*_st_ from the single-turnover reaction. The mechanistic origin of a transient burst composed of two kinetic phases was however not clear, requiring the evaluation of alternative kinetic models for the enzymatic reaction. We excluded at this stage models that expand the chemical step into multiple steps or describe the product release as two sequential steps. The reason is that all of these models lead to progress curves involving a lag phase, either before the burst when the catalytic chemistry is composite of multiple steps^47,48^ or before the steady state when the products are released sequentially.^49,50^ The multiple-turnover progress curves of GFS determined experimentally show a continuous transition from the burst to the steady-state phase, with a lag clearly lacking.

### Transient-State Kinetic Isotope Effects

To further probe the isotope sensitivity of the catalytic steps of the GFS reaction, we performed single-turnover experiments with the wild-type enzyme using deuterated substrate **3** or [4*S*-^2^H]-NADPH. The stopped-flow traces of NADPH consumption are shown in Figure 3a–c together with the associated single-exponential fits (eq S4). The transient-state KIE is the ratio of the rate constant *k*_st_ for the reaction with unlabeled and deuterium-labeled substrate/coenzyme. Deuteration at C-3″ resulted in a KIE of 1.4 ± 0.1, whereas deuteration at C-5″ gave a KIE of close to unity (1.03 ± 0.05). Use of [4*S*-^2^H]-NADPH yielded a KIE of 1.9 ± 0.1.

**Figure 3:**
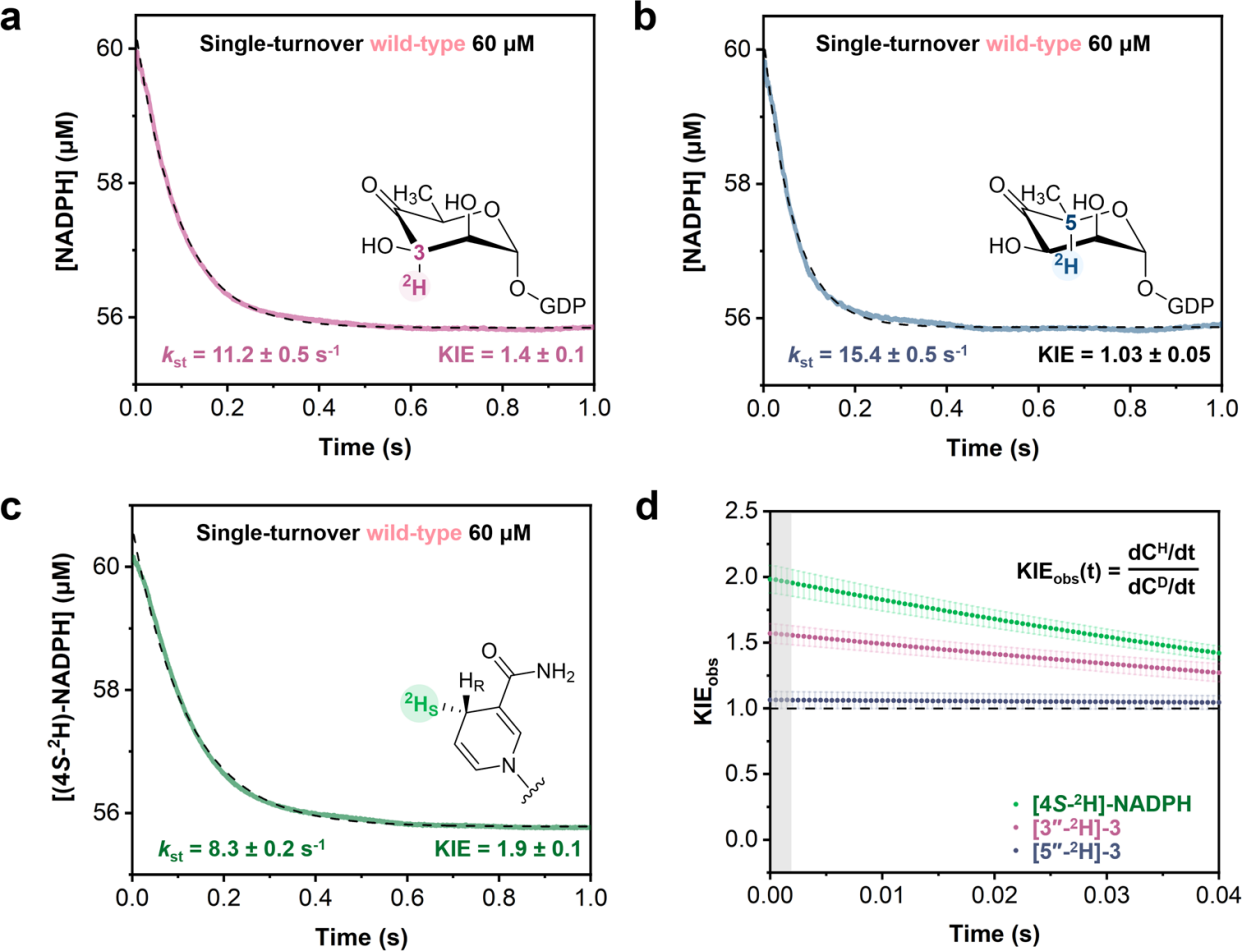
Analysis of transient-state KIEs in single-turnover stopped-flow experiments performed with wild-type GFS. Single-turnover progress curves with [3″-^2^H]-**3** (a), [5″-^2^H]-**3** (b), and [4*S*-^2^H]-NADPH (c). Conditions: [GFS] (subunit) shown in the panel; [coenzyme] = 60 μM; [substrate **3**] = 4 μM. A Tris buffer (10 mM, pH 8.0) with 25 mM NaCl was used. Averaged data are shown from *n* = 3 experiments at 37 °C. Data are shown in color, and dashed lines are exponential decay fits (eq S4). (d) Time-dependent kinetic isotope effects, defined as indicated in the figure. The ratio of derivatives was calculated from the lines of best fits to eq S4. Error bars show the standard deviation fit to triplicate experiments. Highlighted in gray is dead time of the stopped-flow instrument (∼2 ms). The reported concentrations of substrate **3** include the ∼24% portion of hydrated (geminal C-4″ diol) form.

Interpretation of transient-state KIEs is complicated by the fact that the observable isotope effect, expressed as the ratio of the time derivatives of the concentration for the unlabeled and deuterium-labeled substrate, that is, KIE_obs_ = (dC^H^/dt)/(dC^D^/dt), is time-dependent. Fisher and co-workers^51,52^ showed that in cases of enzymatic reactions where the recorded signal is derived from the molecular species after the isotope-sensitive step, the KIE_obs_ corresponds to the kinetically unmasked KIE at t = 0 and then decreases with time. Palfey and Fagan have expanded theory of KIEs on initial rates in transient kinetics,^53^ and da Silva and co-workers provide insightful discussion.^54,55^ Here, the observed KIEs for the hydride transfer step (reaction with [4*S*-^2^H]-NADPH) and C-3″ epimerization step were time-dependent, and extrapolation to t = 0 (Figure 3d) gave values of 2.0 and 1.6, respectively. The KIE_obs_ values at t = 0 were equivalent to those derived from direct exponential fitting, suggesting that the intrinsic isotope effect was not temporally resolved under the applied stopped-flow conditions. Extrapolation to t = 0 may not be warranted in this case, as discussed lucidly by Palfey and Fagan.^53^ Interestingly, the step of C-5″ epimerization involves no KIE.

### Equilibrium Binding of Ligands

Fluorescence titration studies were performed to analyze equilibrium binding of GFS ligands NADPH, NADP^+^, substrate **3**, and product **1a**. Quenching of intrinsic protein fluorescence was used as a reporter of ligand binding. The GFS crystal structures presented later reveal several tryptophan residues lining the binding pockets for coenzymes (Trp38, Trp87, Trp229, and Trp311) and the substrate (Trp208). The fluorescence properties of these residues can arguably be affected by ligand binding. To eliminate spectral interference of NADPH, we synthesized the analogue 1″,4″,5″,6″-tetrahydro-NADPH (NADPH_4_; Figures S1, S12, S13), which is unreactive with the enzyme in the reduction step, but mimics the native NADPH with respect to the structure and charge of the nicotinamide ring.^56^ Additional advantage of NADPH_4_ is that it allows for the formation of a stable ternary complex with enzyme and substrate **3**. Results are shown in Figures S14 and S15, and dissociation constants (*K*_d_) are summarized in Table S3. NADPH_4_ (*K*_d_ = 0.3 ± 0.1 μM) binds 27-fold more tightly to the free GFS than does NADP^+^. Substrate **3** and product **1a** both bind to GFS complexes with NADPH_4_ and NADP^+^. The binding affinity of both compounds is considerably higher for enzyme-NADPH_4_ than for enzyme-NADP^+^, whereas substrate **3** affinity for the free GFS is the lowest (Table S3).

### Simulations of Enzymatic Reaction Time Courses

Global fitting and simulation were used to analyze multiple-turnover progress curves from the reactions of wild-type GFS and H186K. A kinetic mechanism of random substrate binding and product release (Figure 4a,4b) was proposed by arguments as follows. Random binding of substrate **3** and NADPH was supported by normal KIEs of similar magnitude on the corresponding *k*_cat_/*K*_m_ when [4*S*-^2^H]-NADPH was used in the reaction (Table 1). In an ordered mechanism, the ^D^ *k*_cat_/*K*_m_ for the substrate that binds first is expected to be unity.^49^ This effect results because the KIE on the chemical step is suppressed completely by the infinite commitment to forward catalysis under the used conditions when the concentration of the second substrate is saturating.^49^ Fluorescence titration studies show that NADPH_4_ and substrate **3** both bind to the free wild-type enzyme, consistent with a random mechanism (Figure S14 and Table S3). An ordered mechanism of product release was ruled out because it would be inconsistent with the observed burst magnitude of greater than unity and the burst biphasic behavior.^44^

**Figure 4:**
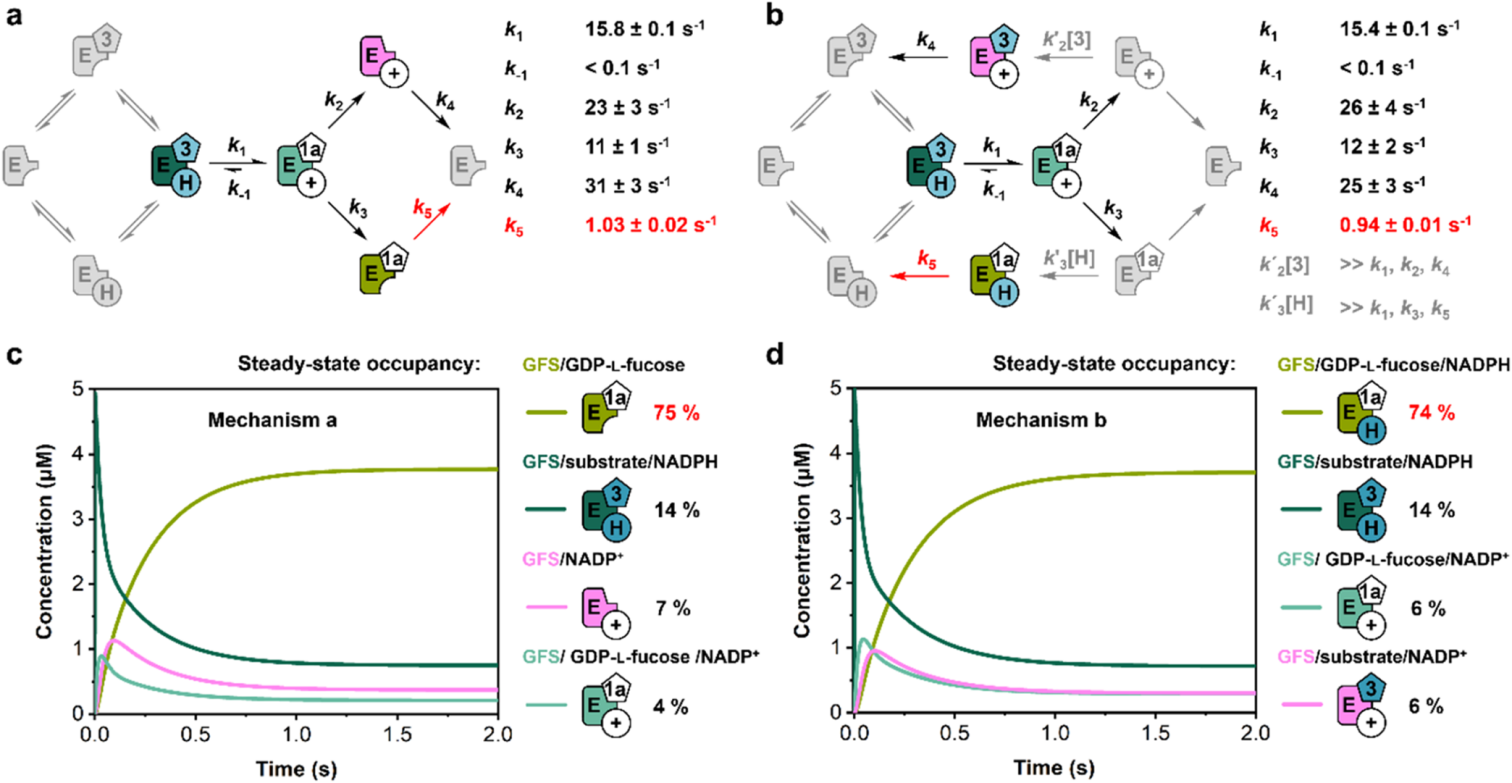
Proposed random kinetic mechanism of the GFS. (a) Mechanism a: Product dissociation recycles the free enzyme. (b) Mechanism b: Product release *via* abortive ternary complexes circumvents the formation of a free enzyme. For each mechanism, the microscopic rate constants from global simulation fitting to transient stopped-flow curves are shown. Substrate **3** and product **1a** are identified by their compound number, **H** and **+** show NADPH and NADP^+^, respectively, and E is the GFS. Enzyme forms shown in gray do not accumulate in significant amount at steady state. (c, d) Simulated progress curves for mechanisms a and b under the conditions of the multiple-turnover stopped-flow experiment (5 μM GFS, 300 μM NADPH, 250 μM **3**) and using the associated rate constants from panels (a) and (b), respectively. The major enzyme forms of the reaction are shown. Highlighted in red are the rate-limiting step (a, b) and prevalent enzyme species at steady state (c, d).

A random mechanism implies that the product release proceeds *via* binary GFS complexes with NADP^+^ or GDP-l-fucose from which the free enzyme could regenerate by dissociation (Figure 4a; mechanism a). Alternatively, the binary GFS complexes might bind substrate **3** or NADPH to form abortive ternary complexes (GFS/substrate/NADP^+^, GFS/GDP-l-fucose/NADPH) that could release the NADP^+^/product and so bypass the free form of enzyme while entering a new catalytic cycle of reaction (Figure 4b; mechanism b). Mechanism b seemed plausible considering that under conditions of NADPH and substrate **3** present at concentrations that are fully saturated at the steady state, the conversion of binary into abortive ternary complexes should be favored strongly. Moreover, there was additional evidence that GFS binds NADPH tightly: the enzyme as-isolated exhibited 46% occupancy by NADPH (Table S1). Fluorescence titration data (Table S3) furthermore support the idea that GFS prefers the reduced over the oxidized nicotinamide coenzyme for binding.

Global fit of both mechanisms (Figure 4a,4b) gave an excellent description of the multiple- and single-turnover progress curves (Figure 2a,2d), with an estimate for the rate constant of NADPH conversion (*k*_1_^a^ = 15.8 ± 0.1 s^–1^ and *k*_1_^b^ = 15.4 ± 0.1 s^–1^) that agreed very well with the transient rate constant determined directly from the single-turnover experiment. Note that during global fitting, the value of *k*_cat_ was constrained based on the steady-state turnover equation, incorporating all relevant rate constants derived from the proposed kinetic mechanism. The relationship of the microscopic rate constants of the mechanism and the observable kinetic constants from the experiments are shown in eqs S7–S10. The microscopic rate constants for the reactions *via* the free enzyme (Figure 4a; mechanism a) and *via* the abortive ternary complexes (Figure 4b; mechanism b) are summarized in Table S4 and Figure 4a,4b, respectively. Both mechanisms predict the *k*_cat_ values in excellent agreement with the results of initial-rate experiments and account fully for the burst magnitude exceeding unity. They localize the rate-limiting step at the release of GDP-l-fucose, yet differ in the enzyme complex from which this release takes place, as revealed by the simulation results shown in Figure 4c,4d.

It is not possible to distinguish between the two mechanisms by the kinetic data. However, based on the evidence in Figure 4c,4d, we reasoned that discrimination would be possible by the enzyme form that predominates at the steady state: enzyme/GDP-l-fucose for mechanism a and enzyme/GDP-l-fucose/NADPH for mechanism b. We therefore measured the content of NADPH and NADP^+^ in GFS during the reaction at the steady state (Figure S16). The experimental occupancy of the enzyme by NADPH (0.71) and NADP^+^ (0.19) was in good agreement with the expectation from the simulations for mechanism b (Figure 4b; NADPH: 0.88, NADP^+^: 0.12). Additional evidence was provided from equilibrium binding studies (Figure S15 and Table S3) that showed ∼12-fold tighter binding of GDP-l-fucose to enzyme-NADPH_4_ than to enzyme-NADP^+^. Mechanism a was ruled out by these findings. An interesting result of the global fitting was that the reverse rate constant for the chemical transformation (*k*_–1_) had an estimated value that was not statistically different from zero. Parameter sensitivity analysis identified an upper boundary for *k*_–1_ of 0.1 s^–1^. Therefore, the internal (“on-enzyme”) equilibrium constant for the overall chemical transformation (*K*_int_ = *k*_1_/*k*_–1_) has a value of 1.54 × 10^2^ or greater.

We then examined the reaction of H186K. The multiple-turnover progress curve was best-described by a simplified kinetic mechanism (Scheme S2) that lumps the rate constants for the two paths of product release (Figure 4b; steps *k*_2_, *k*_3_, *k*_4_, and *k*_5_) into the single net rate constant *k*_P_. The obtained kinetic data (Table S5) reproduced the transient rate constant (eq S11), the burst magnitude (eq S12), and the *k*_ss_ (= *k*_cat_, eq S13) in excellent agreement with the corresponding parameters determined directly from the fit of the burst equation (eq S3 and Table S3). The microscopic rate constants (Table 1) reveal major differences between H186K and wild-type GFS in the value of *K*_int_, which is only 1.69 in the variant and reflects substantial increase (∼10^2^-fold) in the relative importance of the reverse chemical reaction (*k*_–1_ = 1.2 s^–1^) in H186K as compared to wild-type GFS. It is worth pointing out that the NADPH occupancy in the enzyme as-isolated was similar (∼45%) in H186K and wild-type GFS (Table S1). The result suggests that both enzymes bind NADPH tightly and there was no reason to assume that the low burst magnitude in the H186K reaction might have been caused by the portion of total enzyme unoccupied with substrate or coenzyme under the conditions used.

Lastly, we made an effort to apply global fitting of the kinetic mechanism to rationalize the transient-state KIE of 1.4 on the catalytic step of C-3″ epimerization. The proposed mechanism for the single-turnover conversion of substrate **3** (Scheme S3) involved a reversible step of epimerization, comprised of the forward and reverse rate constants *k*_e_ and *k*_–e_, respectively, followed by an irreversible step of reduction (*k*_1_). No unique solution was received from global fitting, and only the internal equilibrium for the epimerization step (*K*_int_ = *k*_e_/*k*_–e_ = ∼1–2) could be estimated. The estimates of the individual rate constants *k*_e_ and *k*_–e_ could vary in a broad range (50–1000 s^–1^) without compromising the quality of the fit as long as the value of *K*_int_ was constant. The transient-state KIE due to C-3″ deuteration of substrate **3** was explained from a corresponding decrease of *K*_int_ by at least a factor of ∼1.4. Note that the observation of normal KIE (>1) on the single-turnover transient rate constant *k*_st_ while no KIE was observed on *k*_cat_/*K*_m_ at the steady state is in line with the kinetic mechanism proposed. The KIE on *k*_cat_/*K*_m_ can be lowered by commitments to catalysis in the forward and reverse direction of the reaction.^49^ The KIE on *k*_st_ is unaffected by these commitments.^51,52^

### Transient Kinetic Analysis of Binding of GDP-l-fucose

The proposal of a rate-limiting release of GDP-l-fucose obtained from simulation studies (Figure 4b) motivated experiments to examine the kinetics of GDP-l-fucose binding directly. Results of equilibrium binding studies (Table S3; Figures S14 and S15) provided the basis for the stopped-flow measurement of GDP-l-fucose binding to the GFS complex with NADPH_4_, monitored by quenching of protein fluorescence. The results are listed in Figure 5a. Progress curves of fluorescence decrease upon mixing GDP-l-fucose to GFS/NADPH_4_ were described by single-exponential decay (eq S14) and the associated rate constant (*k*_obs_) exhibited hyperbolic dependence on the concentration of GDP-l-fucose **1a** (Figure 5b). If GDP-l-fucose binding was a simple (single-step) process, the dependence of *k*_obs_ on [product **1a**] would be expected to be linear. The results therefore suggest GDP-l-fucose binding in two steps where after the initial formation of encounter complex between GFS-NADPH_4_ and GDP-l-fucose, kinetic isomerization happens to yield the conformationally rearranged ternary complex (Figure 5c). For a structural interpretation of the kinetic isomerization, see the crystallographic evidence presented later. Equation S15 is derived for a binding model (Figure 5c) that assumes encounter complex formation in rapid equilibrium compared to the slower steps of conformational rearrangement. Fit of the data yielded a GDP-l-fucose release rate (*k*′_–1_ = 0.5 ± 0.1 s^–1^; Figure 5b) in suitable agreement with the relevant rate constant (*k*_5_) for the release of GDP-l-fucose from enzyme-NADPH obtained by kinetic simulation (Figure 4b). Of note, global simulation fit of the transient kinetic data of GDP-l-fucose binding gave similar and well-constrained values of the kinetic parameters of binding in two steps: *K*_d_ = 7 μM (range: 3–12 μM); *k*′_1_ = 0.6 s^–1^ (range: 0.3–0.8 s^–1^); *k*′_–1_ = 0.9 s^–1^ (range: 0.5–1.4 s^–1^). The individual association step parameters (*K*_d_ = *k*_off_/*k*_on_) could not be fully resolved; only lower bounds were estimated (*k*_on_ ≥ 2 μM^–1^s^–1^; *k*_off_ ≥ 11 s^–1^). Overall, these results strongly support the idea of rate limitation by dissociation of product **1a** and additionally demonstrate full consistency of results over different approaches to kinetic analysis.

**Figure 5:**
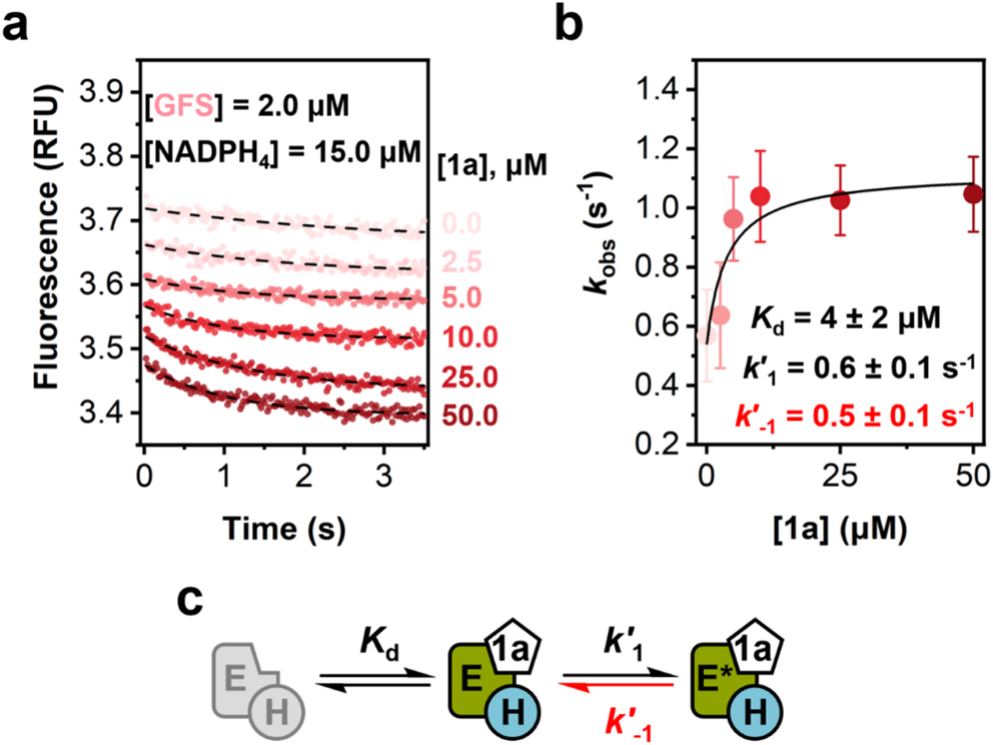
Transient kinetic analysis of the binding of GDP-l-fucose to the GFS complex with NADPH_4_. (a) Stopped-flow progress curves of the quenching of intrinsic protein fluorescence by GDP-l-fucose binding. The curves (RFU, relative fluorescence units) were recorded by mixing enzyme and NADPH_4_ solution with a solution of product **1a**. The conditions used (concentrations after mixing) are shown in the panel. A Tris buffer (10 mM, pH 8.0) with 25 mM NaCl was used. Averaged data are shown from *n* = 3 experiments at 37 °C. Data are shown in color, and dashed lines show their exponential fits (eq S14). Fluorescence traces are vertically offset from each other for clarity reason. (b) Concentration dependence of *k*_obs_ for GDP-l-fucose binding. Error bars show the standard deviation of triplicate measurements. Color is used to identify data (*k*_obs_) for the fluorescence traces in panel (a). The solid black line shows nonlinear fit with a model of two-step binding (eq S15). (c) Proposed model of binding of **1a** to the complex of GFS with NADPH.

### X-ray Structures of GFS Complexes with NADP^+^ and NADP^+^/GDP

Crystallographic studies of GFS were undertaken in search of a structural interpretation of the random kinetic mechanism and enzymatic rate limitation by GDP-l-fucose release. Structures of GFS complexes with NADP^+^ and NADP^+^/GDP were obtained at useable resolutions of 2.70 (PDB: 4BKP) and 2.75 Å (PDB: 4B8Z), respectively (Table S6). Both crystals contained two homodimers in the asymmetric unit. The subunits are arranged side to side, and their intersubunit interface is a four-helical bundle formed by the α3 and α5 helices of each subunit (Figure 6a). Each subunit adopts the characteristic SDR fold,^38,41,57^ composed of a Rossman-fold domain for nicotinamide coenzyme binding (residues 1–72, 83–113, 140–179, 220–242, and 287–299) and a smaller, predominantly α-helical domain (residues 73–82, 114–139, 180–219, 243–286, and 300–321) responsible for substrate binding (Figure 6b). The active site is in a cleft at the interface of the two domains (Figure 6b). The two active sites are separated one from another in the dimer structure (Figure 6b) and apparently function independently in catalysis. The active site is composed of a canonical SDR catalytic triad (Tyr143, Thr114, and Lys147) for C-4″ carbonyl reduction by NADPH, extended by two residues (Cys116 and His186) promoting C-3″/C-5″ epimerization (Figure 6b).

**Figure 6:**
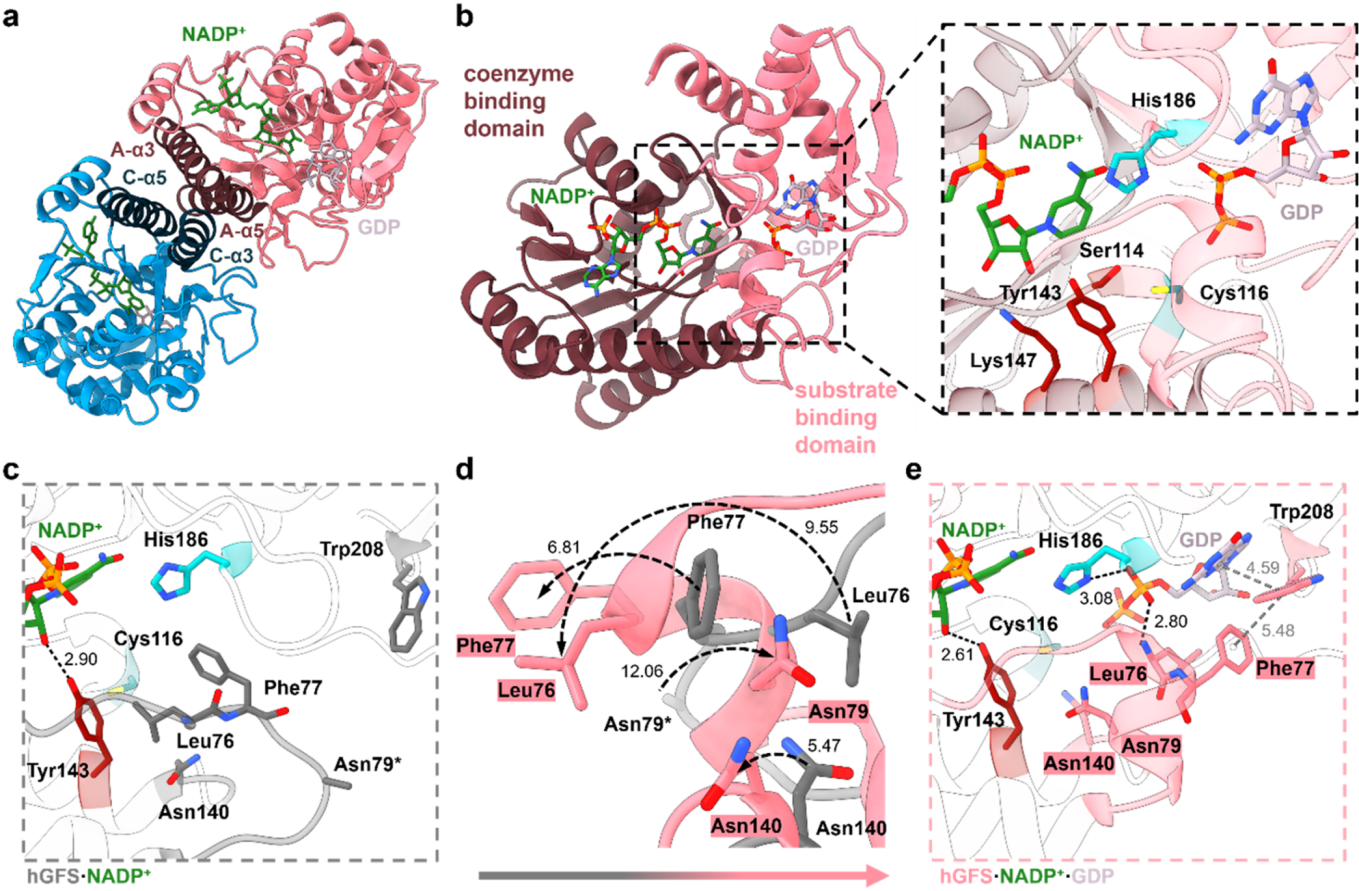
GFS complex structures with NADP^+^ (PDB: 4BKP) and NADP^+^/GDP (PDB: 4B8Z). (a) GFS functional homodimer (4B8Z) with elements of the four-helix bundle dimer interface highlighted (A-α3 and A-α5 in dark pink, C-α3 and C-α5 in dark blue). Note that the designations A–D of the four protein subunits in the asymmetric unit follow the structure deposited in the PDB (4B8Z). Functional dimers are formed between subunits A­(blue)/C­(pink) and B/D (not shown). (b) Two-domain protein fold of the GFS, revealing the Rossman-fold-like domain for NADPH binding (dark pink) and the mostly α-helical substrate binding domain (bright pink). A zoom into the active site is shown, with NADP^+^ (dark green), GDP (light purple), and catalytic residues indicated. (c) Binding pocket of GFS in complex with NADP^+^ (PDB: 4BKP), showing the conformationally flexible region in gray. The asterisk mark for Asn79 indicates the unassigned rotamer for this residue due to low electron density. (d) Superimposed structures (RMSD 1.424 Å) of GFS complexes with NADP^+^ (gray) and GDP/NADP^+^ (pink), showing loop-to-helix ordering and residue conformational changes for the sequence 74–82 and for Asn140. Dashed arrows indicate the movement of residues (Å). (e) Binding pocket of GFS in complex with GDP/NADP^+^, showing the conformationally flexible region in pink. Hydrogen bonds (Å) are indicated by black dashed lines with heteroatom distances indicated. Putative π–π stacking interactions (Å) are indicated by gray dashed lines based on interplanar distances.

The catalytic residues are contributed from both domains of the enzyme subunit. The configuration of the active site is well preorganized and is unchanged in the NADP^+^/GDP complex compared to the NADP^+^ complex (Figure S17). The binding pockets for NADP^+^ and GDP are well-separated from one another in the enzyme structure and appear to give independent access to each ligand for binding to the free enzyme, without the requirement of prior binding of the respective other ligand (Figures S18 and S19). The ligands are bound identically in all four enzyme subunits present in the asymmetric unit (Figure S20). GFS interactions with NADP^+^ are unaffected by the presence of bound GDP (Figures S21–S23), suggesting that binding of NADP^+^ is probably not cooperative with the binding of GDP. Binding of GDP involves induced fit of the enzyme binding pocket (Figure 6c–e). A prominent loop-to-helix transition for residues 74–82 results in enhanced compaction and structural preorganization of the extended active site (Figure 6d). The main-chain nitrogen of Leu76 establishes a strong hydrogen bond (2.80 Å) with the α-phosphate moiety of GDP (Figure 6e). The α-phosphate has an additional hydrogen bond with His186 in the immediate enzyme active site. Concerted position changes for Phe77 and Trp208 shape a binding pocket for the guanine moiety. Trp208 undergoes side chain rotation to develop stacking interactions with both the guanine and Phe77. The conformational change of Trp208 promotes further important interactions with GDP. The main-chain nitrogen of Trp208 interacts with the N-9 of the guanine, and the main-chain oxygen interacts with the 2-hydroxy group of the ribosyl moiety (Figure S19). Additionally, Lys194 forms a cation−π interaction with Trp208 and so can additionally interact with the O-6 of guanine. Trp208 and Phe77 close up the binding pocket toward the solvent, and it is significant that GDP could not be released in the absence of an “unbinding” conformational change of the two residues. This conformational rearrangement likely explains the kinetic isomerization step during binding of GDP-l-fucose described in the previous section. Asn79 also moves upon GDP binding to become part of the network of residues that form the binding pocket for the sugar residue. The rotamer state of Asn79 is not well-defined (i.e., probably quite flexible) in the NADP^+^ structure. Residues 138–140 change their conformation, resulting in Asn140 to orient toward the sugar binding pocket. In addition, Tyr143 moves closer toward the center of the active site, thereby shortening the hydrogen bond between its phenolic OH and the 2-OH of the NADP^+^ ribosyl from 2.90 to 2.61 Å. Finally, Arg215 and Lys282 coordinate the β-phosphate group of the GDP. The GFS complex structure with NADP^+^ and GDP-l-fucose (PDB: 4BL5) will be reported in a separate paper, but its comparison to the enzyme complex structure with NADP^+^ and GDP (PDB: 4B8Z) reveals that the l-fucosyl moiety did not induce conformational changes in addition to the ones already induced by GDP alone (Figure S24). We also note the report of Zhou *et al*.^58^ on the NADPH complex structure of the human GFS. Structural comparison shows that NADP^+^ and NADPH bind similarly and both structures involve conformational flexibility in the binding pocket for GDP-l-fucose (Figures S21, S25, and S26). The structures show positioning of the nicotinamide ring in a relatively hydrophobic binding pocket, which might be one factor of the preference of GFS for binding NADPH over NADP^+^ (Figure S27). The structural evidence just discussed has importance for the interpretation of the kinetic data because it suggests that the reverse direction of the conformational change likely represents the molecular origin of the slow release of GDP-l-fucose. Held in place by the interactions developed through the induced fit, the GDP moiety is not ready to dissociate.

## Discussion

### The kinetic Mechanism of GFS and Its Change in the H186K Variant

Several lines of evidence indicate a random kinetic mechanism of human GFS (Figure 4b; mechanism b). Random binding of substrates is suggested by KIE studies and fluorescence titration analyses, and it receives strong support from the X-ray crystal structures of enzyme complexes with NADPH and NADPH/GDP. Random release of the products is suggested from the analysis of burst kinetics in multiple-turnover stopped-flow progress curves of NADPH conversion.

Global fitting of the data provides estimates of the microscopic rate constants. The rate constants are validated by their excellent agreement with parameters determined directly from the experiments. The overall chemical transformation (*k*_1_ = 15.4 s^–1^) is 6-fold faster than the total net product release (*k*′_2,3_). The ternary product complex (enzyme/NADP^+^/GDP-l-fucose) partitions 2.2-fold faster into the binary complex with NADP^+^ than into the complex with GDP-l-fucose. The rate constants for the release of NADP^+^ (26 s^–1^) and GDP-l-fucose (12 s^–1^) have similar values as *k*_1_. The *k*_1_ is 15-fold faster than the slowest step of the mechanism, which is release of GDP-l-fucose (**1a**) from the abortive enzyme complex with NADPH (*k*_5_). Transient kinetic analysis of binding of GDP-l-fucose to the GFS complex with NADPH_4_ supports slow release of product **1a** and suggests that the release rate is governed by kinetic isomerization before the actual dissociation. Structurally, the kinetic isomerization is interpreted as a conformational rearrangement that involves stacking interactions of Trp208 to the guanine moiety of the ligand. Release of NADP^+^ from the abortive complex with substrate **3** (*k*_4_) is relatively much (25-fold) faster than the release of GDP-l-fucose. The enzyme complex with NADPH/GDP-l-fucose, therefore, accumulates at steady state and accounts for ∼85% of the total enzyme present. Fluorescence titration analysis shows that GDP-l-fucose (**1a**) binds to the enzyme complex with NADPH_4_ about as tightly as substrate **3** does (Figure S15 and Table S3). We discuss below that the enzyme complex with NADPH and GDP-l-fucose may be physiologically relevant and represents the enzyme form targeted by inhibitors.

Histidine-to-lysine replacement of the general catalytic acid for C-3″/C-5″ epimerization is largely conservative in terms of the GFS function. The *k*_cat_ of H186K is decreased only weakly (5.6-fold) compared to wild-type GFS, and enzyme specificity in product formation is retained fully. The rate constant ratio for chemical transformation and overall product release has a value of ∼6 in both H186K (*k*_1_/*k*_P_) and wild-type GFS (*k*_1_/*k*′_2,3_). The main difference between the two enzymes lies in the internal equilibrium constant for the overall chemical step (*K*_int_ = *k*_1_/*k*_–1_). The value of *K*_int_ is 1.69 in H186 K, whereas in wild-type GFS only a lower boundary of ∼10^2^ can be given because the estimated value of *k*_–1_ is not statistically different from zero.

Interpretation of the large change in *K*_int_ between wild-type and H186K enzymes must consider that *k*_1_ and *k*_–1_ are not rate constants for an elementary reaction step. Although *k*_1_ and *k*_–1_ are determined from progress curves of NADPH conversion, they are composites of the steps of C-3″/C-5″ epimerization and C-4″ carbonyl reduction. The absence of KIE on steady-state parameters (*k*_cat_, *k*_cat_/*K*_m_) in reactions with a deuterium-labeled substrate ([3″-^2^H]; [5″-^2^H]) or NADPH suggests that in both wild-type GFS and H186K, the chemical steps are relatively fast compared to other physical steps, implying the possibility that each chemical step comes to internal equilibrium on the enzyme before the products are released. Transient-state KIEs obtained in single-turnover stopped-flow studies of the wild-type enzyme provide additional kinetic insight into the chemical steps of GFS catalysis. The results support the notion that both epimerization steps are faster considerably than the reduction step.

By reference to external equilibria for sugar epimerization,^40,59^ one would expect the internal equilibrium for the epimerization steps to lie rather in the middle (*K*_int_ = ∼1). Indeed, fitting of the single-turnover progress curves of the wild-type reaction suggested *K*_int_ for the overall epimerization (= *k*_e_/*k*_–e_; Scheme S3) to be close to unity. The internal equilibrium for the reduction step is expected to lie rather on the side of the product. However, *K*_int_ values in a broad range between ∼1 and 10^2^ have been reported for different dehydrogenases/reductases catalyzing the carbonyl-alcohol interconversion by NAD­(P).^60–63^ The wild-type GFS is remarkable for its ability to drive the chemical steps of its reaction forward. Enzymatic strategies of “equilibrium pull” to C-3″/C-5″ epimerization might involve a rapid return to the original protonation state of the general base/general acid-catalytic residues after the reaction, thus rendering epimerization in the reverse direction unfavorable under the conditions used (Figure 1b).

The proposed GFS mechanism involves Cys116 as the general base and His186 as the general acid in the epimerization of *both* C-3″ and C-5″ (Figure 1b), implying the need for the cysteine to become deprotonated and the histidine to become reprotonated after the C-3″ epimerization so that the C-5″ epimerization can proceed. Conformational changes that accompany catalysis (e.g., changes in the sugar ring pucker) could represent an additional feature of forward pull in the reaction of wild-type GFS. The direct involvement of His186 in the immediate catalysis to C-3″/C-5″ epimerization supports the idea that shift in *K*_int_ for the H186K variant might result from the effect of the site-directed substitution on the on-enzyme equilibrium of one or both epimerization steps (Figure 1b).

Studying the GFS,^34^ Menon *et al*. analyzed the conversion of substrate **3** in the absence of NADPH or in the presence of NADP^+^, that is, under conditions that preclude the C-4″ carbonyl reduction. The fully C-3″/C-5″ epimerized product was found, while singly C-3″ or C-5″ epimerized products were not detected. These findings support the notion of efficient coupling of the two steps of epimerization by GFS. Structurally in the human GFS, His186 and, by extension, Lys186 appear not to play a direct role in C-4″ carbonyl reduction by NADPH and would therefore seem unlikely to affect the associated internal equilibrium. However, structural change of the extended active site can also affect the internal equilibrium for reduction, as shown with other enzymes,^60,63^ precluding a definite conclusion for GFS at this stage. Further research might combine experimental studies with quantum mechanics and molecular mechanics simulations to explore mechanistic details of the reactions catalyzed by wild-type and H186K forms of the human GFS. The steady-state and transient-state KIE data can be a useful point of departure to explore the enzymic mechanism with molecular simulations.

### Implications for the Catalytic Mechanism

Evidence from detailed kinetic characterization and KIE studies of wild-type GFS and site-directed variants thereof enables the assignment of specific tasks in catalysis to individual residues in the human GFS active site. We recall at this point that the native GFS coordinates precisely the steps of epimerization (C-3″ before C-5″) to the subsequent reduction step so that only a single isomeric product is released (see refs 31,34–37 for studies of the GFS).

In the human GFS, Cys116 is suggested as the general base catalyst for C-3″ epimerization. Complete removal of its function in C116A destroys the activity to below the detection limit. Replacing the Cys116 with serine renders abstraction of the C-3″ proton rate-determining in a variant that has largely (∼10^3^-fold) lost the original enzyme activity. Two lines of evidence support the conclusion. Multiple-turnover stopped-flow progress curves of C116S are linear, implying that product release is no longer the slowest step of the reaction. Reaction of C116S with [3″-^2^H]-substrate **3** involves the appearance of a normal KIE (^D^ *k*_cat_ = 1.6). In wild-type GFS and other variants (Y143F, H186K) the analogous ^D^ *k*_cat_ was ∼1.0, implying that the KIE was masked by other steps in the kinetic mechanism. Formation of GDP-d-altrose (**4**) in ∼70% of total product released by conversion of substrate **3** shows that in the C116S reaction, premature reduction of the C-3″ epimerized GDP-4″-keto-6″-deoxy-d-altrose intermediate largely outcompetes further epimerization at C-5″. While GDP-d-altrose (**4**) formation by C116S indicates a substantial slowdown of the C-5″ epimerization caused by the site-directed substitution, the ^D^ *k*_cat_ for the reaction with [5″-^2^H]-substrate **3** was not different from unity. Partitioning of the C-3″ epimerized GDP-4″-keto-6″-deoxy-d-altrose intermediate between C-5″ epimerization and reduction may reduce a normal KIE on the proton abstraction from C-5″ to the value of ∼1.0 observed in the experiment. Note that the formation of GDP-d-altrose does not involve the abstraction of proton from C-5″. Significantly, there is a small normal KIE (^D^ *k*_cat_ = 1.12) in the C116S reaction that arises from the use of [4*S*-^2^H]-NADPH.

Complete loss of activity to below the detection limit in H186A shows the crucial importance of His186 for GFS function. Role of general acid in catalysis to C-3″ and C-5″ epimerization was proposed for histidine (Figure 1). Comparison of the kinetic properties and the KIE data for the wild-type enzyme and H186K suggests that the human GFS may be rather permissive with the structure of the protonic residue at position 186. In GFS catalysis to C-3″/C-5″ epimerization, the initial deprotonation of the carbon to generate an enolate/enol intermediate is the most challenging task. Reprotonation of the incipient enolate/enol from the opposite face is expected to proceed more easily in comparison. In sugar nucleotide decarboxylases related to GFS by a common membership to the SDR superfamily, protonation of an analogous C-5″ enolate/enol is achieved even without catalytic facilitation from a general acid residue on the enzyme.^64^ Protonation of the enolate/enol may, however, constitute an important factor of the internal equilibrium of the epimerization, assuming that the p*K*_a_ of an enzyme-stabilized enolic intermediate could be close to the p*K*_a_ (= ∼10) of the lysine side chain of H186K. Overall, therefore, His186 is suggested as the general catalytic acid for epimerization at both C-3″ and C-5″. The general catalytic base in both steps is thus Cys116.

Tyr143 is suggested as a proton donor for the C-4″ carbonyl reduction as the last of the three chemical steps. Replacement of Tyr143 with phenylalanine results in chemical reduction becoming the rate-determining step. The conclusion is supported by evidence that the reaction of Y143F involves a large KIE (^D^ *k*_cat_ = 3.9) arising from the use of [4*S*-^2^H]-NADPH; and the multiple-turnover stopped-flow progress curves of NADPH consumption are linear with a slope consistent with the *k*_cat_. The absence of KIE from the use of [3″-^2^H]- or [5″-^2^H]-substrate **3** by Y143F suggests that the steps of epimerization were not as strongly affected by the site-directed substitution as the reduction step. The proposed catalytic mechanism of the human GFS is effectively same as that of the enzyme from (Figure 1b).^31^ Important novelty from the study of the human enzyme is the clarity of how the catalytic steps are integrated into the overall kinetic mechanism of the enzyme (Figure 4b,4d). Tight coupling of the epimerization steps to the reduction step, with the result of *K*_int_ lying far on the side of product **1a**, was an insight only revealed by combining mechanistic and kinetic studies.

### Implications for the Inhibition of Human GFS

Product release *via* an abortive (dead-end) ternary complex of the enzyme with NADPH/GDP-l-fucose may be relevant physiologically. The metabolic concentration of NADPH (20–250 μM) is vastly saturating (*K*_m_ = 0.75 μM) and exceeds that of NADP^+^ in large amount (≥5 to 10^2^-fold).^65–67^ The human GFS binds NADPH_4_ (*K*_d_ = 0.3 μM; Table S3) more tightly (27-fold) than it binds NADP^+^. The preference for binding the reduced coenzyme compared to NADP^+^ is similar to GFS.^34^ The cellular concentration of GDP-4″-keto-6″-deoxy-d-mannose (**3**) has not been reported to the best of our knowledge. GDP-d-mannose (**2**) and GDP-l-fucose (**1a**) accumulate in mammalian cells to concentrations of approximately 5–40 and 1–20 μM, respectively.^68–70^ It is reasonable to assume, therefore, that the concentration of substrate **3** might at least approach the GFS *K*_m_ of 1.7 μM. Tuning the enzyme *K*_m_ to a value close to,^71,72^ or even lower than,^73^ the physiological substrate concentration (while *k*_cat_/*K*_m_ is being kept constant) represents a well-known principle for the evolutionary maximization of the enzymatic rate in cellular metabolism.^71,72^ Optimality of enzyme utilization in bimolecular reactant systems depends on both the substrate and product concentrations. Sahin *et al*.^74^ discuss that the random mechanism of substrate binding is optimal over any other ordered mechanism under physiological conditions. Random release of the products could be an additional principle used by the GFS to enhance the overall rate when the dissociation of one product (i.e., GDP-l-fucose) is rate-determining.

The inhibition constant of GDP-6″-alkynyl-l-fucose (Scheme S4) was determined as 2.9 μM for human GFS.^27^ Despite the complex kinetic mechanism of the enzyme (Figure 4b), analogues of GDP-l-fucose will be competitive inhibitors with respect to substrate **3** binding to enzyme-NADPH. Studies of GFS^34^ suggest that the substrate/inhibitor binding affinity is governed mostly by the GDP moiety: the *K*_i_ of GDP-l-fucose is 55.3 μM and that of GDP is 60.5 μM; these findings are in line with the structural evidence of this study, showing that GDP binding is sufficient to induce binding pocket rearrangements for substrate/product binding. However, as can be seen from Figure 4d, it takes about ∼1 s and 3.5 turnovers of the enzyme until the predominant GFS complex with NADPH and product **1a** was fully developed. Because the abortive ternary enzyme complex is unable to bind to substrate **3** or inhibitor before it has released GDP-l-fucose, and this release happens at a slow rate as we have shown, the degree of *in vivo* inhibition of the GFS, when both NADPH and substrate **3** are saturating, can be considerably lower than predicted from an inhibition constant (*K*_i_) evaluated in conventional assays at a low substrate concentration. Simulations with mechanism b (Figure S28) show that the inhibition of GDP-6″-alkynyl-l-fucose at concentrations of the reported *K*_i_ is reduced by ∼10%, compared to the effect expected from *K*_m_ and *K*_i_ when assuming a 5 μM concentration of substrate **3** (30% instead of 40% inhibition) based on the lowest reported concentration of GDP-d-mannose and requires about ∼1 s (i.e., 2–3 enzyme turnovers) to develop fully.

Summarizing, we present evidence from kinetic, mechanistic, and structural characterization that elucidates the complex reaction of the human GFS. We show how the three catalytic steps of the enzymatic transformation are integrated into an overall framework of kinetic mechanism. The proposed kinetic mechanism is likely relevant for enzyme activity under physiological reaction conditions and provides a refined view on the effect of small-molecule inhibitors, such as the analogues of GDP-l-fucose that compete with GDP-4″-keto-6″-deoxy-d-mannose for binding to the enzyme.

## Supplementary Material



## Notes

Experimental methods used; structures of compounds described in this work (Figure S1); enzyme purification, biochemical, and sequence analysis (Figures S2–S4 and Table S1); substrates and deuterated analogues characterization and synthesis (Figures S5–S8 and Scheme S1); primary deuterium kinetic isotope effect measurements (Figures S9 and S10); multiple-turnover kinetic analysis (Figure S11, Schemes S2 and S3, Table S2); coenzyme analogue characterization and synthesis (Figures S12 and S13); equilibrium binding measurements (Figures S14 and S15, Table S3); quantification of enzyme-bound ligands at steady state (Figure S16); microscopic rate constants from global simulation fitting (Tables S4 and S5); structural analysis (Figures S17–S27 and Table S6); inhibitor structure and inhibition simulation (Figure S28 and Scheme S4); nucleotide and protein sequence of the GFS enzyme with corresponding expression vector (Table S7); and oligonucleotide primers (Table S8) (PDF)
