# Supplementary material for: Kinetic Dissection of the Reaction of Human GDP-l-Fucose Synthase
Source: ACS Catal. 2025 Jul 29;15(16):13872–85. doi: 10.1021/acscatal.5c02722 (PMC12362431; doi:10.1021/acscatal.5c02722)
Supplement: Supplementary file 1 [file cs5c02722_si_001.pdf]

Supporting Information

Kinetic dissection of the reaction of the human GDP-L-fucose synthase

Denis Smyshliaev,[1] *Martin Pfeiffer,*[1,2] *Udo Oppermann,*[3,4] *Bernd* Nidetzky[1,2,*]

[1]Institute of Biotechnology and Biochemical Engineering, Graz University of Technology, NAWI Graz, Petersgasse 12, A-8010 Graz, Austria

[2]Austrian Centre of Industrial Biotechnology (acib), Krenngasse 37, A-8010 Graz, Austria

[3]Botnar Research Centre, Nuffield Department of Orthopaedics, Rheumatology and Musculoskeletal Sciences, National Institute of Health Research Oxford Biomedical Research Unit (BRU), University of Oxford, OX3 7LD Oxford, United Kingdom

[4]Oxford Centre for Translational Myeloma Research, University of Oxford, OX3 7LD Oxford, United Kingdom

* Corresponding author, e-mail: bernd.nidetzky@tugraz.at

Contents

MATERIALS AND METHODS 3
- Materials 3
- Site-directed mutagenesis 4
- Expression and purification of GFS and site-directed variants thereof 4
- Protein concentration determination 5
- Analysis and quantification of enzyme-bound NADPH and $NADP^+$ 5
- Synthesis of GDP-D-mannose 6
- Synthesis of GDP-4″-keto-6″-deoxy-D-mannose and deuterated analogues 7
- Synthesis of GDP-L-fucose 7
- Synthesis of NADPH and [4*S*-^{2}H]-NADPH 8

Synthesis of the NADPH analog $NADPH_4$ 8
Ligand-free enzyme preparation 9
Primary kinetic isotope effect measurements 10
Rapid-mixing stopped-flow kinetic analysis 10
Analysis of multiple- and single-turnover kinetics 12
Spectrofluorometric analysis of ligand binding 13
Kinetic simulation and parameter fitting 13
Analysis and kinetic simulation of transient binding kinetics 15
Quantification of enzyme-bound NADPH and $NADP^+$ at steady state of reaction 16
Analytical methods 17
Crystallization and data collection 18
SUPPORTING FIGURES 20
SUPPORTING SCHEMES 47
SUPPORTING TABLES 49
SUPPORTING REFERENCES 56

MATERIALS AND METHODS

Materials

NADPH (>98%) and NADP$^+$ (>98%) were from Carl Roth GmbH (Karlsruhe, Germany). GTP (>99%) was from Carbosynth (Compton, UK). Deuterium oxide (^2H_2O, 99.96%) was from Euriso-Top (Saint-Aubin Cedex, France). ATP (>99%), D-mannose (>98%) and [1-^{2}H]-D-glucose (98% ^{2}H) were from Sigma Aldrich (Darmstadt, Germany). [3-^{2}H]-D-mannose (98% was from Omicron Biochemicals (South Bend, IN, USA). The *E. coli* expression strains (Rosetta 2(DE3) and BL21(DE3)), Q5 reaction buffer, dNTP, and Q5 High Fidelity DNA Polymerase were from New England BioLabs GmbH (Frankfurt/Main, Germany). *Dpn*I restriction enzyme was from Thermo Fisher Scientific, Inc. (Altham, MA, USA). Oligonucleotide primers were from Sigma-Aldrich. For plasmid isolation, Wizard Plus SV Minipreps DNA Purification System (Promega Corporation, Madison, WI, USA) was used. DNA sequencing was done at LGC Genomics (Berlin, Germany). The enzymes used in this study for sugar nucleotide synthesis (GenBank/NCBI reference sequence in brackets) were the following: *N*-Acetyl hexosamine 1-kinase from *Bifidobacterium longum* NahK[1,2] (E8MF12); mannose-1-phosphate guanylyltransferase from *E. coli* ManC[3] (ACT43784.1); inorganic pyrophosporilase from *E. coli* iPPase[3] (AAB88583.1); GDP-mannose-4,6-dehydratase from *Homo sapiens* GMD[2] (AAC24501); L-fucokinase/GDP-L-fucose pyrophosphorylase from *B. fragilis Bf*FKP[4] (Q58T34). These enzymes were purified and stored as described in the respective references. Glucose dehydrogenase GDH from *Pseudomonas sp.* (≥200 U/mg) was from Sigma Aldrich. Expression vector (pNIC28_*hgfs*) containing the gene of GDP-L-fucose synthase (GeneID: 7264) from *Homo sapiens* was provided by Udo Opperman (SGC, Oxford, United Kingdom). The protein sequence can be found in **Table S7**. GFS variants C116A, C116S, H186A, H186K and Y143F were generated by site-directed mutagenesis.

Site-directed mutagenesis

The DNA template used was pNIC28_*hgfs*. A two-stage PCR method with a modified protocol of the QuickChange™ Site-Directed mutagenesis[5] was used. Two separate PCR reactions, using either forward (fwd) or reverse (rev) primer (**Table S8**), were performed for each mutation in the first step. The reaction profile consisted of a preheating step at 98 °C for 15 s, followed by 10 cycles of linear PCR amplification (98 °C, 7 s/50 °C, 15 s/ 72 °C, 420 s). In the second step, reaction products were combined 1:1 (by volume) and amplification was continued for additional 15 cycles of exponential DNA amplification (98 °C, 7 s/55 °C, 15 s/72 °C, 420 s). The parental template was removed by *Dpn*I digestion of the final PCR product, which was followed by *E. coli* Top10 cells transformation with plasmid by electroporation. Single-colony transformants were selected on agar plates containing either 0.05 mg/mL kanamycin. Plasmids were amplified in *E. coli* Top10 cells, isolated and sent for sequencing at LGC Genomics. The verified constructs were used for the transformation of *E. coli* BL21(DE3) cells.

Expression and purification of GFS and site-directed variants thereof

E. coli Rosetta 2(DE3) expression strain was transformed with the expression vector encoding GFS or a site-directed variant thereof (plasmid vector pNIC28). Electroporation was used. Transformants were selected over 16 h at 37 °C on 0.05 mg/mL kanamycin LB-agar plates. Enzymes were expressed in 1 L baffled shaken flasks at 37 °C and 110 rpm using 250 mL LB-media containing 0.05 mg/mL of kanamycin. Inoculation was to an initial OD_{600} of 0.1. At an OD_{600} of ~1.0, the temperature was decreased to 18 °C and gene expression was induced with 0.2 mM IPTG (isopropyl β-D-1-thiogalactopyranoside) for 20 h. Cells were harvested by centrifugation at 2800 × g at 4 °C for 20 min using a Sorvall RC-5B refrigerated superspeed centrifuge (Du Pont Instruments, Newtown, CT, USA). The supernatant was discarded, and

the pellet was suspended in 30 mL buffer A (50 mM 4-(2-hydroxyethyl)-1-piperazineethanesulfonic acid (HEPES), 125 mM NaCl, 20 mM imidazole, pH 7.4). Cells were disrupted using sonication Fisherbrand Sonic Dismembrator, model Ultrasonic Processor FB-505 (Fisher Scientific, Vienna, Austria) on ice and the cell-free supernatant was recovered by centrifugation for 40 min at 4 °C and 21130 × g. The pre-treated cell lysate (25 mL) was loaded on a 2 × 5 mL HisTrap FF column (GE Healthcare, Little Chalfont, UK), equilibrated with buffer A and mounted on an ÄKTA start (GE Healthcare) system. Protein was eluted using an imidazole gradient from 0% to 80% buffer B (50 mM HEPES, 125 mM NaCl, 500 mM imidazole, pH 7.4). The temperature was 10 °C and a flow rate of 3 mL/min was used. Fractions containing the target protein were pooled, concentrated and buffer-exchanged with Vivaspin Turbo 15 (30000 MWCO; Sartorius AG, Göttingen, Germany) at 5975 × g and 4.0 °C. Molecular mass and purity of the proteins were confirmed by SDS-PAGE (**Figure S3**). Enzymes (~34 mg protein/mL) were stored at -20 °C until further use. GFS enzyme preparations were stable for at least 18 weeks.

Protein concentration determination

Protein concentrations were determined photometrically using a DS-11 Spectrophotometer (DeNovix Inc. Wilmington, DE, USA) at 280 nm. Molar extinction coefficients were calculated by ProtParam (https://www.expasy.org). GFS (43890 $M^{-1}cm^{-1}$, 37759 Da), GMD (54320 $M^{-1}cm^{-1}$, 42468 Da), NahK (27515 $M^{-1}cm^{-1}$, 39902 Da), ManC (60445 $M^{-1}cm^{-1}$, 52611 Da), iPPase (23045 $M^{-1}cm^{-1}$, 19703 Da).

Analysis and quantification of enzyme-bound NADPH and $NADP^+$

The content of protein-bound NADPH and $NADP^+$ was determined for GFS and the site-directed variants thereof. The purified enzymes (50 µL, 440 µM) were denatured with 50 µL

of methanol using incubation for 3 h at room temperature. The precipitated protein was removed by centrifugation (16100 × g, 80 min, 4 °C). The supernatant was collected and the amount of released NADPH and $NADP^+$ was analyzed on HPLC based on linear calibration (**Figure S22**). The amount of released NADPH/$NADP^+$ was calculated by dividing the concentration of released NADPH or $NADP^+$ by the total molar enzyme concentration (subunit; 220 μM) used in the experiment (**Table S1**).

Synthesis of GDP-D-mannose

The reaction mixture (5.0 mL, pH 7.5) contained 200 mM D-mannose, 200 mM $MgCl_2$, 220 mM ATP, 220 mM GTP, to which enzymes were added in concentrations of 100.0 μM NahK, 75.5 μM ManC, and 14.0 μM iPPase (**Scheme S3a**). Incubation was for 24 h at 25 °C and the reaction was monitored by thin-layer chromatography and HPLC to ensure that it reached completion. Proteins were removed using Vivaspin Turbo 15 (10000 MWCO; Sartorius AG) and the filtrate was diluted 20-times to a final volume of 100 mL. Diluted filtrate was split into 20 mL fractions and put to a HiPrep DEAE-FF column (16 mm × 100 mm, XR 16/10; GE Healthcare) prepacked with DEAE Sepharose Fast Flow, equilibrated in doubly distilled H_2O and mounted onto an ÄKTA FPLC system (GE Healthcare). Compound was eluted using a NH_4HCO_3 gradient from 0% to 100% AEX buffer (250 mM NH_4HCO_3, pH 8.0) and monitored at 260 nm. Fractions containing free sugar nucleotide were pooled and concentrated under reduced pressure (20 mbar, 40 °C) to a total volume of 10 mL using a rotary evaporator (Laborta 500-efficient; Heidolph Instruments, Schwabach, Germany), and were lyophilized afterwards. Purity of the synthesized GDP-D-mannose was confirmed using ^{1}H-NMR.

Synthesis of GDP-4″-keto-6″-deoxy-D-mannose and deuterated analogues

The reaction mixture (2.0 mL) contained 10 mM GDP-D-mannose, 95 µM of hGMD in 10 mM Tris buffer (pH 7.0, 50 mM NaCl added). The synthetic reaction is shown in **Scheme S1a**. Incubation was for 4 h at 30 °C and the reaction was monitored by HPLC to ensure that it reached completion. Protein was removed using Vivaspin Turbo 15 (30000 MWCO; Sartorius AG). The filtrate was applied to a size exclusion column (16 × 1000 mm; XR 16/100; GE Healthcare) packed with Sephadex G10 (exclusion limit <700 Da) equilibrated in doubly distilled H_2O and mounted onto an ÄKTA GO system (GE Healthcare). Compound elution was performed using doubly distilled H_2O and monitored at 260 nm. Fractions containing glycerol-free product were collected, pooled and concentrated under reduced pressure (20 mbar, 40 °C) to a total volume of ~1 mL using a rotary evaporator (Laborta 500-efficient; Heidolph Instruments) and were lyophilized afterwards. The C-3″-labeled substrate **3** was synthesized in the identical manner as the unlabeled substrate **3**, except that [3-^{2}H]-D-mannose was used for synthesis (**Scheme S1b**). The C-5″-labeled substrate **3** was synthesized in an identical manner as the unlabeled compound, except that the GMD-catalyzed step was carried out in ^2H_2O phosphate buffer (25 mM, p^{2}H 7.5; **Scheme S1c**). Structure and purity of the synthesized substrate **3**, [3″-^{2}H]- and [5″-^{2}H]-**3** were shown using HPLC and ^{1}H NMR, where >95% of the deuterated substrates contained a single deuterium atom (**Figures S5–S6** and **S8a**).

Synthesis of GDP-L-fucose

The reaction mixture (5.0 mL, pH 7.5) contained 100 mM L-fucose, 50 mM $MgCl_2$, 120 mM ATP, 120 mM GTP, to which enzyme was added in a concentration of 50 µM *Bf*FKP (**Scheme S1d**). Incubation was for 24 h at 25 °C and the reaction was monitored by HPLC to ensure that it reached completion. Protein was removed using Vivaspin Turbo 15 tubes (10000 MWCO; Sartorius AG) and the filtrate was diluted 20-times to a final volume of 100 mL. Purification

was carried out as previously described for GDP-D-mannose (see the earlier section). Purity of the synthesized GDP-L-fucose was confirmed using ^{1}H-NMR. (^2H_2O, 500 MHz): δ = 8.04 (s, 1H, H8), 5.87 (d, J=6.0 Hz, 1H, H1′), 4.85 (t, J=8.0 Hz, 1H, H1″), 4.71 (q, J = 5.7 Hz, 1H, H2′), 4.46 (dq, 1 H, H3′), 4.29 (dq, 1H, H4′), 4.14 (m, 1H, H5′), 3.71 (q, 1H, H5″), 3.65 (d, J = 3.9 Hz, 1H, H5″), 3.58 (m, 1H, H3″), 3.49 (dd, J = 10.0, 7.6 Hz, 1H, H4″), 1.16 ppm (d, J = 6.5 Hz, 3H, H6″).

Synthesis of NADPH and [4*S*-^{2}H]-NADPH

NADPH and [4*S*-^{2}H]-NADPH were obtained by reduction of $NADP^+$ (10 mM) with D-glucose or [1-^{2}H]-D-glucose (10 mM) catalyzed by GDH from *Pseudomonas sp.* (0.01 mg/ml) in 25 mM Tris buffer (containing 125 mM KCl, pH 8.5; **Scheme S1e**). The pH was controlled at 8.5 during the reaction (30 °C, 600 rpm in a Thermomixer Comfort, Eppendorf, Hamburg, Germany). Protein was removed using Vivaspin Turbo 15 (30000 MWCO; Sartorius AG). The obtained compounds were used directly after enzyme removal in KIE experiments. Control measurements confirmed that the synthetically prepared, non-purified NADPH did not affect steady-state kinetic parameters, which were identical to those obtained using commercial, high-purity NADPH (>98%; Carl Roth GmbH). The deuterium content of the obtained [4*S*-^{2}H]-NADPH was determined by ^{1}H NMR to be >98% (**Figures S7**). The $NADP^+$ content <1% was confirmed by HPLC (**Figures S8b**).

Synthesis of the NADPH analog $NADPH_4$

1″,4″,5″,6″-Tetrahydro-NADPH ($NADPH_4$) was obtained by catalytic hydrogenation of 110 mg of NADPH in 10 mL doubly distilled H_2O. The reaction solution was pumped through a HCube® system (Thales Nanotechnology Inc., Budapest, Hungary), with CatCart® cartridge containing 10% Pd/C (Thales Nanotechnology Inc.). The parameters used were as follows:

1.0 mL·min^{-1}, full H_2-mode (1 bar, >99%), 30 °C. The reaction progress was monitored by measuring absorbance spectra using a Varian Cary 50 UV-VIS Spectrophotometer (Varian Inc., Palo Alto, CA, USA) until the absorbance ratio 266:288 nm was 1.1, which indicates full conversion. The obtained product solution was put to a HiPrep DEAE-FF column (16 mm × 100 mm, XR 16/10; GE Healthcare) prepacked with DEAE Sepharose Fast Flow, equilibrated in doubly distilled H_2O and mounted onto an ÄKTA FPLC system (GE Healthcare). Compound was eluted using NH_4HCO_3 gradient from 0% to 100% AEX buffer (250 mM NH_4HCO_3, pH 8.0) and monitored at 260 nm. Fractions containing free $NADPH_4$ were pooled and concentrated under reduced pressure (20 mbar, 40 °C) to a total volume of 0.5 mL using a rotary evaporator (Laborta 500-efficient; Heidolph Instruments), and were lyophilized. The final yield was 100.7 mg (91.5%). Purity and structure of the synthesized $NADPH_4$ were confirmed spectrophotometrically and with ^{1}H-NMR (**Figures S12** and **S13**).

Ligand-free enzyme preparation

The purified wild-type enzyme (3 mL, 0.6 mM) was incubated for 2 h at room temperature with 10.0 mM substrate **3**. The incubation continued until all enzyme-bound NADPH was oxidized. The enzyme solution was immediately transferred into a Vivaspin Turbo 15 (30000 MWCO; Sartorius AG) and centrifuged at 4 °C (2880 × g) until 0.5 mL of the original volume remained. The enzyme was then washed 5 times with 10 mL of ice-cold buffer (50 mM HEPES, 125 mM NaCl, pH 7.4). The enzyme was subsequently concentrated to 0.9 mM in 1.5 mL and aliquots of 100 µL stored in -20 °C until further use. Absence of NADPH and $NADP^+$, as well as substrate **3** and product **1a**, in ligand-free GFS was confirmed by HPLC.

Primary kinetic isotope effect measurements

The reactions (500 µL) were performed in 10 mM Tris buffer (containing 25 mM NaCl, pH 8.0) without agitation (37 °C). Initial rates were determined at varying concentration of substrate **3** (or deuterated analogs thereof) in the range 0.001–0.050 mM and at varying NADPH ([4*S*-^{2}H]-NADPH) concentration (0.002–0.030 mM). Reactions were initiated by adding 10 µL of stock enzyme solution (0.06 µg wild-type, 11.5 µg C116S or Y143F, and 0.6 µg H186K) to the reaction mixture (490 µL). Initial rates were determined from the NADPH consumed in the enzymatic reaction, by absorbance at 340 nm (ε_{340} = 6.22 mM^{-1}cm^{-1}) using Varian Cary 50 UV-VIS Spectrophotometer (Varian Inc.). Under the conditions used, the decrease in absorbance was linear over 1–7 min.

A set of KIEs ($^{D}k_{cat}$, $^{D}k_{cat}/K_m$) were determined for each enzyme using [^{2}H]-labeled and unlabeled substrate **3** or NADPH. Michaelis-Menten curves were measured in triplicates and globally fitted to Eq. S1 using OriginPro 9.6 software (OriginLab Corporation, Northampton, MA, USA). Reported standard deviations were derived from three independent fits.

$$\frac{\mathrm{v}}{[\mathrm{E}]} = \frac{k_{\mathrm{cat}}[\mathrm{S}]}{[K_{\mathrm{m}}(1+\mathrm{F}(\mathrm{E}_{\mathrm{v/k}}-1))+[\mathrm{S}](1+\mathrm{F}(\mathrm{E}_{\mathrm{v}}-1))]} \qquad \text{S1}$$

$E_{v/k}$ and E_v are the KIEs on k_{cat}/K_m and k_{cat}, respectively. F is the deuterium fraction in the deuterated substrate or NADPH.

Rapid-mixing stopped-flow kinetic analysis

Experiments were performed using a SX.18 MV stopped-flow spectrophotometer from Applied Photophysics (Leatherhead, UK) with a 50 µL flow cell and an instrument dead time below 2.0 ms under the conditions used. Enzyme and substrate solutions were mixed in equal volumes (55 µL). Reactions were initiated upon mixing in flow cell and progress was monitored at 37 °C by absorbance at 340 nm. In binding studies, the fluorescence was

measured. The excitation wavelength was 295 nm and a cutoff (≥ 320 nm) emission filter was applied.

Multiple-turnover reactions. Reaction conditions (after mixing in flow cell): [GFS] (subunit) = 5.0 μM (wild-type), 10 μM (H186K) or 20 μM (C116S, Y143F); [NADPH] = 300 μM (wild-type, H186K) or 350 μM (C116S, Y143F); [substrate **3**] = 250 μM (wild-type, H186K) or 300 μM (C116S, Y143F). Buffer: 10 mM Tris, 25 mM NaCl, pH 8.0. Solution of enzyme and NADPH was held in one syringe and solution of NADPH and substrate **3** was in the other syringe. The absorbance data was averaged in triplicate measurements, each measurement containing minimum of 3 traces with 4000 data points per trace.

Single-turnover reactions. Reaction condition: [GFS] (subunit) = 60 μM; [NADPH] = 60 μM (or [4*S*-^{2}H]-NADPH); [substrate **3**] = 4.0 μM (or deuterated analogues). Buffer: 10 mM Tris, 25 mM NaCl, pH 8.0. Solution of enzyme and NADPH was held in one syringe and solution of substrate **3** was in the other syringe. The absorbance data was averaged in triplicate measurements, each measurement containing minimum of 3 traces with 4000 data points per trace.

Transient kinetic analysis of binding of product **1a**. The time dependence of enzyme intrinsic fluorescence change upon binding of **1a** was recorded. Reaction condition: [GFS] (subunit) = 2.0 μM; [$NADPH_4$] = 15.0 μM; [product **1a**] = 0.0–50 μM. Buffer: 10 mM Tris, 25 mM NaCl, pH 8.0. Solution of enzyme and $NADPH_4$ was held in one syringe and solution of product **1a** was in the other syringe. Note: stopped-flow analysis of the GFS/$NADP^+$ complex upon mixing with product **1a** did not produce a measurable kinetic response in intrinsic fluorescence. The fluorescence data was averaged in triplicate measurements, each measurement containing minimum of 6 traces with 200 data points per trace.

Analysis of multiple- and single-turnover kinetics

The multiple-turnover progress curves were initially analyzed by a linear equation (Eq. S2) or single exponential burst equation (Eq. S3):

$$[\mathrm{NADPH}]_t = [\mathrm{NADPH}]_0 + [\mathrm{E}]k_{ss}t \quad \text{S2}$$

$$[\mathrm{NADPH}]_t = [\mathrm{NADPH}]_{burst}e^{-k_{obs}t} - [\mathrm{E}]k_{ss}t + \mathrm{Y}_0 \quad \text{S3}$$

where $[\mathrm{NADPH}]_t$ is the time-dependent concentration of NADPH, $[\mathrm{NADPH}]_{burst}$ is NADPH consumed in the burst phase, k_{obs} (s^{-1}) is the observed rate constant for the burst phase, [E] is the total enzyme concentration, k_{ss} (s^{-1}) is the steady-state rate constant, $[\mathrm{NADPH}]_0$ is initial NADPH concentration and Y_0 corresponds to $[\mathrm{NADPH}]_0$ minus $[\mathrm{NADPH}]_{burst}$. t is time.

The single-turnover curves were fitted to a single-exponential equation (Eq. S4):

$$[\mathrm{NADPH}]_t = [\mathrm{NADPH}]_\infty + \mathrm{A}_0e^{-k_{st}t} \quad \text{S4}$$

where $[\mathrm{NADPH}]_\infty$ is the NADPH concentration at time approaching infinity, A_0 amplitude equivalent to the substrate concentration used, and k_{st} (s^{-1}) is the single-turnover rate constant.

The curves exhibiting biphasic burst behavior required the use of a double-exponential burst equation (Eq. S5):

$$[\mathrm{NADPH}]_t = [\mathrm{NADPH}]_{burst1}e^{-k_{obs1}t} + [\mathrm{NADPH}]_{burst2}e^{-k_{obs2}t} - [\mathrm{E}]k_{ss}t + \mathrm{Y}_0 \quad \text{S5}$$

where $[\mathrm{NADPH}]_{burst1}$ and $[\mathrm{NADPH}]_{burst2}$ are the amplitudes of the two burst phases, and k_{obs1} and k_{obs2} are their respective observed rate constants (s^{-1}). The results of the burst equations fitting can be found in **Figure S6** and **Table S3**. Reported errors for each obtained value are standard deviations from three separate experiments.

Spectrofluorometric analysis of ligand binding

Fluorescence titrations of wild-type GFS in the binding of coenzyme, substrate and/or product were performed using a fluorescence spectrophotometer F-4500 (Hitachi, Ltd., Tokyo, Japan). Emission spectra were recorded in the range 300–500 nm at 1200 nm/min with the excitation wavelength at 295 nm. The assays (500 µL) were performed in 10 mM Tris buffer (containing 25 mM NaCl, pH 8.0) without agitation (25 °C). The equilibrium dissociation constant (K_d) was determined by following the change in the intrinsic fluorescence ($W_{em} = 335$ nm) of 0.40 µM ligand-free enzyme (or 0.60 µM in the presence of saturating 20.0 µM $NADPH_4$ or 200 µM $NADP^+$), determined at varying concentration of substrate **3**, product **1a**, $NADP^+$ or $NADPH_4$ in the range 0.1–90.0 µM (**Figure S8, S9**). The resulting fluorescence signals were plotted versus ligands concentrations and the data were fitted to a hyperbolic equation (Eq. S6) to obtain the K_d value (**Figure S8, S9**; **Table S3**):

$$\mathrm{F} = \mathrm{F_1} - \frac{\mathrm{F_1} - \mathrm{F_2}}{2[\mathrm{E}]}\left(K_\mathrm{d} + [\mathrm{L}] + [\mathrm{E}] - \sqrt{(K_\mathrm{d} + [\mathrm{L}] + [\mathrm{E}])^2 - 4[\mathrm{E}][\mathrm{L}]}\right) \qquad \text{S6}$$

where F is the fluorescence emission, [L] is concentration of ligand, F_1 is the initial and F_2 is the final fluorescence signal, [E] is the enzyme subunit concentration.

It was confirmed that the unbound ligands, in the concentrations used, did not interfere with the fluorescence measurements.

Kinetic simulation and parameter fitting

To refine the mechanistic interpretations and account for an unusual burst behavior in multiple-turnover experiments, global simulation-fitting of kinetic data based on numerical integration of rate equations was performed using COPASI.[6] The family of curves resulting from the pre-steady-state multiple- and single-turnover experiments were fit simultaneously to the proposed mechanism. The kinetic models for wild-type enzyme (**Figure 3a** and **3b**) were developed

based on the evidence from KIE, structural and transient kinetic studies, assuming random binding of substrates as well as random product release. Additionally, the product release steps were assumed to be irreversible due to the initial absence of product, and substrate binding was assumed to be in rapid equilibrium under the experimental conditions used. During the initial simulation, the rate constants describing chemistry and product release (k_1, k_{-1}, k_2, k_3, k_4, k_5) were allowed to float within a reasonably confined range (between 0.01 and 100 s^{-1}) to avoid physically meaningless results. Additionally, the constraint for k_{ss} was imposed by incorporating the net rate expressions[12] derived from the proposed mechanisms a (Eq. S7–S9) and b (Eq. S7, S8 and S10). This approach ensured consistency between pre-steady-state and steady-state data while reducing parameter redundancy. A constrained range for k_{ss} of 2.0 to 2.4 s^{-1} was applied, based on k_{cat} value from steady-state experiment with the wild-type enzyme. Standard errors for the fitted rate constants were estimated as standard deviations based on the variability observed during global fitting. The goodness of fit to the model was assessed by confidence contour analysis, in which all parameters, except for k_{-1}, were well constrained by the data. The k_{-1} rate constant was not identifiable from the data and was instead empirically bounded through parameter sensitivity analysis. Its upper limit was defined as the highest value that did not introduce significant perturbation to the fit quality or to the estimates of other rate constants. Obtained rate constants can be found in **Figure 3** and **Table S4**.

$$k_{ss} = \frac{k'_1 k'_{2,3}}{k'_1 + k'_{2,3}} \qquad \text{S7}$$

$$k'_1 = \frac{k_1(k_2+k_3)}{k_{-1}+k_2+k_3} \qquad \text{S8}$$

$$k'_{2,3}{}^{a} = \frac{k_2+k_3}{1+\frac{k_2}{k_4}+\frac{k_3}{k_5}} \qquad \text{S9}$$

$$k'_{2,3}{}^{b} = \frac{k_2+k_3}{1+\frac{k_2}{k'_2[3]}+\frac{k_2}{k_4}+\frac{k_3}{k'_3[\mathrm{NADPH}]}+\frac{k_3}{k_5}} \qquad \text{S10}$$

Kinetic simulation for H186K variant was performed according to **Scheme S2**. Similar to the wild-type enzyme, a constrained range for k_{ss} of 0.1 to 0.6 s^{-1} was applied, based on the k_{cat} (= k_{ss}) value from steady-state experiment. The parameters k_{obs}, $[NADPH]_{burst}$ and k_{ss} in this case were derived from Eq. S3 according to net rate constants from minimal mechanism in **Scheme S2** (Eq. S11–S13):

$$k_{obs} = k_1 + k_{-1} + k_P \qquad \text{S11}$$

$$[NADPH]_{burst} = [E]_t \frac{k_1(k_1 + k_{-1})}{(k_1+k_{-1}+k_P)^2} \qquad \text{S12}$$

$$k_{ss} = \frac{k_1 k_P}{k_1+k_{-1}+k_P} \qquad \text{S13}$$

The obtained rate constants can be found in **Table S5**.

Analysis and simulation of transient binding kinetics of GDP-L-fucose

The stopped-flow progress curves of fluorescence change on ligand binding were initially analyzed using single exponential equation (Eq. S14):

$$F_t = F_\infty + Ae^{-k_{obs}t} \qquad \text{S14}$$

where F_t is the time-dependent fluorescence signal, A is the amplitude of fluorescence change, F_∞ is the fluorescence signal at time approaching infinity and k_{obs} (s^{-1}) is the observed rate constant.

Resulting k_{obs} values showed a hyperbolic dependence on the concentration of GDP-L-fucose (**1a**), consistent with a two-step binding mechanism (**Figure 5c**, main text). The mathematical relationship between k_{obs} and the ligand concentration for such a two-step mechanism is represented by Equation S15:

$$k_{obs} = k'_{-1} + k'_1 \frac{[L]}{K_d+[L]} \qquad \text{S15}$$

where [L] is the ligand concentration, K_d is the dissociation constant, k'_1 and k'_{-1} are the rate constants for the forward and reverse conformational changes, respectively.

Global simulation-fitting of the kinetic data was performed using COPASI in the way described for the transient absorbance traces. The observed fluorescence signal was calculated as a weighted sum of all relevant enzyme species, with response coefficients assigned to account for differences in intrinsic fluorescence. A two-step model fitting (**Figure 5c**) was done globally across series of ligand concentrations, and parameter identifiability was assessed through confidence contour analysis.

Quantification of enzyme-bound NADPH and $NADP^+$ at steady state of reaction

The reaction was performed in 10 mM Tris buffer (containing 25 mM NaCl, pH 8.0) in 10 mL volume with agitation (37 °C, 300 rpm). The reaction mixture contained 1.0 mM of substrate **3**, 1.2 mM of NADPH and 0.7 µM of wild-type enzyme. Reaction was incubated for 3 min, after which 12 mL of iced-cold buffer (10 mM Tris/HCl, 25 mM NaCl, pH 1.3) was added to decrease the pH to 4.0 and stop enzyme activity, while retaining the enzyme in a soluble state. The reaction mixture was immediately transferred into a Vivaspin Turbo 15 (30000 MWCO; Sartorius AG) and centrifuged at 0 °C (2880 × g) until 0.8 mL of the original volume remained. The enzyme was then washed twice with 4 mL of ice-cold buffer (10 mM Tris/HCl, 25 mM NaCl, pH 4.0). The flow-through from the reaction mixture and both wash fractions were collected and analyzed by HPLC to confirm that the substrate/product ratio remained unaffected by washing procedure. The enzyme was subsequently concentrated to 35 µM in 100 µL, then denatured with 100 µL of MeOH. NADPH and $NADP^+$ released from GFS were detected by HPLC (**Figure S7**). A control experiment was conducted in the absence of enzyme to ensure the stability of substrates during experiment.

Analytical methods

HPLC. The sugar nucleotides and NADPH/$NADP^+$ were separated using Shimadzu (Korneuburg, Austria) Prominence HPLC-UV system with detection at 258 nm. NADPH and $NADP^+$ were separated using Kinetex C18 (5 μm, 100 Å, 50 × 4.6 mm; Phenomenex, Aschaffenburg, Germany). Compounds were eluted by applying an isocratic flow (2 mL/min) at 40 °C with a mobile phase composed of 92% of buffer A (20 mM potassium phosphate buffer, 40 mM tetrabutylammonium bromide, pH 5.9) and 8% of organic solvent B (acetonitrile). GDP-D-mannose and substrate **3** were separated using Kinetex C18 (5 μm, 100 Å, 150 × 4.6 mm; Phenomenex) column. Compounds were eluted applying an isocratic flow (1 mL/min) at 40 °C with a mobile phase composed of 100% of buffer A. For the separation of compounds in steady-state enzyme-bound experiment, gradient method with YMC-Triart C18 (5 μm, 120 Å, 150 × 4.6 mm; YMC Europe GmbH, Dinslaken, Germany) column was used. The gradient method consisted of next steps: 0–12 min 25 % B; 12–20 min 50 % B; 20–25 min 25 % B. The amount of bound NADPH and $NADP^+$ was determined based on the calibration curves shown. Defined standard solutions (15–200 μM) were prepared in doubly distilled H_2O and were directly used for HPLC analysis.

NMR. The purity and identity of the synthesized substrates were determined by ^1H NMR. NMR measurements were carried out on a Varian INOVA 500 MHz spectrometer (Agilent Technologies, Santa Clara, CA, USA) at 30 °C using VNMRJ 2.2D software. ^1H NMR spectra was measured at 499.98 MHz with a 5–mm indirect detection PFG probe (relaxation delay 1 s; 90° proton pulse; acquisition time 2.048 s; spectral width 8 kHz; number of points 32000). The spectra were analyzed using MestReNova 16.0 (Mestrelab Research, Santiago de Compostela, Spain). Probes for measurements were prepared in 50 mM potassium phosphate

buffer (p^2H 8.0) in a total volume of 750 µL, where sugar nucleotides were dissolved to 10 mM.

Crystallization and data collection

Purified wild-type enzyme was crystallized using the sitting drop vapor diffusion method. For the GFS complex with $NADP^+$, co-crystallization was performed using 12 mg/mL of the enzyme and 1.0 mM $NADP^+$. For the GFS complex with $NADP^+$/GDP, the same enzyme concentration was used, with 1.0 mM $NADP^+$ and 5.0 mM GDP. Sitting drops were set up in 96-well 3-drop SWISSCI crystallization plates (Molecular dimensions, Newmarket, Suffolk, UK). The drop volume was 150 nL and consisted of 50 nL, 75 nL or 100 nL protein solution mixed with 100 nL, 75 nL or 50 nL of precipitant solution, respectively. Crystallization drops were equilibrated against a reservoir containing 20 µL of precipitant solution. Crystal hits were detected in various conditions at 25 °C. Crystallization conditions were optimized and suitable conditions for different combinations of enzyme and ligands were as follows: GFS with $NADP^+$, 20% PEG3350, 0.1 M ammonium citrate dibasic (pH 5.5); GFS with $NADP^+$ and GDP, 20% PEG3350, 0.3 NaBr, 0.1 M Bis-Tris propane (pH 7.5), 10% ethylene glycol. Prior to data collection, crystals were briefly transferred to a solution consisting of 20% ethylene glycol added to the corresponding precipitate and flash frozen in liquid nitrogen. Data were collected at the Diamond light source beamlines I03 or X10SA using a Pilatus 6 M detector (Dectris, Baden, Switzerland). Data were integrated with XDS[7] and scaled with AIMLESS[8], as part of the XIA2[9] auto-processing. Data collection statistics are summarized in **Table S6**.

The structure of GFS was determined by molecular replacement with PHASER[10] using PDB ID 1GFS (the GFS from *Escherichia coli*) as a search model. Model building was performed with COOT[11] and refinement was carried out with phenix.refine[12]. Ligand restraints for $NADP^+$ (Ligand ID: NAP) and GDP (Ligand ID: GDP) were generated with the Grade Web Server and

the molecules were initially placed using phenix.ligand_fit[12]. Final structures were evaluated using MolProbity[13]. The numbers of Ramachandran outliers are <0.2%. Figures of the structural models were prepared using ChimeraX[14]. Two-dimensional diagrams of the binding sites were prepared using LigPlot[15] software. Refinement statistics of the reported structures are shown in **Table S6**.

SUPPORTING FIGURES

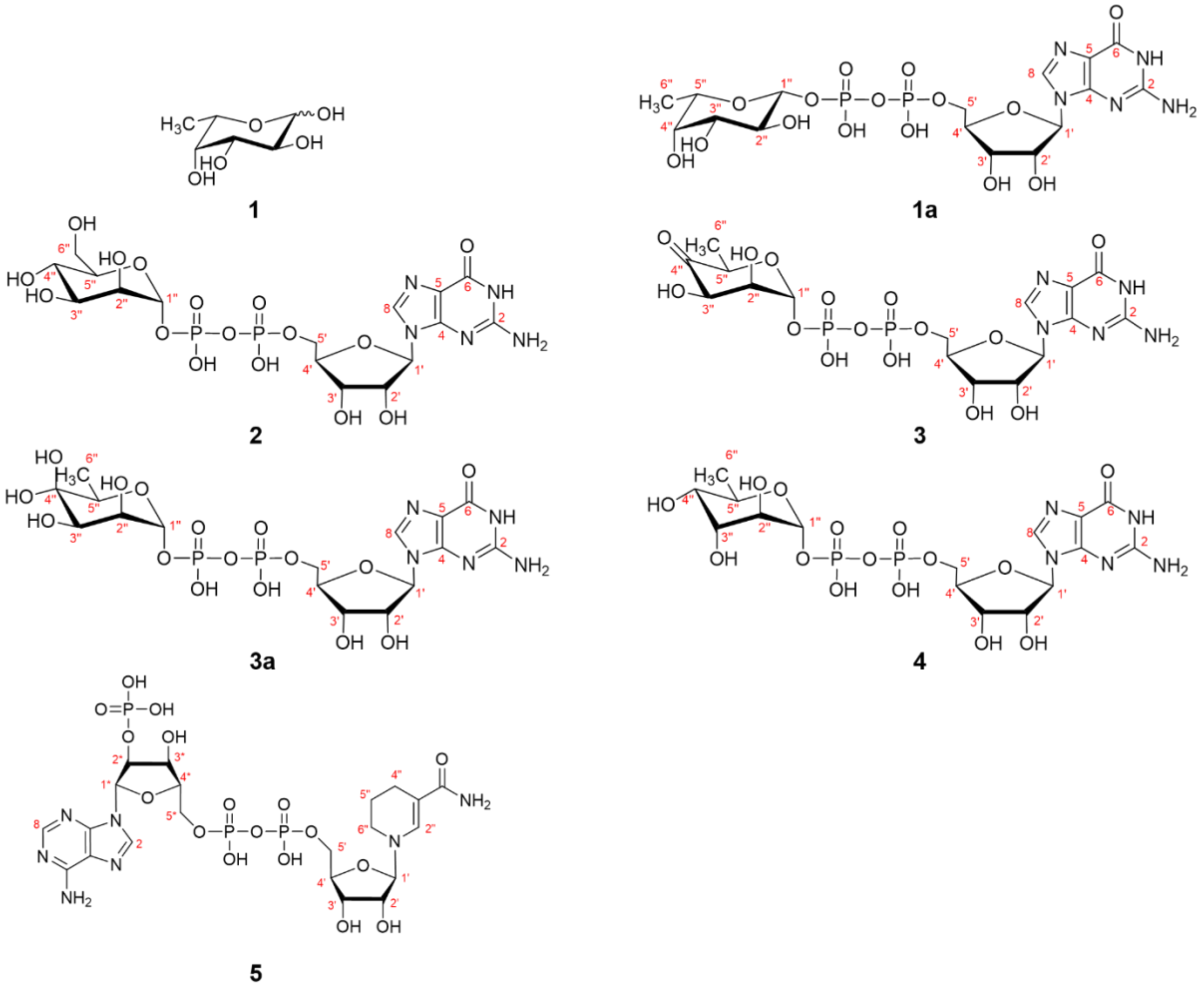

Figure S1. Structure of compounds described in this work. **1**: L-fucose, **1a**: GDP-L-fucose, **2**: GDP-D-mannose, **3**: GDP-4″-keto-6″-deoxy-D-mannose, **3a**: GDP-4″-diol-6″-deoxy-D-mannose, **4**: GDP-6″-deoxy-D-altrose, **5**: 1″,4″,5″,6″-tetrahydro-NADPH.

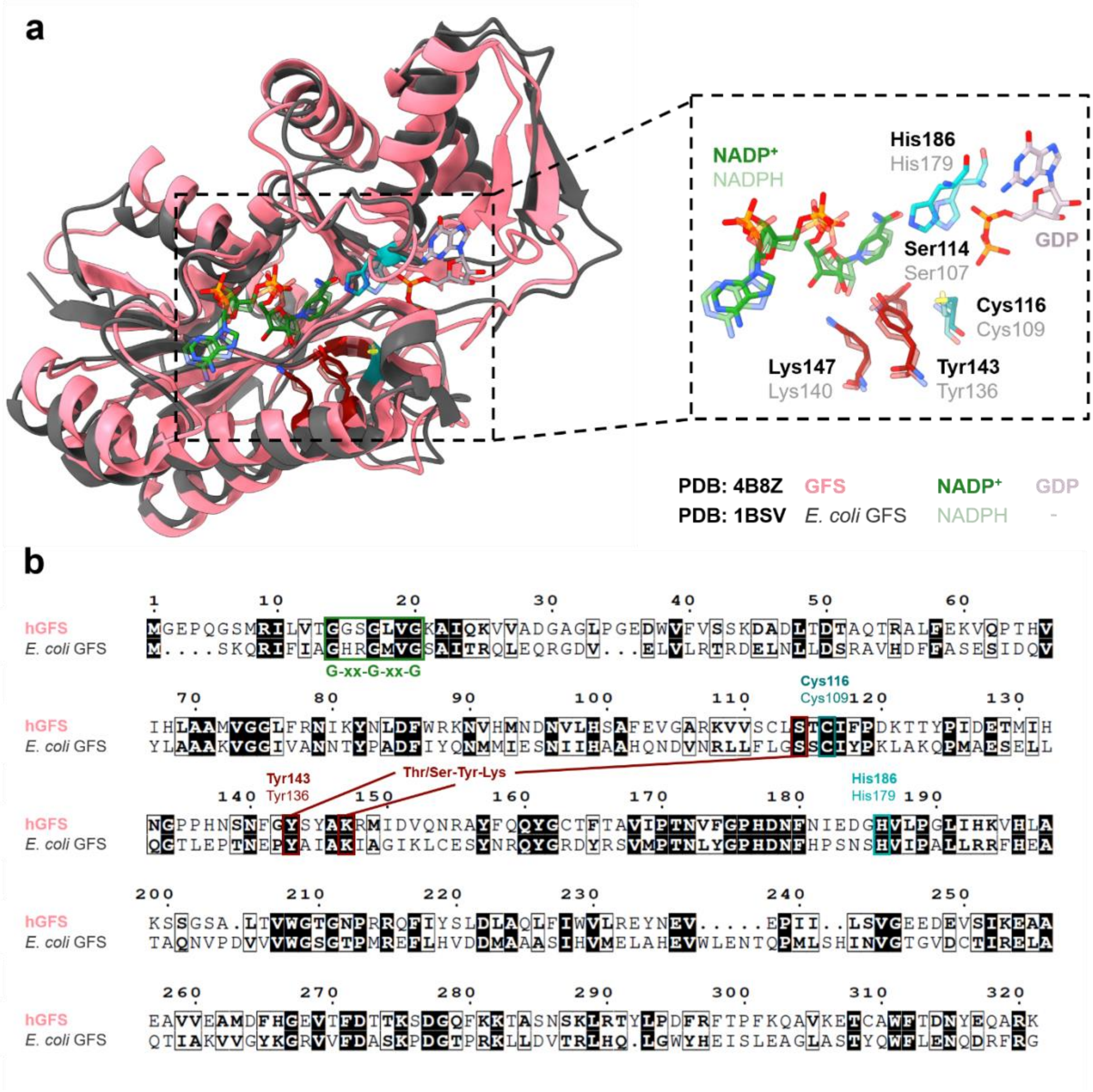

Figure S2. Structure and sequence comparison of the human and *E. coli* GFS. (**a**) Superimposed structures (RMSD = 2.557 Å) of the human GFS complex with NADP$^+$/GDP (4B8Z; pink), and the *E. coli* GFS complex with NADPH (1BSV; grey). A zoom into the active site is shown, with NADP$^+$/NADPH (dark green), GDP (light purple) and catalytic residues indicated. Residues and ligand from *E. coli* GFS are shown with 60% transparency. (**b**) Sequence alignment between human and *E. coli* GFS sequences. Conserved residues, including the epimerization dyad and the SDR triad as well as the Gly-rich motif responsible for coenzyme binding, are highlighted. Sequences were processed using the ClustalW[16] and ESPript 3[17] online tools.

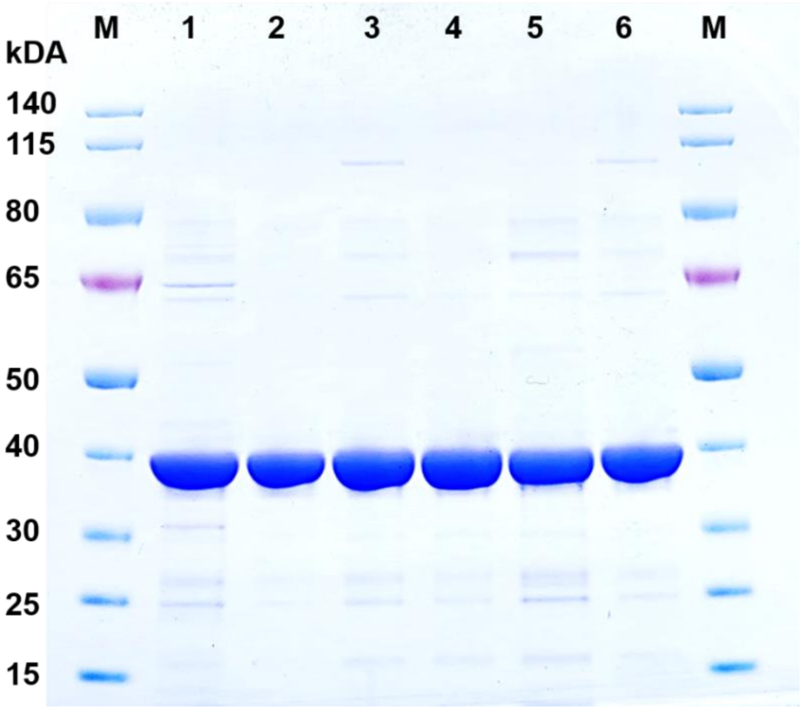

Figure S3. SDS polyacrylamide gel showing the purified preparations of GFS enzymes. Lanes 1: wild-type, 2: C116S, 3: H186K, 4: Y143F, 5: C116A, 6: H186A, M: PageRuler™ Prestained Protein Ladder.

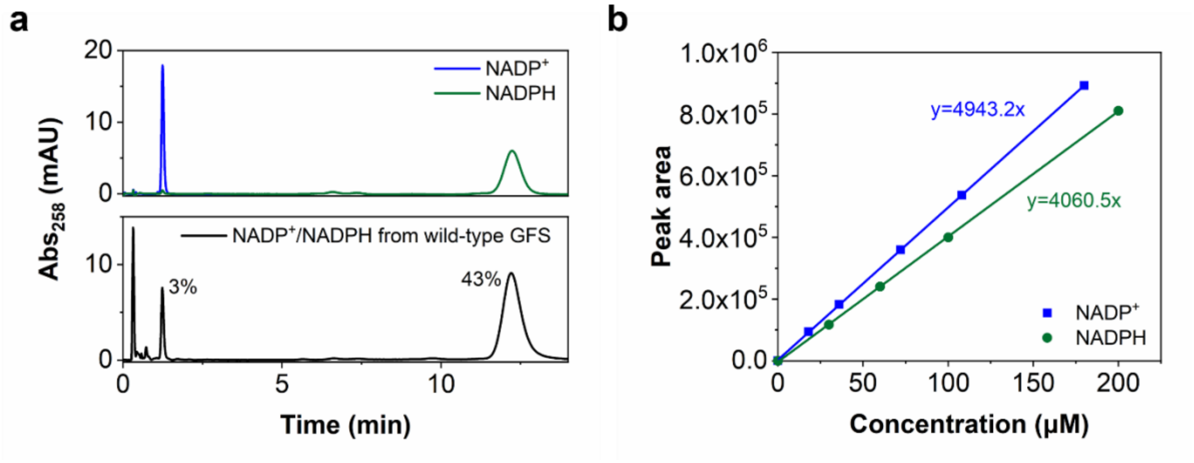

Figure S4. Quantification of enzyme-bound NADPH and $NADP^+$ from the as-isolated wild-type GFS. (**a**) HPLC chromatograms of extracted NADPH and $NADP^+$ from 220 µM of the enzyme with authentic standards. (**b**) The amount of bound NADPH and $NADP^+$ was determined based on the linear calibration shown. Defined standard solutions (15–200 µM) were directly used for HPLC analysis at 258 nm.

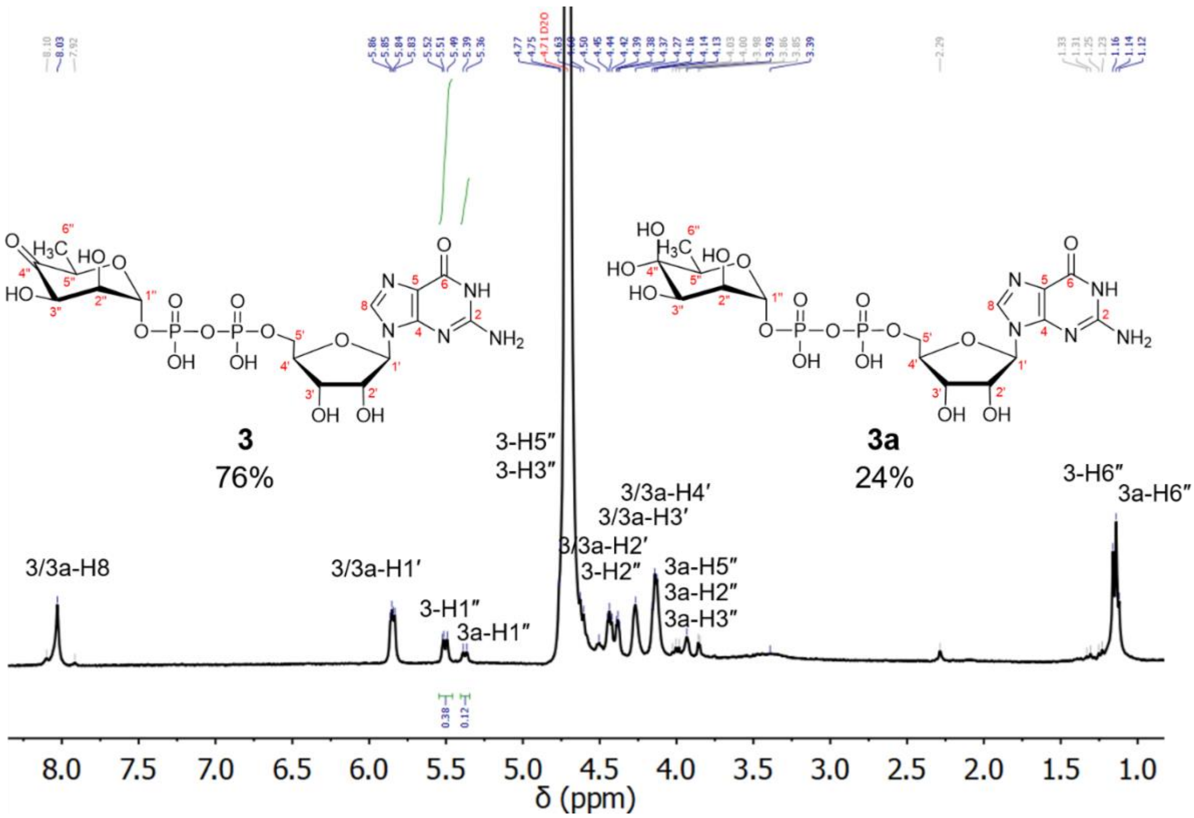

Figure S5. Full ^{1}H NMR spectrum of GDP-4″-keto-6″-deoxy-D-mannose (**3**), containing also GDP-4″-diol-6″deoxy-D-mannose (**3a**). Indicated ratio of keto- and diol-forms based on integrated anomeric peaks at 5.51 and 5.38 ppm, respectively. (^2H_2O, 500 MHz): δ = 8.03 (s, 1 H, H8), 5.85 (dd, J = 6.3 Hz, 2 H, H1′), 5.51 (dd, J = 7.0 Hz, 1 H, 3-H1″), 5.38 (dd, J = 7.0 Hz, 1 H, 3a-H1″), 4.62 (d, J = 6.4 Hz, 1 H, 3-H5″), 4.44 (t, J = 4.4 Hz, 1 H, H2′), 4.39 (t, J = 3.3 Hz, 1 H, 3-H2″), 4.27 (br. t, 3 H, H3′), 4.09 (m, 1 H, H4′), 4.04 (dd, 1 H, 3a-H5″), 3.94 (br. s, 1 H, 3a-H2″), 3.86 (d, 1 H, 3a-H3″), 1.15 (dd, 3 H, 3-H6″), 1.13 ppm (dd, 3 H, 3a-H6″).

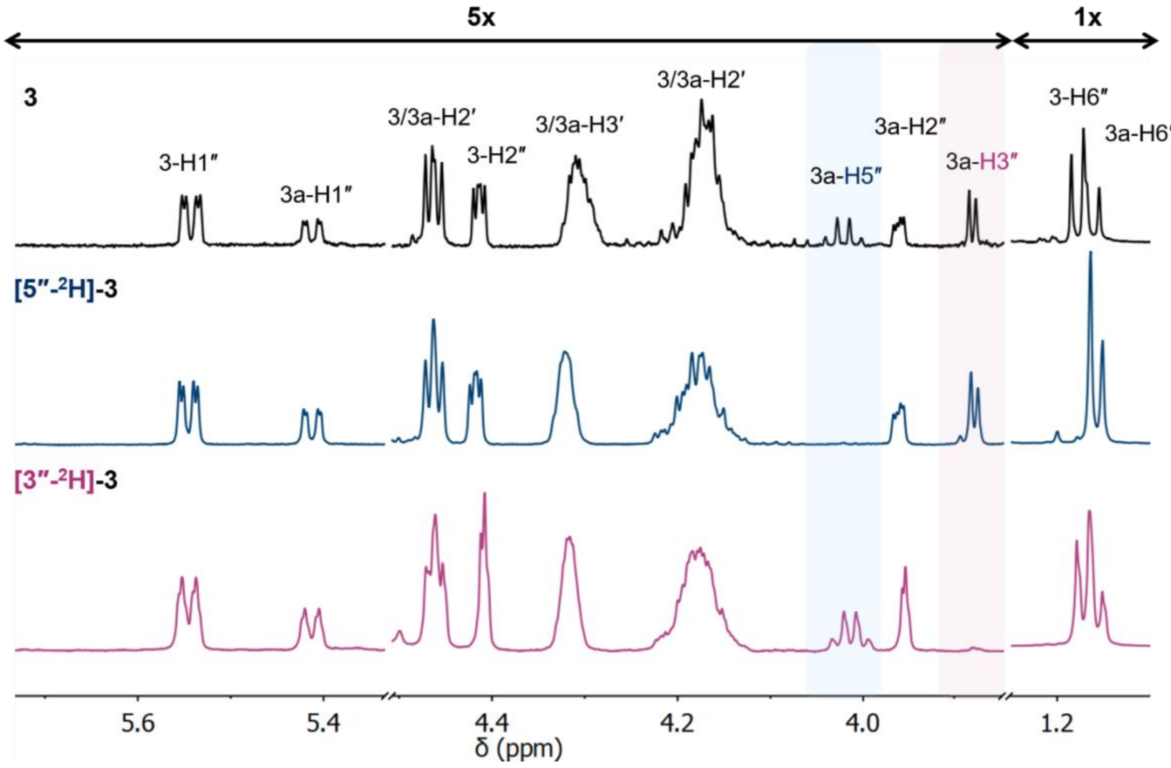

Figure S6. ^{1}H NMR spectra of the synthesized deuterated substrates. Incorporation of deuterium label for [3″-^{2}H]-**3** (shaded pink area) and [5″-^{2}H]-**3** (shaded blue area). Only signals from the diol form are visible; (^2H_2O, 500 MHz): δ = 4.01 (dd, 1 H, 3a-H5″), 3.86 (d, 1 H, 3a-H3″). Signals at the positions C-3″ and C-5″ in the keto form are overlapped by the water peak.

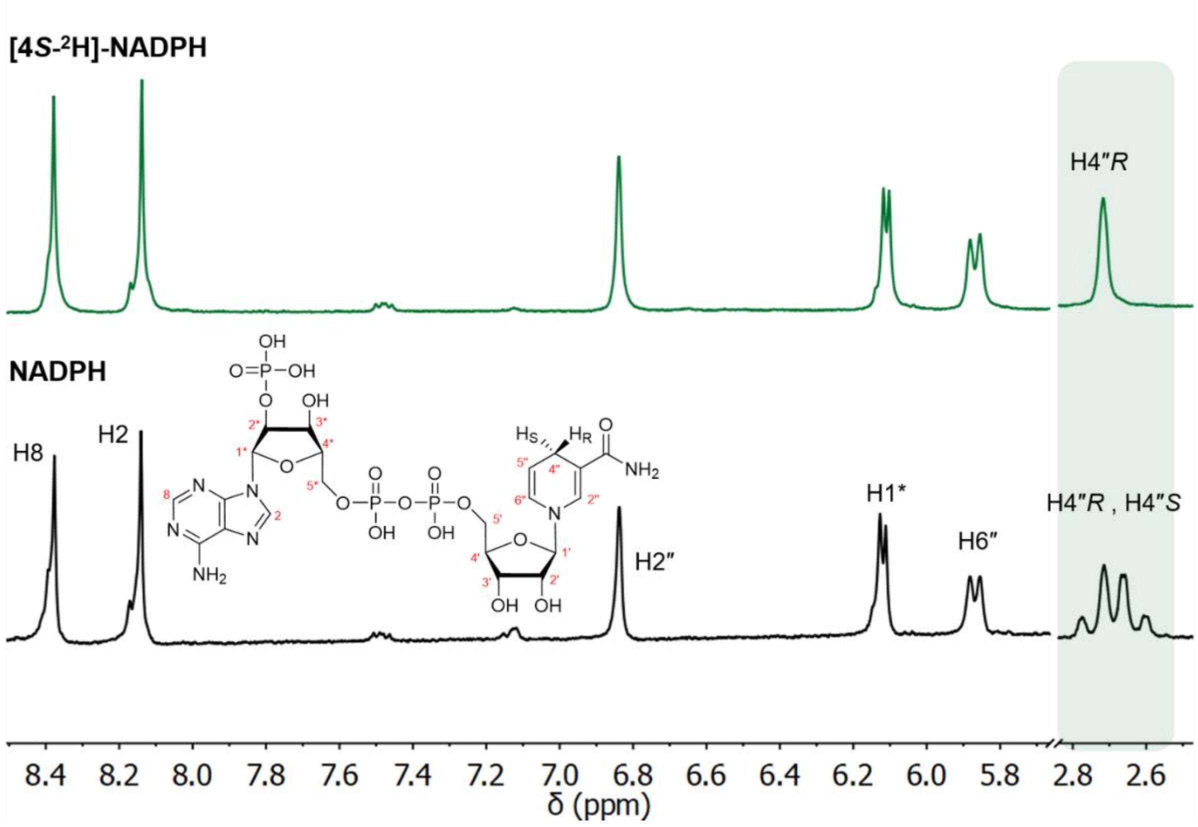

Figure S7. ^{1}H NMR spectrum of synthesized deuterated NADPH. Incorporation of label at 4*S*-position of NADPH highlighted in the shaded green area. (^2H_2O, 500 MHz): δ = 8.35 (s, 1 H, H8), 8.13 (s, 1 H, H2), 6.84 (s, 1 H, H2″), 6.10 (d, 1 H, H1*), 5.89 (dt, 1 H, H6″), 2.7 (q, 2 H, H4″*R*), 2.72 ppm (s, 2 H, H4″*S*).

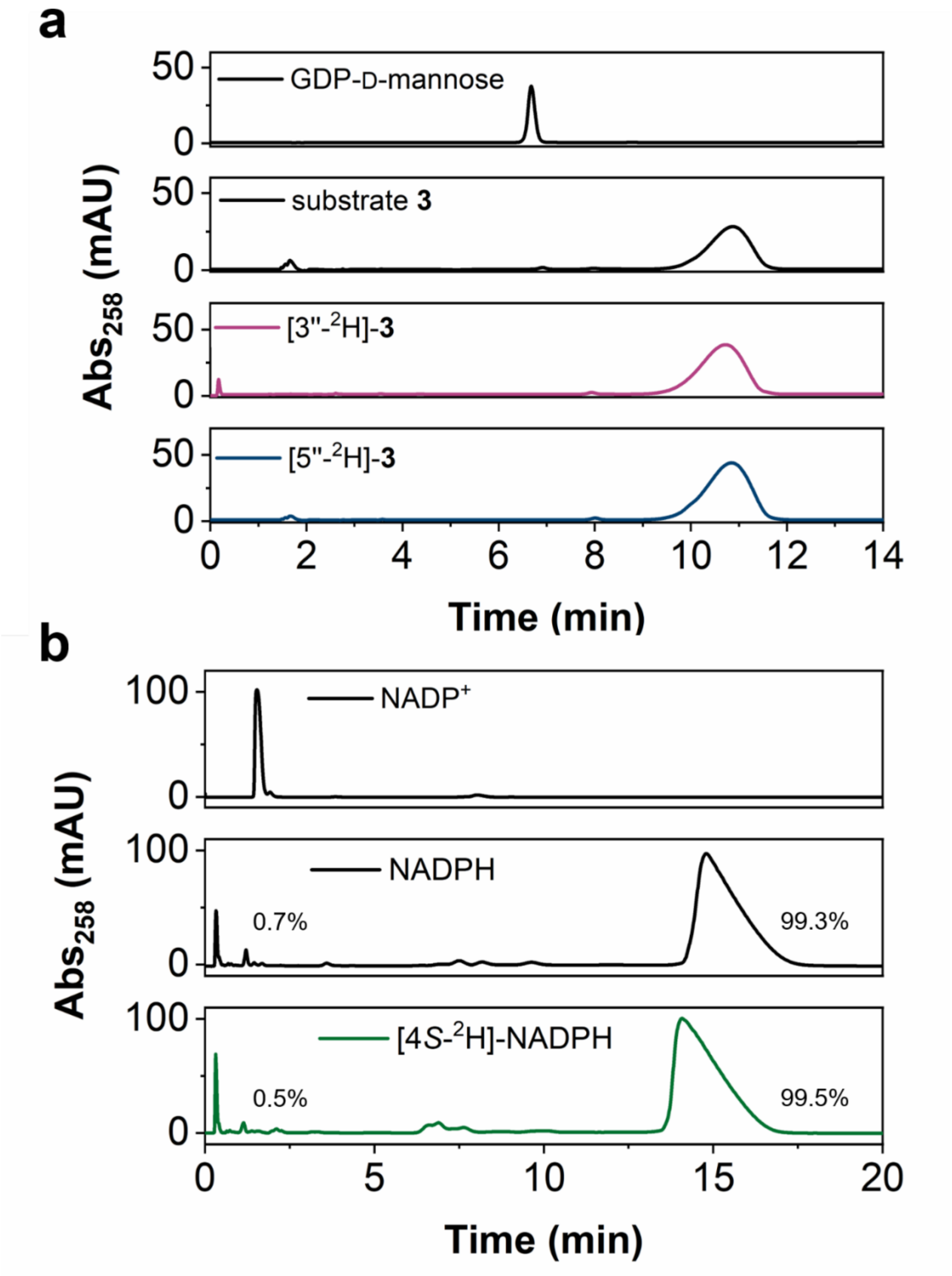

Figure S8. HPLC chromatograms of the synthesized substrates. (**a**) GDP-D-mannose, substrate **3**, [3″-^{2}H]-**3** and [5″-^{2}H]-**3**. (**b**) NADPH and [4*S*-^{2}H]-NADPH with an authentic $NADP^+$ standard to indicate the percentage of $NADP^+$ in the synthesized compounds.

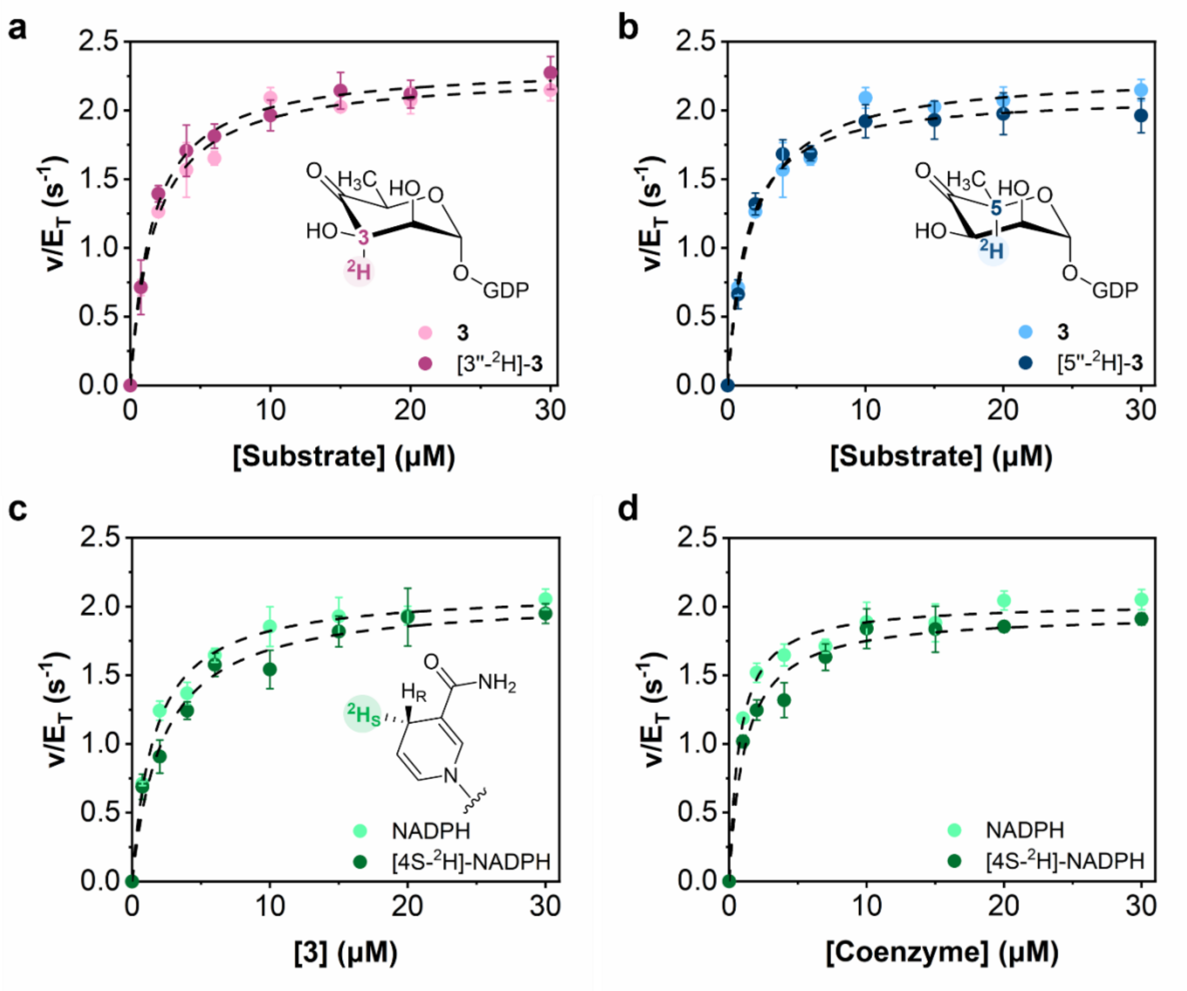

Figure S9. Michaelis-Menten curves of wild-type GFS for conversion of unlabeled and [^{2}H]-labeled substrate **3** or NADPH used for KIE determination. (**a, b**) Varied substrates **3** (light pink or blue), [3″-^{2}H]-**3** (dark pink) and [5″-^{2}H]-**3** (dark blue); [NADPH] = 100 µM. (**c**) Varied substrate **3**; [coenzyme] = 100 µM. (**d**) Varied NADPH (light green) and [4*S*-^{2}H]-NADPH (dark green); [substrate **3**] = 100 µM. Buffer: 10 mM Tris, 25 mM NaCl, pH 8.0 (37 °C). Error bars show the standard deviation of triplicate measurements. Dashed lines represent best global fit to Eq. S1. Coenzyme is either NADPH or [4*S*-^{2}H]-NADPH.

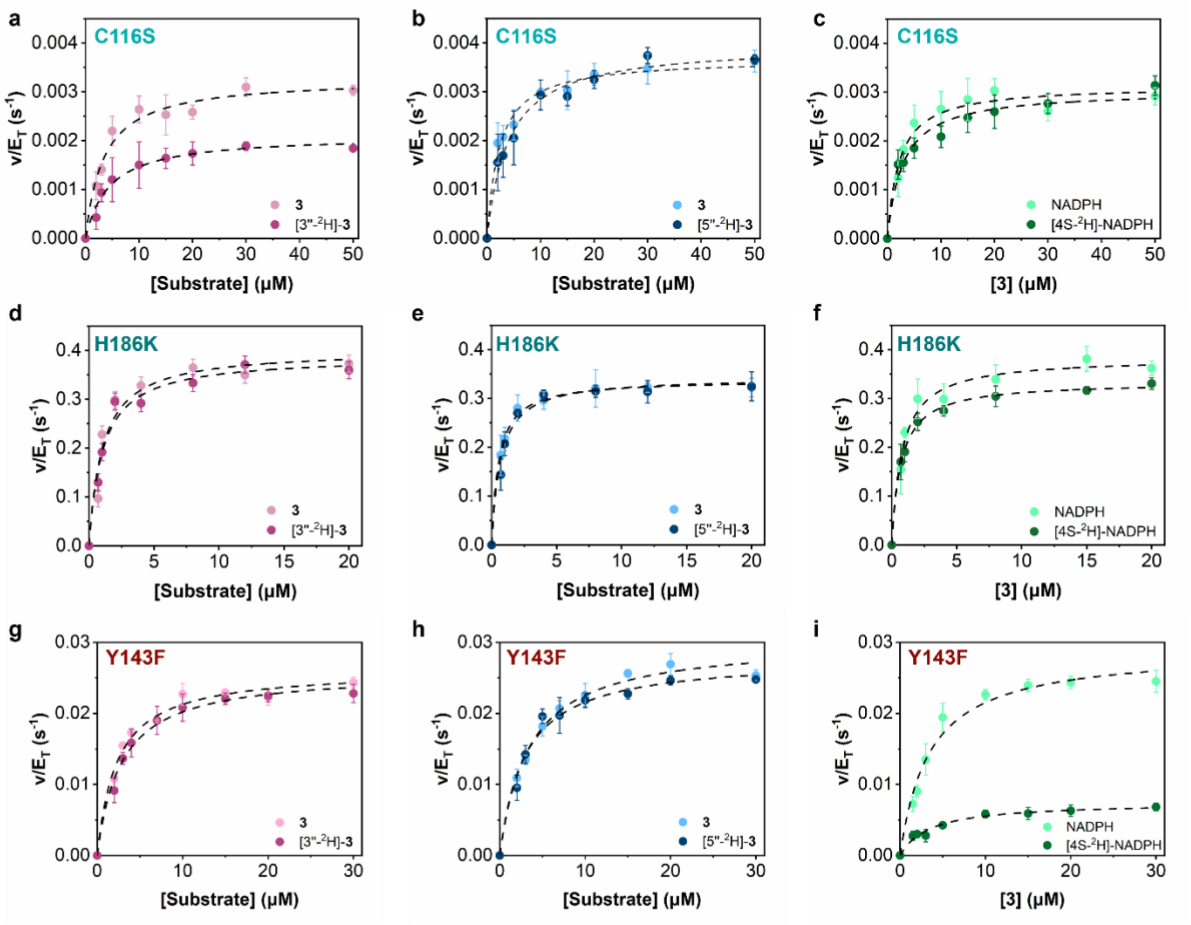

Figure S10. Michaelis-Menten curves of GFS variants for conversion of unlabeled and [^{2}H]-labeled substrate **3** or NADPH used for KIE determination. C116S (**a**–**c**), H186K (**d**–**f**) and Y143F (**g**–**i**). All kinetic curves obtained with varied unlabeled or [^{2}H]-labeled substrates **3**, where NADPH or [4*S*-^{2}H]-NADPH were at saturating concentration of 100 µM. Buffer: 10 mM Tris, 25 mM NaCl, pH 8.0 (37 °C). Error bars show the standard deviation of triplicate measurements. Dashed lines are best global fit to Eq. S1.

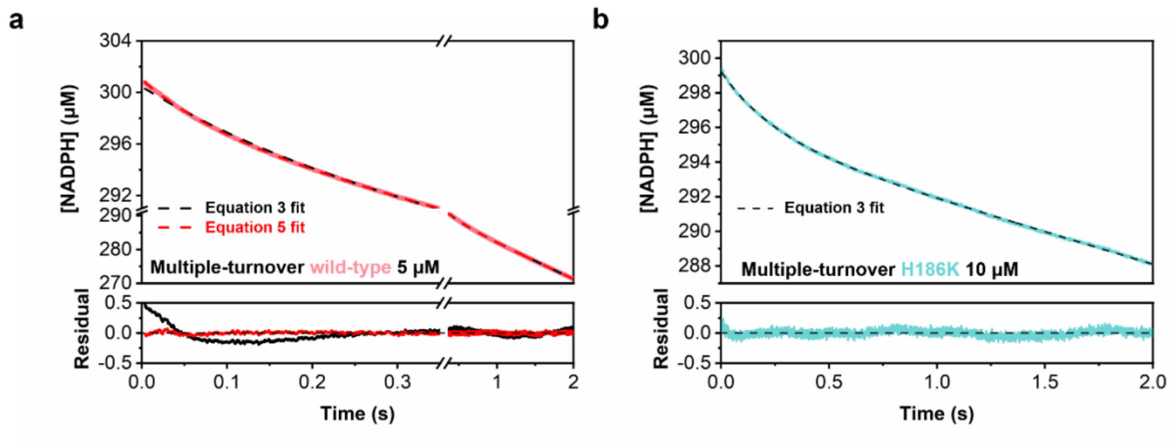

Figure S11. Multiple-turnover progress curves for the wild-type enzyme (**a**) and H186K (**b**) fitted to single- and double-exponential burst equations. The black short dashed line represents the fit to Eq. S3. The red short dashed line represents the fit to Eq. S5 in the case of the wild-type enzyme.

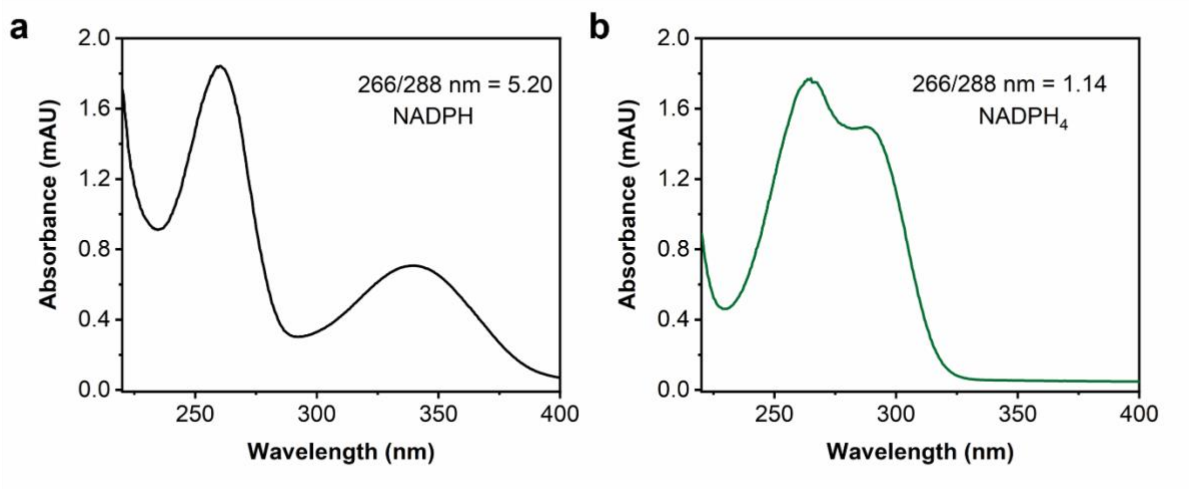

Figure S12. Absorbance spectra for (**a**) NADPH and synthesized analog (**b**) $NADPH_4$, with 266/288 nm ratio indicated in panels.

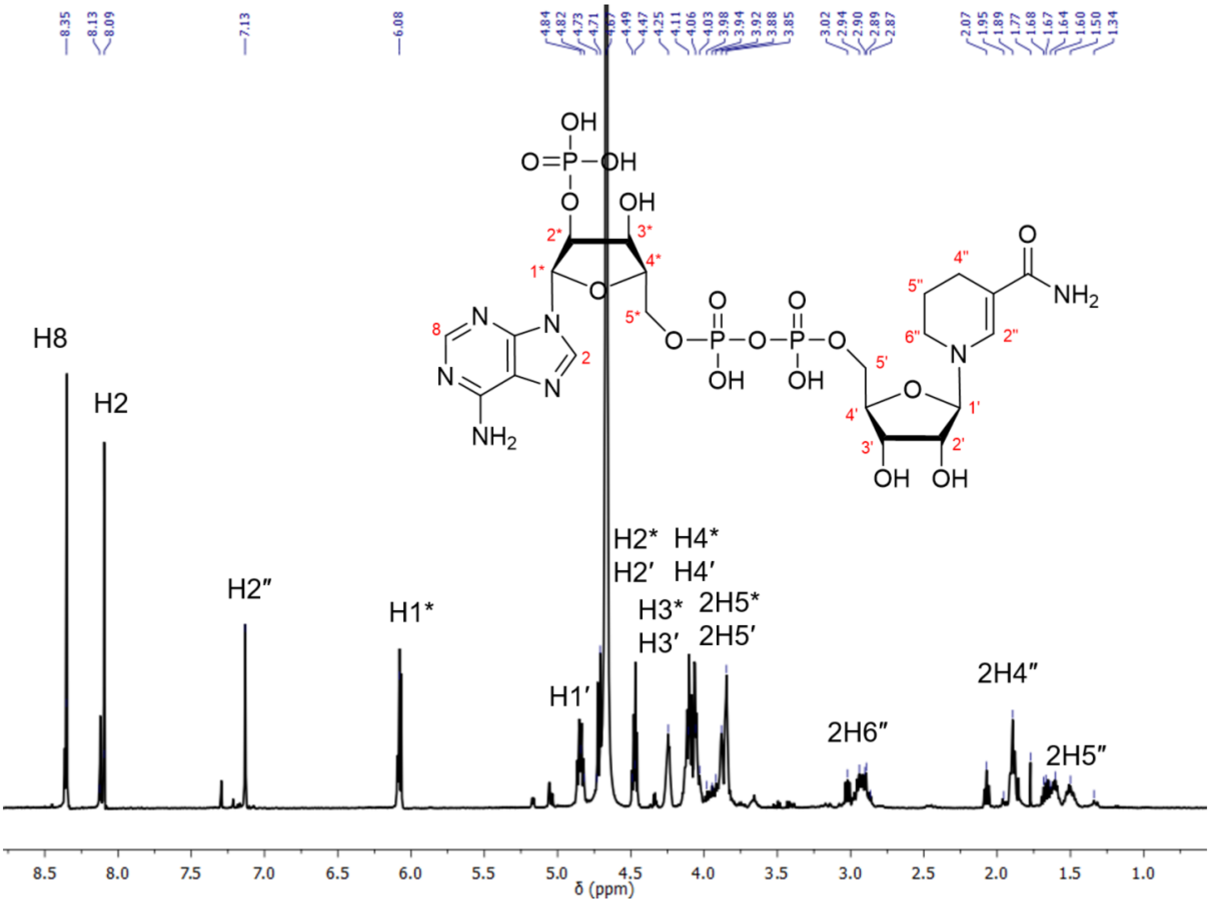

Figure S13. ^{1}H NMR spectrum of the synthesized NADPH4. (^2H_2O, 500 MHz): δ = 8.35 (s, 1 H, H8), 8.09 (s, 1 H, H2), 7.13 (s, 1 H, H2″), 6.08 (d, 1 H, H1*), 4.84 (m, 1H, H1′), 4.49 (m, 2H, H2′-H2*), 4.25 (m, 2H, H3′-H3*), 4.25 (m, 2H, H3′-H3*), 3.85–4.11 (m, 6H; H4′, H4*, 2H5′, 2H5*) 2.87–3.02 (m, 2 H, H6″), 1.8–2.07 (m, 2 H, H4″), 1.48–1.7 ppm (m, 2 H, H5″).

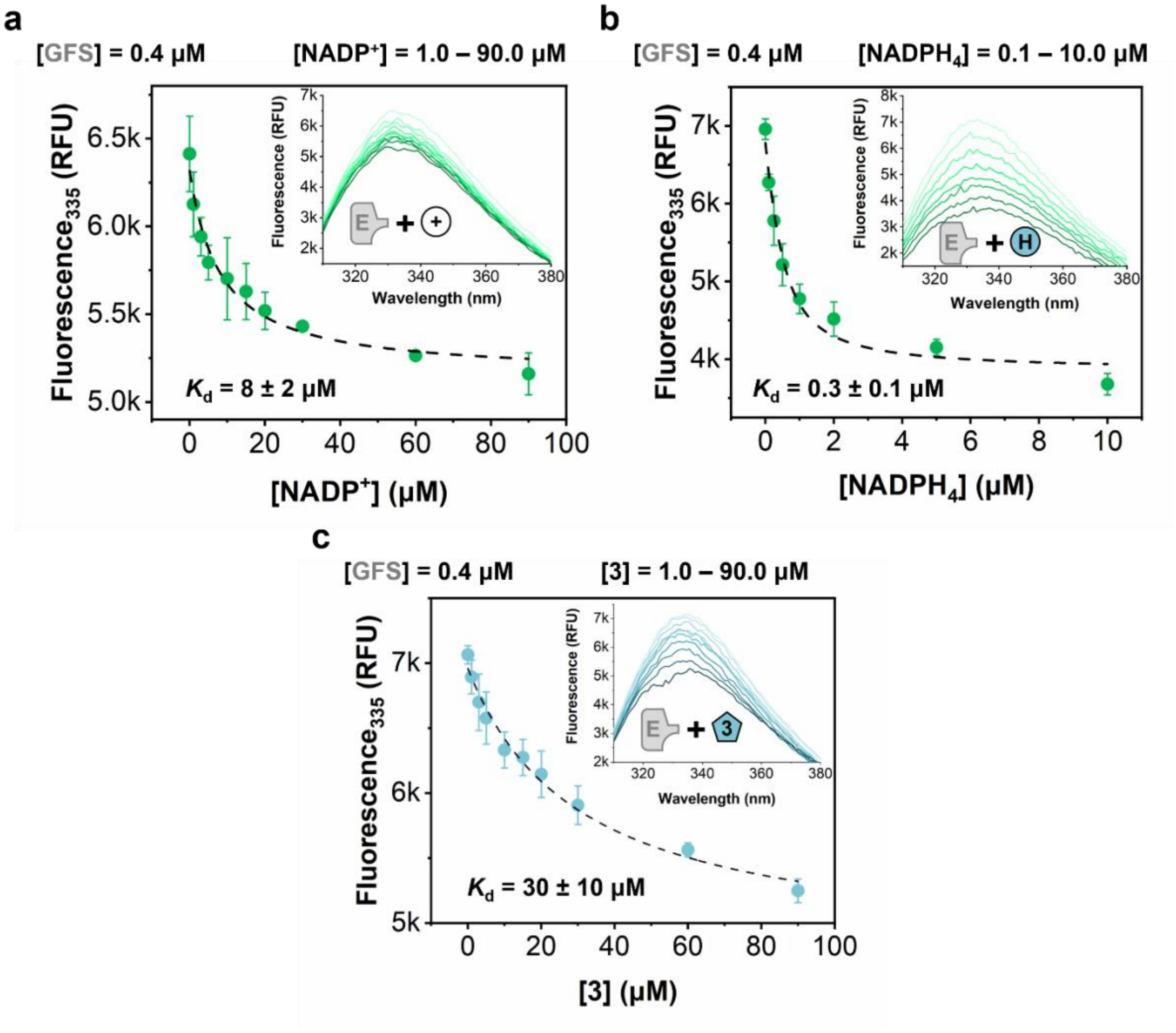

Figure S14. Equilibrium binding analyzed by quenching of intrinsic fluorescence of ligand-free wild-type GFS titrated with $NADP^+$ (**a**) and $NADPH_4$ (**b**) and substrate **3** (**c**) as ligands. All curves were obtained at constant [GFS] (subunit) with varied [$NADP^+$], [$NADPH_4$] or [**3**]. The concentrations are shown in the panels. Buffer: 10 mM Tris, 25 mM NaCl, pH 8.0 (25 °C). Error bars show the standard deviation of triplicate measurements. Dashed lines are best fit to Eq. S2.

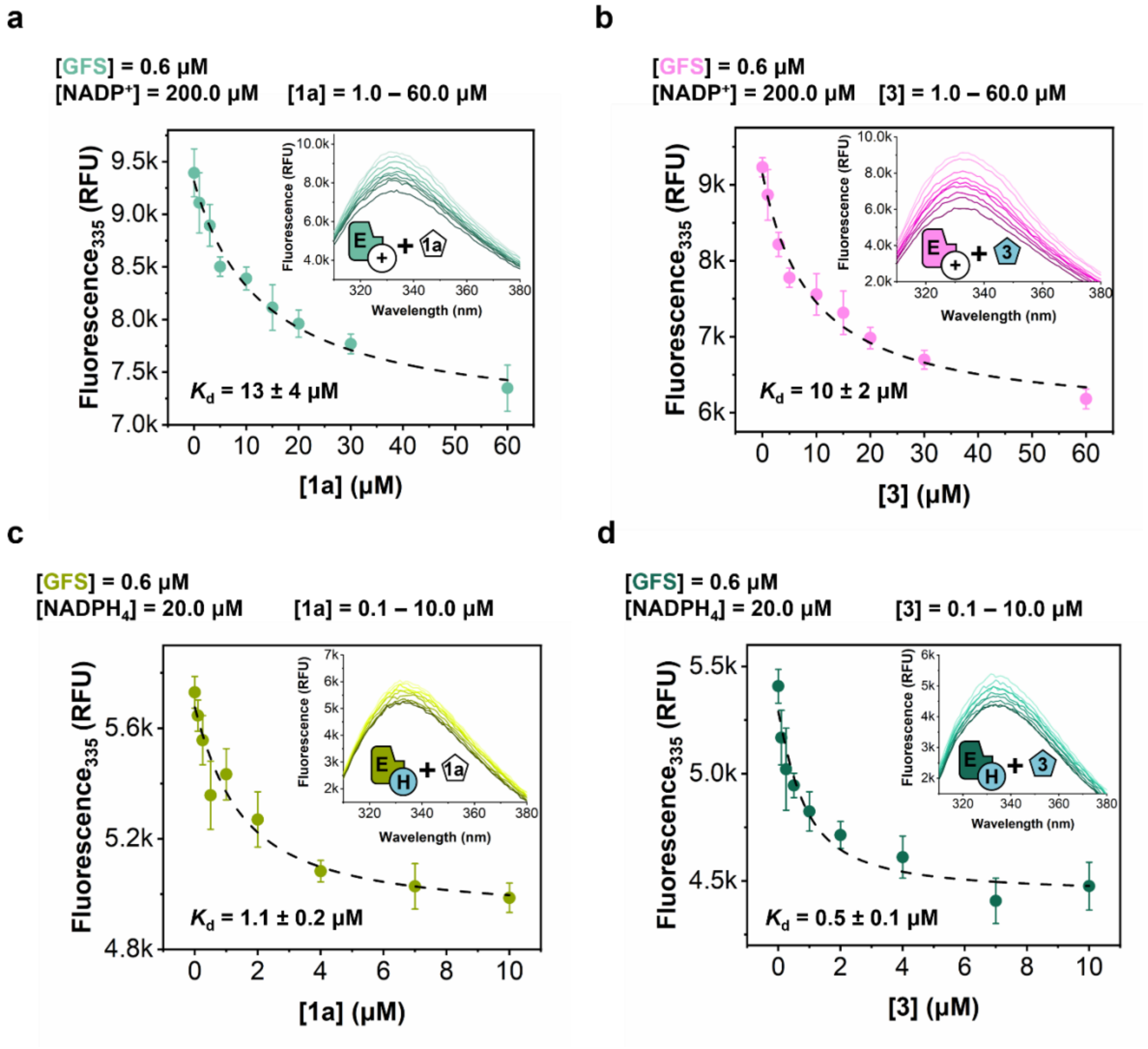

Figure S15. Equilibrium binding analyzed by quenching of intrinsic fluorescence of wild-type GFS in complex with $NADP^+$ or $NADPH_4$. GFS/$NADP^+$ complex titrated with product **1a** (**a**) and substrate **3** (**b**); GFS/$NADPH_4$ complex titrated with product **1a** (**c**) and substrate **3** (**d**). All curves were obtained at constant [GFS] (subunit) and saturating [$NADP^+$] or [$NADPH_4$], with varied [**1a**] or [**3**]. The concentrations are shown in the panels. Buffer: 10 mM Tris, 25 mM NaCl, pH 8.0 (25 °C). Error bars show the standard deviation of triplicate measurements. Dashed lines are best fit to Eq. S2.

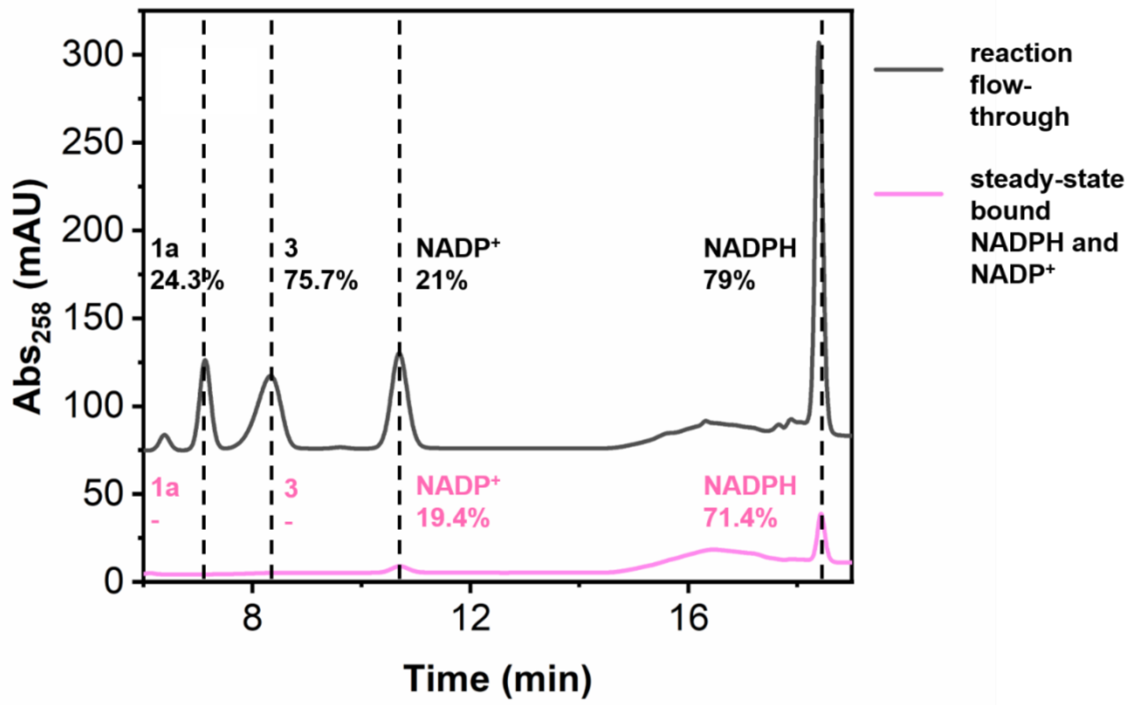

Figure S16. Quantification of enzyme-bound NADPH and $NADP^+$ at steady state of the reaction. HPLC chromatograms show the flow-through (grey) obtained after stopping the reaction at 3 min, and the supernatant from the denatured wild-type GFS (pink), representing the coenzymes released from the enzyme active site.

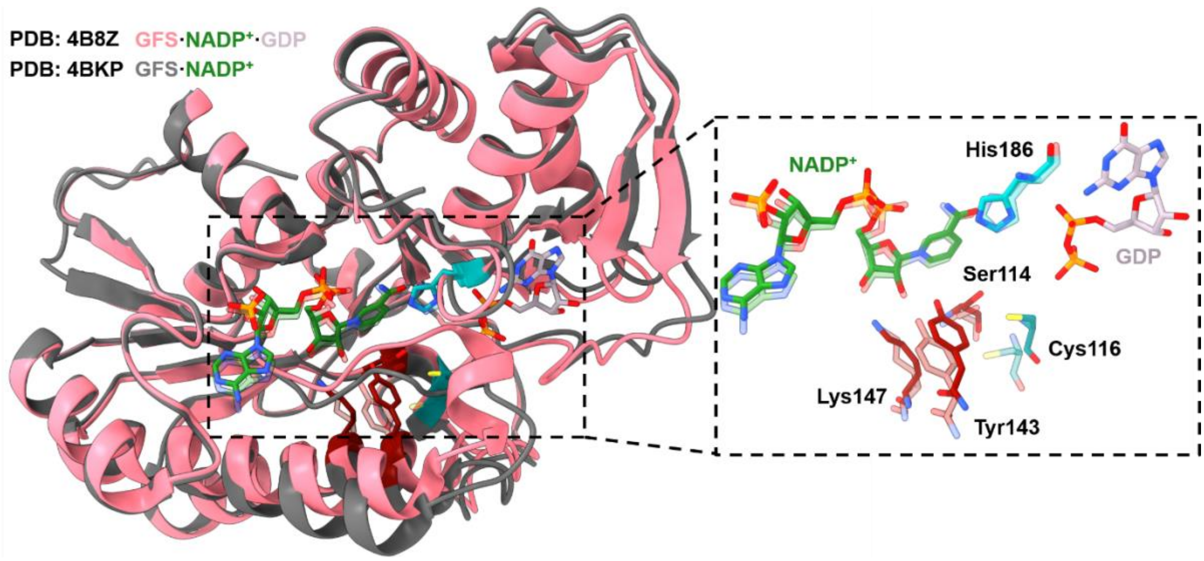

Figure S17. Superimposed structures (RMSD 1.424 Å) of GFS complexes with $NADP^+$ (4BKP, grey) and $NADP^+$/GDP (4B8Z, pink). A zoom into the active site is shown, with $NADP^+$ (dark green), GDP (light purple) and the catalytic residues indicated. Residues and ligand from the $NADP^+$ complex (4BKP) are shown with 60% transparency.

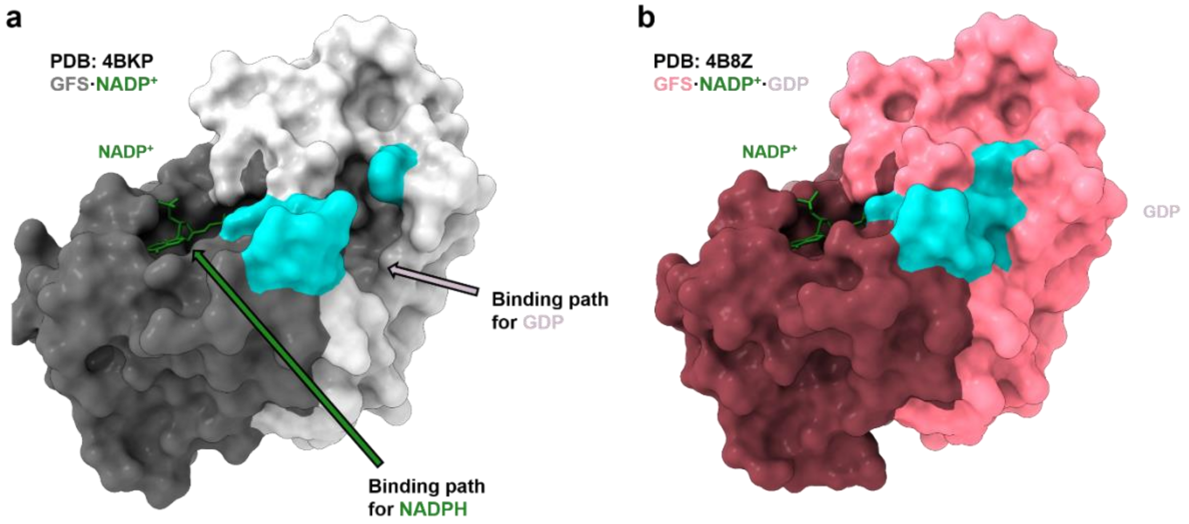

Figure S18. Surface representation of GFS complex structures with NADP$^+$ (4BKP, **a**) and NADP$^+$/GDP (4B8Z, **b**) showing distinct entry points in the structure for NADPH and substrate binding. The Rossman-fold like domain for NADPH binding is highlighted in dark grey and dark pink; the mostly α-helical substrate binding domain highlighted in light grey and bright pink. The flexible region involved in GDP binding (residues 74–82, Trp208) is highlighted in blue.

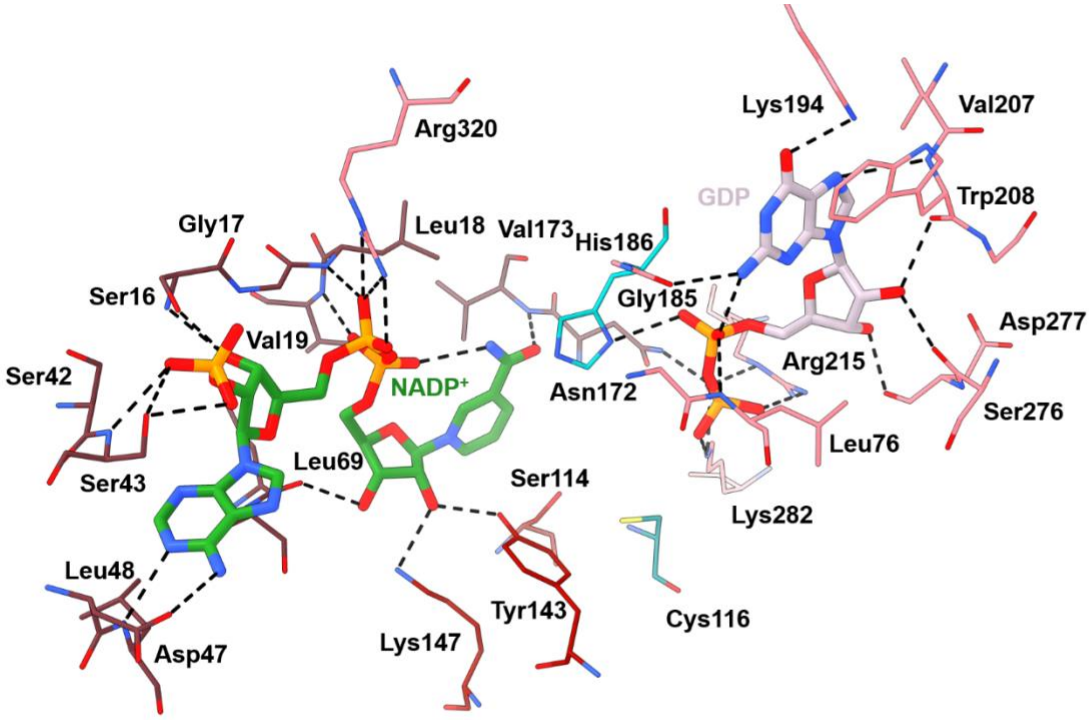

Figure S19. Binding pocket of the GFS complex structure with NADP$^+$/GDP. Black dashed lines represent hydrogen bonds between NADP$^+$ (green) and the coenzyme-binding domain (residues in dark pink), and between GDP (light purple) and the substrate-binding domain (residues in bright pink) of GFS (4B8Z).

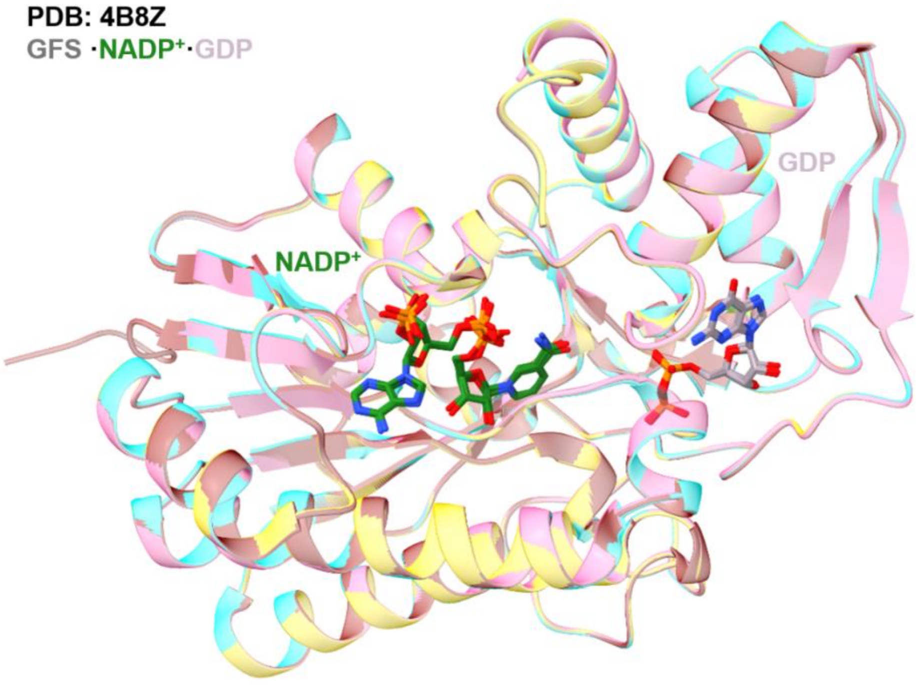

Figure S20. Superimposed monomers from the asymmetric unit of the GFS complex structure with NADP$^+$/GDP (4B8Z, RMSD< 0.124 Å).

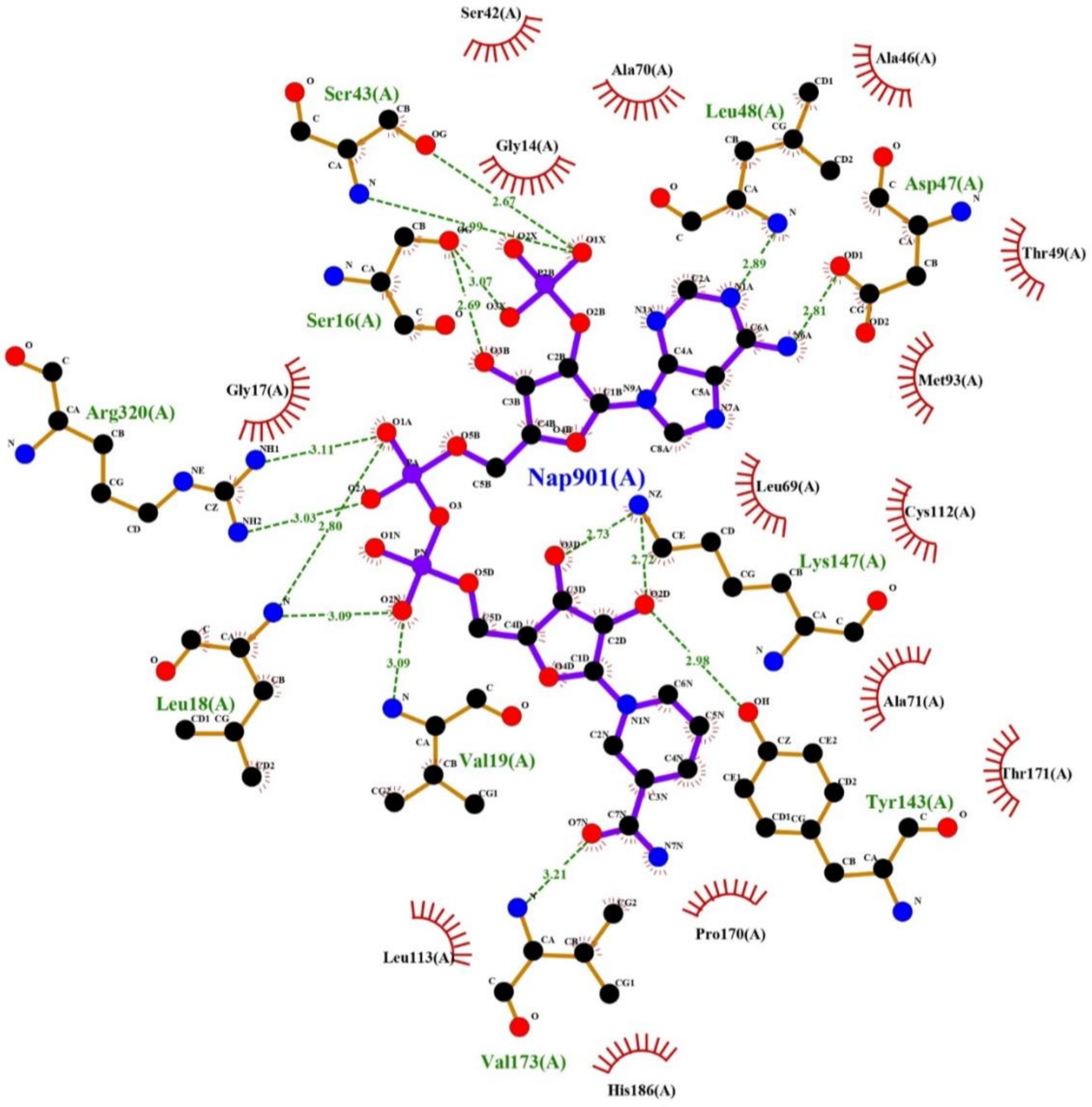

Figure S21. LigPlot diagram of NADP$^+$ interactions in the GFS complex with NADP$^+$ (4BKP, subunit A).

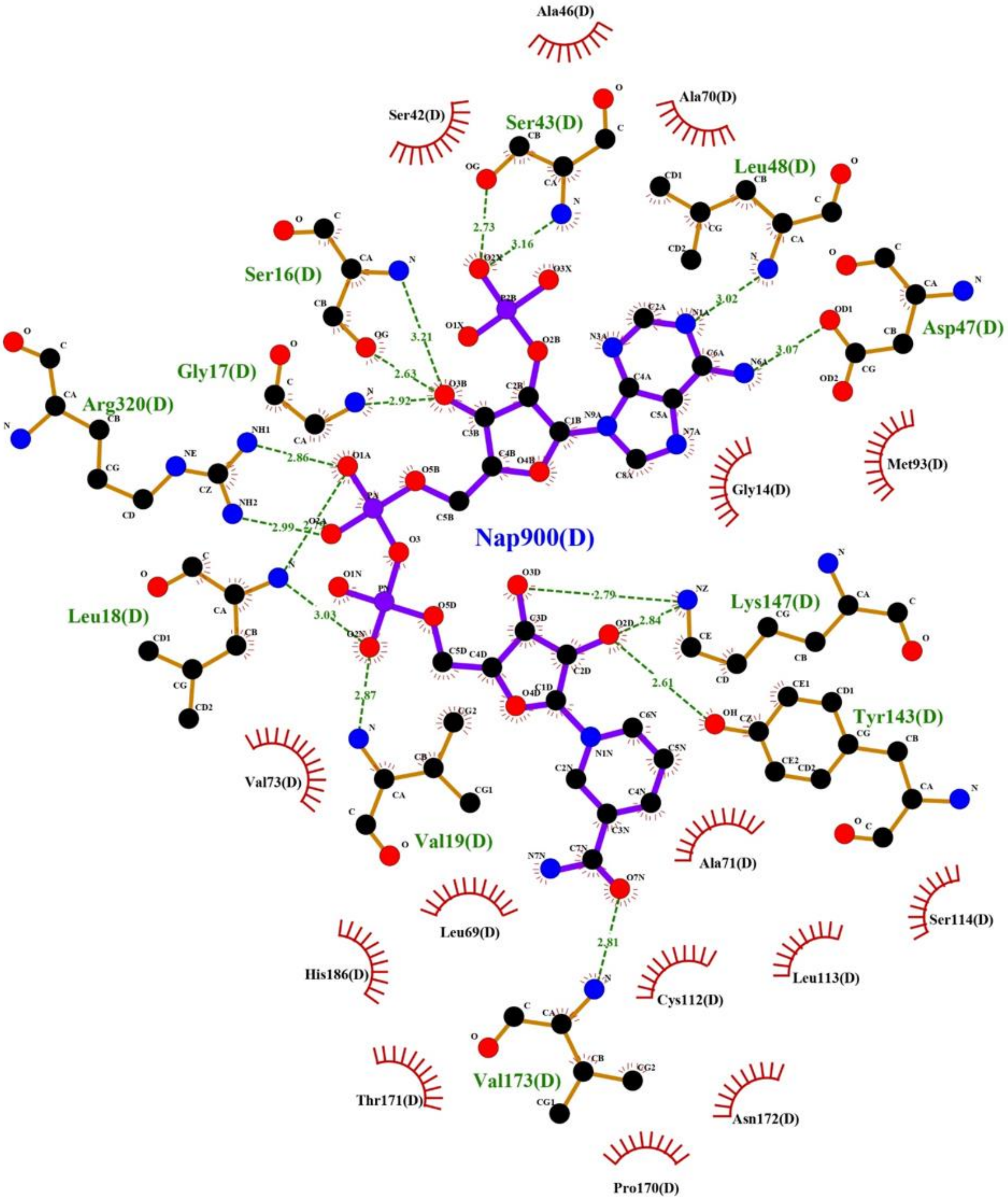

Figure S22. LigPlot diagram of $NADP^+$ interactions in the GFS complex with $NADP^+$/GDP (4B8Z, subunit D).

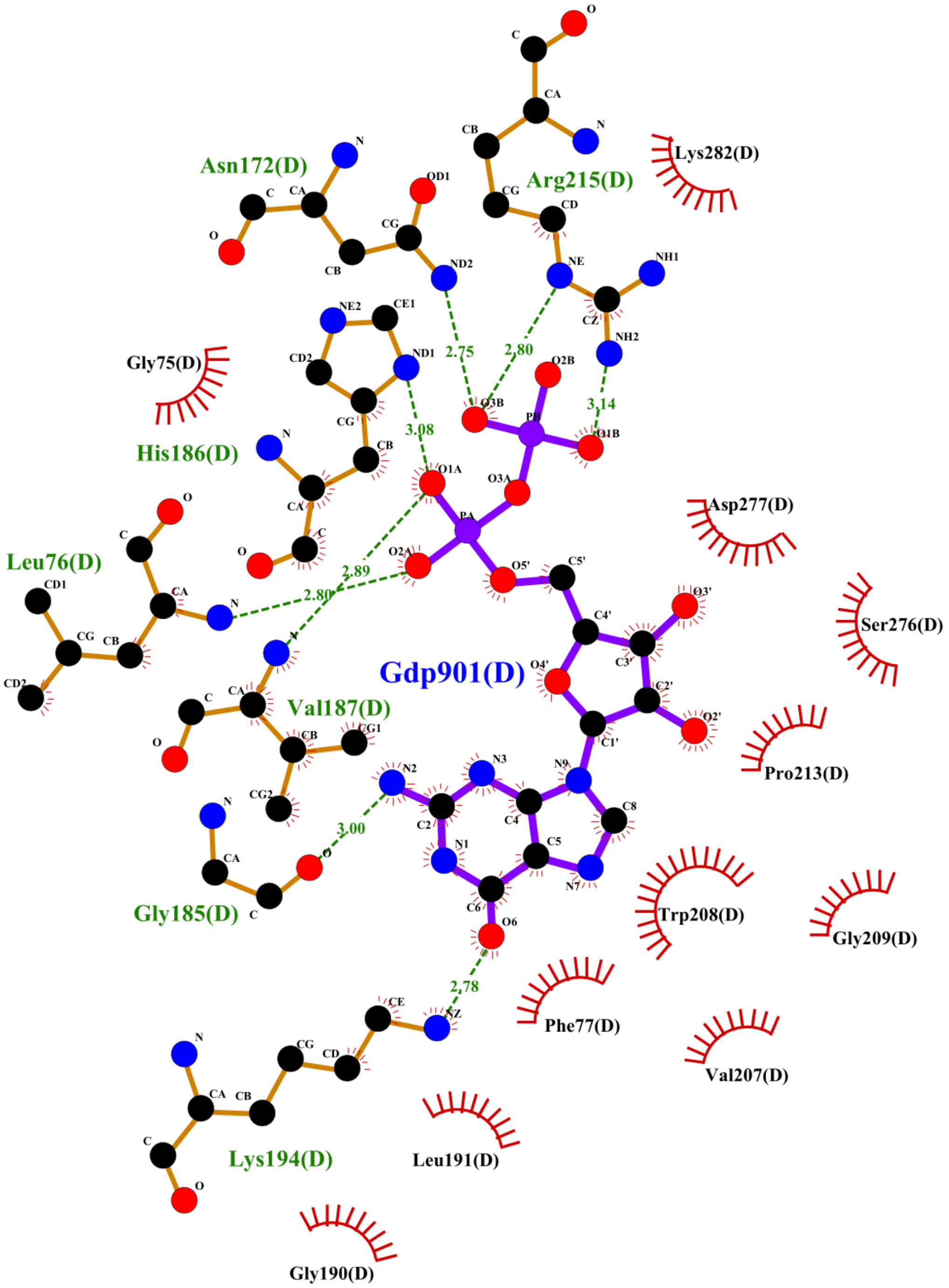

Figure S23. LigPlot diagram of GDP interactions in the GFS complex with NADP+/GDP (4B8Z, subunit D).

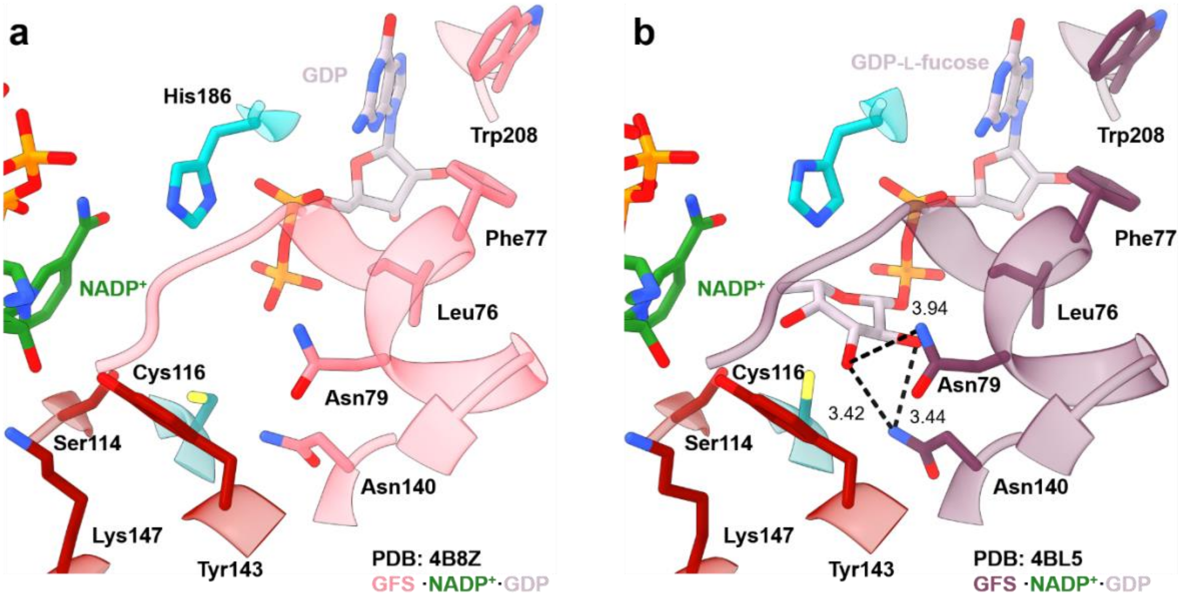

Figure S24. Close-up views of the active site and the substrate binding loop (residues 74–82) of GFS complex structures with NADP$^+$/GDP (4B8Z, **a**) and NADP$^+$/GDP-L-fucose (4BL5, **b**). Interactions with the sugar moiety (Å) are indicated by black dashed lines. Despite presence of sugar moiety in (**b**), residue positioning remains unchanged, with the GDP phosphate group maintaining the alignment of catalytic and binding residues.

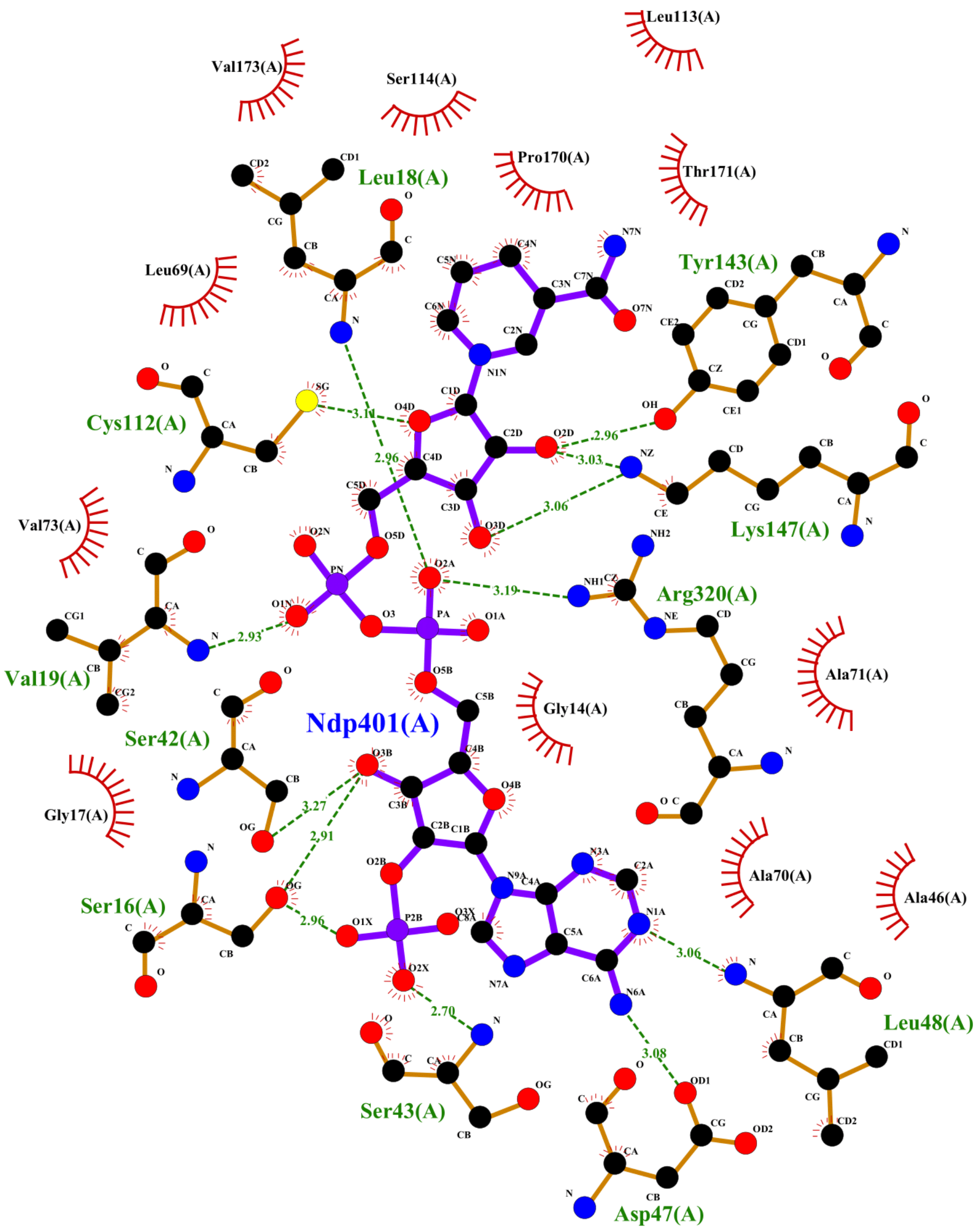

Figure S25. LigPlot diagram of NADPH interactions in the GFS complex with NADPH (4B8Z, subunit D).

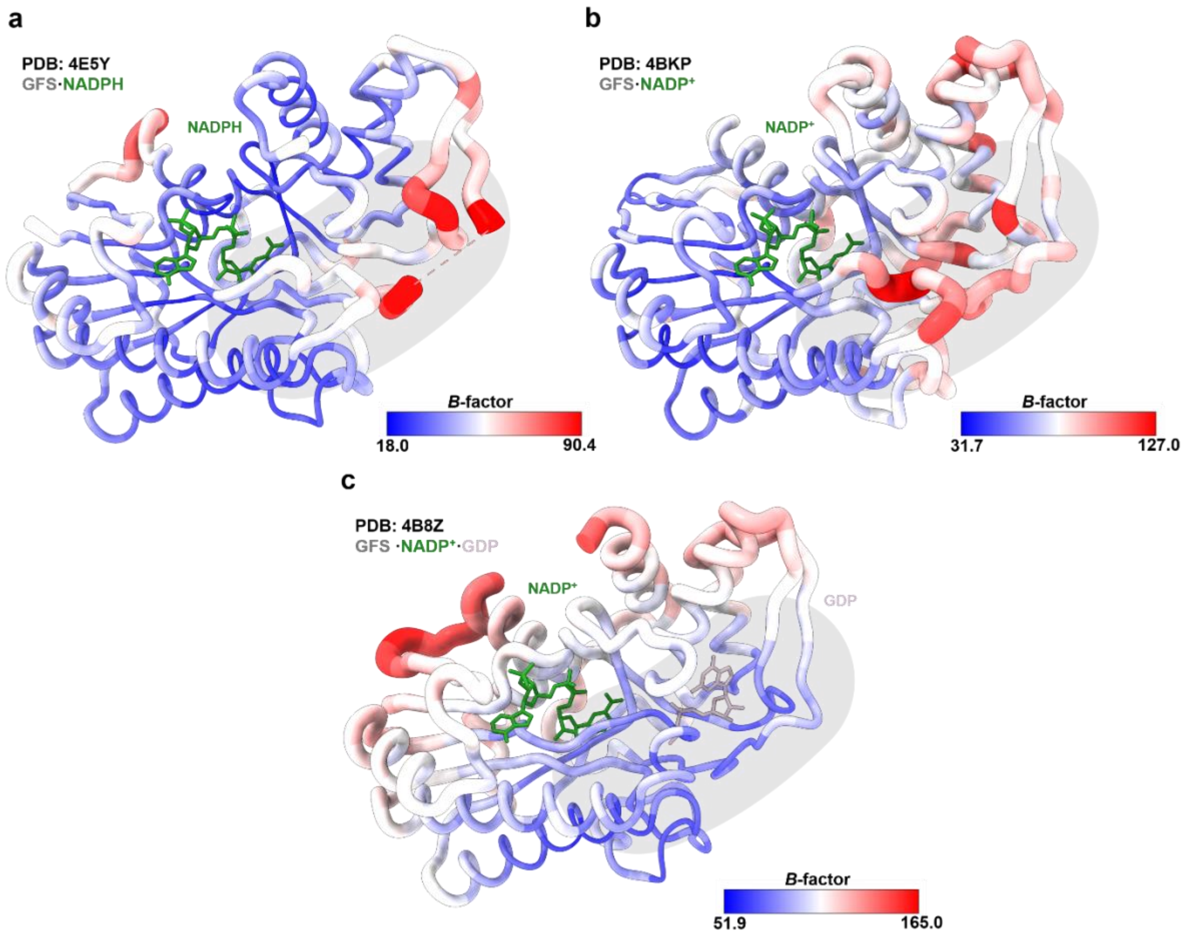

Figure S26. *B*-factor diagram of GFS complex structures with NADPH (4E5Y, **a**), NADP$^+$ (4BKP, **b**) and NADP$^+$/GDP (4B8Z, **c**). The flexible region, highlighted in gray, undergoes a conformational change upon GDP binding, resulting in increased rigidity.

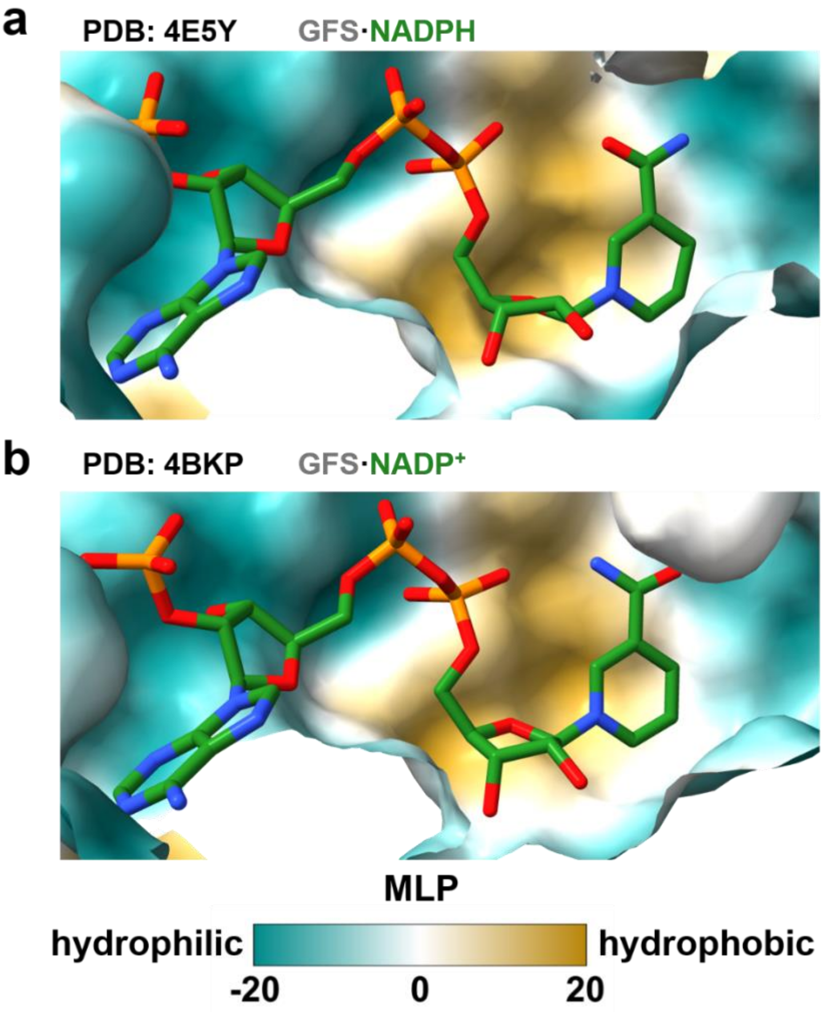

Figure S27. Surface representation of the NADPH/NADP⁺ binding site in GFS structures, highlighting the hydrophobicity of residues. Close-up views of the binding pocket GFS structures with NADPH (4E5Y, **a**) and NADP⁺ (4BKP, **b**). Surface representation colored based on molecular lipophilicity potential (MLP) calculated in ChimeraX.[18]

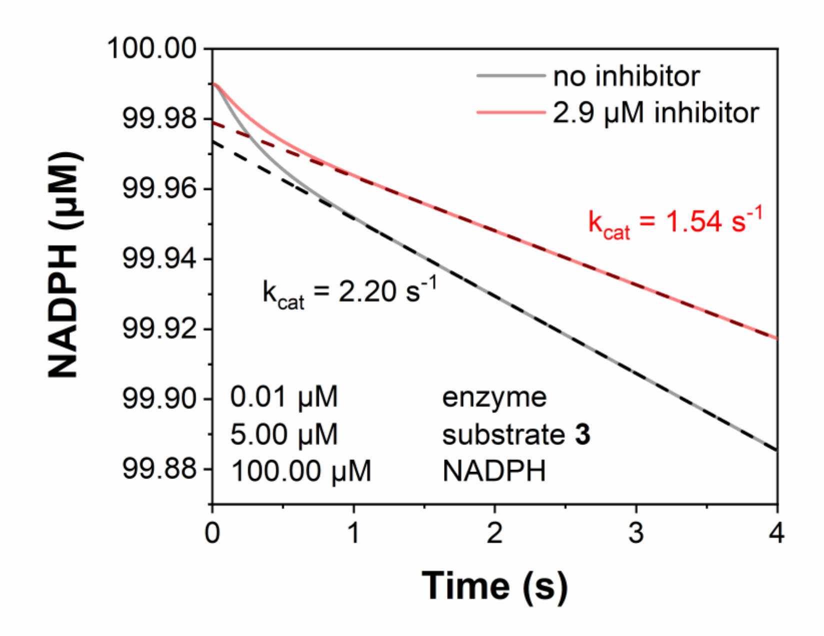

Figure S28. Simulated multiple-turnover progress curves in the absence and presence of the inhibitor GDP-6″-alkynyl-L-fucose (K_i = 2.7 μM [19]). [GFS] (subunit), [NADPH] and [substrate **3**] are shown in the graph. Simulations are based on mechanism b using the associated rate constants from Table S4. Inhibitor binding to the enzyme complex with NADPH was modeled with the assumption of rapid equilibrium, with k_5 describing dissociation from the abortive ternary complex.

SUPPORTING SCHEMES

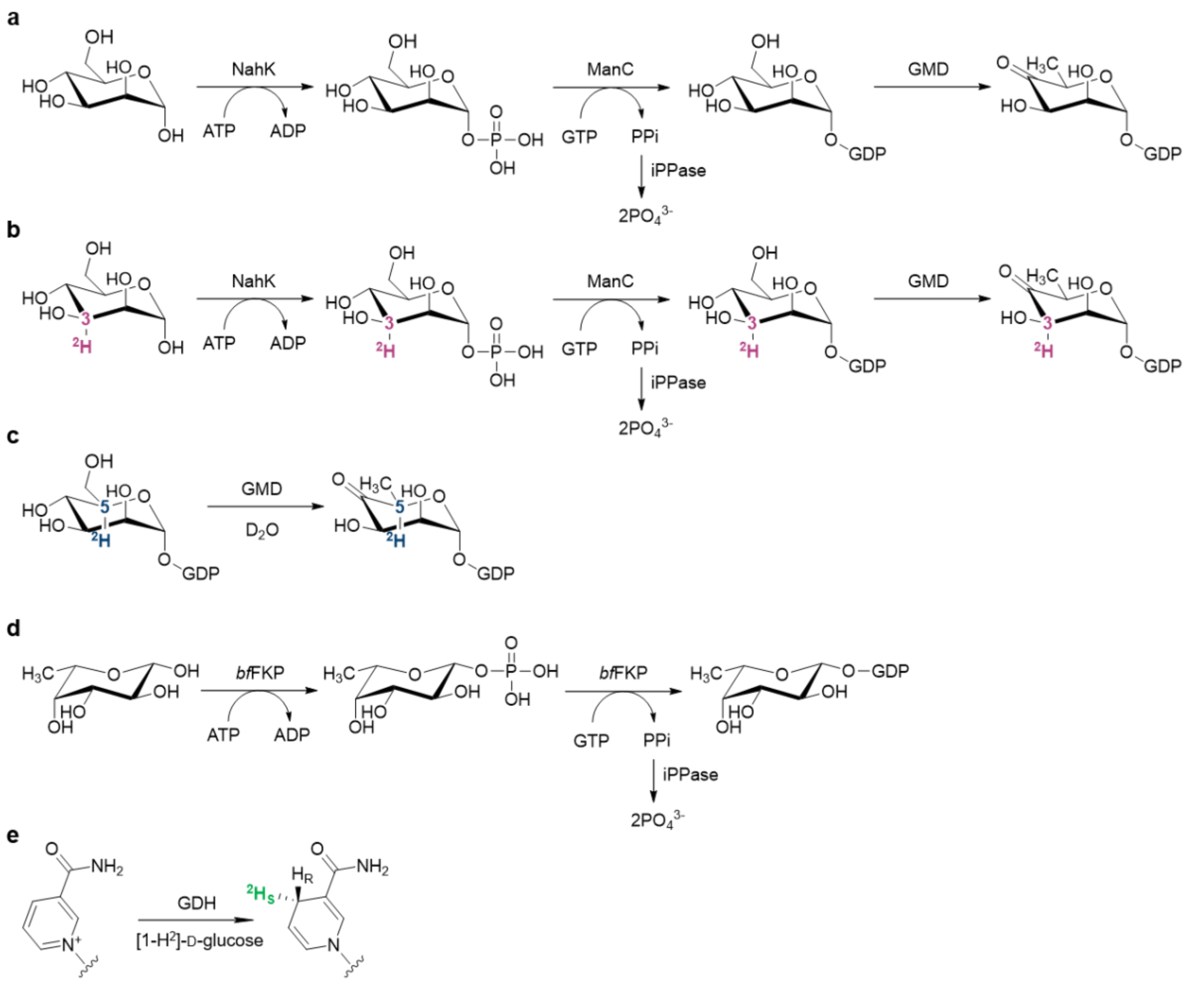

Scheme S1. Enzymatic synthesis of substrates. (**a**) Substrate **3**: *N*-acetylhexosamine 1- kinase (NahK) and ATP were used for anomeric phosphorylation of D-mannose; D-mannose-1-phosphate was incubated with GTP, D-mannose-1-phosphate guanylyltransferase (ManC) and inorganic pyrophosphatase (iPPase); GDP-D-mannose was incubated with human GDP-D-mannose-4,6-dehydratase (GMD); (**b**) [3″-^{2}H]-**3**: same reaction sequence as for **3**, starting from [3-^{2}H]-D-mannose; (**c**) [5″-^{2}H]-**3**: starting from GDP- D-mannose, incubation with hGMD was conducted in $^{2}H_2O$; (**d**) L-fucokinase/GDP-L-fucose pyrophosphorylase (*bf*FKP) and ATP were used for anomeric phosphorylation of L-fucose; L-fucose-1-phosphate was incubated with GTP, *bf*FKP and iPPase; (**e**) [4*S*-^{2}H]-NADPH: glucose dehydrogenase (GDH) and [1-^{2}H]-D-glucose were used for reduction of NADP^{+}.

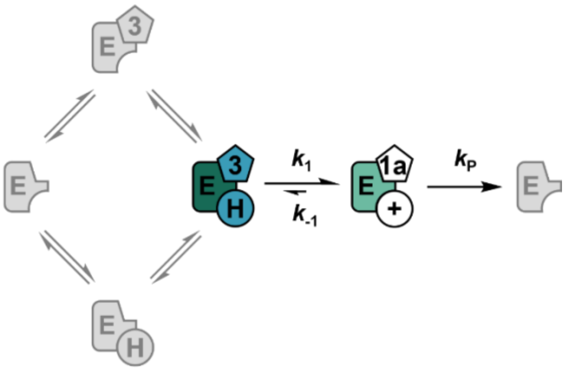

Scheme S2. Proposed kinetic mechanism of H186K variant involving random substrate binding and product release. Substrate (**3**) and product (**1a**) are identified by their compound number, **H** and + show NADPH and NADP$^+$, respectively. E is GFS. Enzyme forms shown in grey do not accumulate in significant amount at steady state.

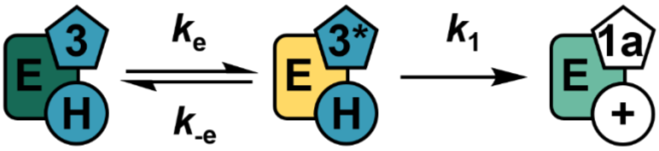

Scheme S3. Kinetic mechanism featuring an expanded (two-step) chemical step of GFS catalysis comprised of a reversible epimerization step and an irreversible reduction. Substrate (**3**) and product (**1a**) are identified by their compound number, the epimerized intermediate is identified as **3***, **H** and + show NADPH and NADP$^+$, respectively. E is GFS.

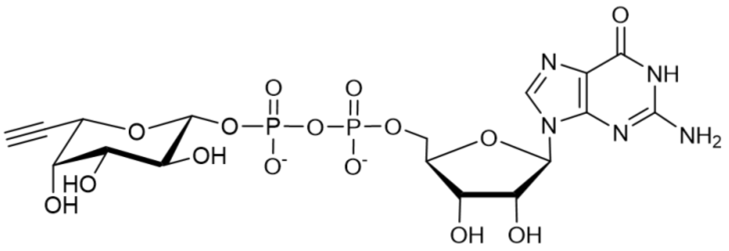

Scheme S4. Structure of the GDP-6″-alkynyl-L-fucose, a GDP-L-fucose analogue and GFS inhibitor.[19]

SUPPORTING TABLES

Table S1. Specific enzyme activity and enzyme-bound NADPH/$NADP^+$ of different GFS enzymes.

GFS	Activity, U/mg	NADPH, %	$NADP^+$, %
Wild-type	0.44 ± 0.02	43	3
C116A	Below detection limit	-[a]	-[a]
C116S	0.0010 ± 0.0003	24	6
H186A	Below detection limit	-[a]	-[a]
H186K	0.12 ± 0.01	46	2
Y143F	0.014 ± 0.001	90	1

[a] Not determined.

Table S2. Kinetic parameters from fits of transient multiple-turnover curves of reactions catalyzed by wild-type enzyme and H186K. Single- and double-exponential burst equations were used for fitting.

Single-exp. burst[a]	Wild-type	H186K	Double-exp. burst[b]	Wild-type
$[NADPH]_{burst}$ (μM)	7.47 ± 0.07	3.61 ± 0.01	$[NADPH]_{burst1}$ (μM)	1.73 ± 0.01
k_{obs} (s^{-1})	3.87 ± 0.03	3.93 ± 0.02	k_{obs1} (s^{-1})	15.4 ± 0.1
k_{ss} (s^{-1})	2.15 ± 0.01	0.38 ± 0.01	$[NADPH]_{burst2}$ (μM)	7.05 ± 0.01
Y_0 (μM)	292.1 ± 0.3	295.82 ± 0.04	k_{obs2} (s^{-1})	3.03 ± 0.01
			k_{ss} (s^{-1})	2.10 ± 0.01
			Y_0 (μM)	292.2 ± 0.2

[a] Fitting with Eq. S3
[b] Fitting with Eq. S5

Table S3. Equilibrium dissociation constants (K_d) determined from fluorescence titration studies.

Ligand	K_d, μM	Ligand	K_d, μM	
	apo GFS		enzyme/$NADPH_4$	enzyme/$NADP^+$
**3**	30 ± 10	**3**	0.5 ± 0.1	10 ± 2
$NADPH_4$	0.3 ± 0.1	**1a**	1.1 ± 0.2	13 ± 4
$NADP^+$	8 ± 2			

Fitting with Eq. S6

Table S4. Microscopic rate constants obtained from global simulation-fitting of mechanisms **a** and **b** to transient stopped-flow curves for the wild-type enzyme.

Rate constants	Mechanism a	Mechanism b	Calculation range	
			Lower	Upper
k_1 (s^{-1})	15.8 ± 0.1	15.4 ± 0.1	0.01	100
k_{-1} (s^{-1})	< 0.1	< 0.1	0.01	100
k_2 (s^{-1})	23 ± 3	26 ± 4	0.01	100
k_3 (s^{-1})	11 ± 1	12 ± 2	0.01	100
k'_2[**3**] (s^{-1})		>> k_1, k_2, k_4		
k'_3[NADPH] (s^{-1})		>> k_1, k_3, k_5		
k_4 (s^{-1})	31 ± 3	25 ± 3	0.01	100
k_5 (s^{-1})	1.03 ± 0.02	0.94 ± 0.01	0.01	100
$k'_{2,3}$ (s^{-1})	2.6 ± 0.1[a]	2.5 ± 0.1[b]		
k_{cat}^{SF} (s^{-1})	2.2 ± 0.1[a]	2.2 ± 0.1[b]	2.0	2.4

[a] k_{cat}^{SF} and $k'_{2,3}$ calculation according to mechanism a, based on Eq. S7-S9

$$k_{ss}^{SF} = \frac{k'_1 k'_{2,3}}{k'_1 + k'_{2,3}} \qquad k'_1 = \frac{k_1(k_2+k_3)}{k_{-1}+k_2+k_3}$$

$$k'_{2,3} = \frac{k_2+k_3}{1+\frac{k_2}{k_4}+\frac{k_3}{k_5}}$$

[b] k_{cat}^{SF} and $k'_{2,3}$ calculation according to mechanism b, based on Eq. S7, S8 and S10

$$k_{ss}^{SF} = \frac{k'_1 k'_{2,3}}{k'_1 + k'_{2,3}} \qquad k'_1 = \frac{k_1(k_2+k_3)}{k_{-1}+k_2+k_3}$$

$$k'_{2,3} = \frac{k_2+k_3}{1+\frac{k_2}{k'_2[3]}+\frac{k_2}{k_4}+\frac{k_3}{k'_3[\mathrm{NADPH}]}+\frac{k_3}{k_5}}$$

Table S5. Microscopic rate constants from global simulation-fitting of Scheme S2 to transient stopped-flow curves for H186K.

Rate constants	Scheme S2	Calculation limits	
		Lower	Upper
k_1 (s^{-1})	2.03 ± 0.05	0.01	100
k_{-1} (s^{-1})	1.2 ± 0.1	0.01	100
k_P (s^{-1})	0.75 ± 0.02	0.01	100
k_{cat}^{SF} (s^{-1})	0.39 ± 0.04[a]	0.1	0.6

[a] k_{cat}^{SF} calculation according to Scheme S2, based on Eq. S13.

$$k_{cat}^{SF} = \frac{k_1 k_P}{k_1 + k_{-1} + k_P}$$

Table S6. Data collection and refinement statistics (molecular replacement).

	PDB: 4B8Z	PDB: 4BKP
**Data collection**		
Space group	P 4 2	I 2 2 2
Cell dimensions		
a, b, c (Å)	111.47, 111.47 46.92	106.60, 163.09, 197.58
α, β, γ (°)	90, 90, 90	90, 90, 90
Resolution (Å)	40.32 – 2.75	49.44 – 2.70
R_{merge}	0.11	0.10
$I/\sigma I$	1.65	2.16
Completeness (%)	93.3	100.0
Redundancy	3.8 (3.8)	6.8 (6.9)
**Refinement**		
Resolution(Å)	40.32 – 2.75	49.44 – 2.70
No. reflections	34584	47605
R_{work} / R_{free}	0.201 / 0.252	0.196 / 0.231
No. atoms		
Protein	9492	10056
Ligand/ion	304	209
Water	18	50
B-factors		
Protein	75.4	37.5
Ligand/ion	70.8	48.3
Water	52.8	36.3
R.m.s. deviations		
Bond lengths (Å)	0.008	0.007
Bond angles (°)	0.91	1.198

Table S7. Nucleotide and protein sequence of GFS enzyme with the corresponding expression vector used in this study.

**GFS** (with His-Tag and TEV restriction site)	
Vector	pNIC28
Nucleotide sequence	ATGCACCATCATCATCATCATTCTTCTGGTGTAGATCTGGGTACCGAGAA CCTGTACTTCCAATCCATGCGGATTCTAGTGACAGGGGGCTCTGGGCTGG TAGGCAAAGCCATCCAGAAGGTGGTAGCAGATGGAGCTGGACTTCCTGG AGAGGACTGGGTGTTTGTCTCCTCTAAAGACGCCGATCTCACGGATACAG CACAGACCCGCGCCCTGTTTGAGAAGGTCCAACCCACACACGTCATCCAT CTTGCTGCAATGGTGGGGGGCCTGTTCCGGAATATCAAATACAATTTGGA CTTCTGGAGGAAAAACGTGCACATGAACGACAACGTCCTGCACTCGGCC TTTGAGGTGGGCGCCCGCAAGGTGGTGTCCTGCCTGTCCACCTGTATCTT CCCTGACAAGACGACCTACCCGATAGATGAGACCATGATCCACAATGGG CCTCCCCACAACAGCAATTTTGGGTACTCGTATGCCAAGAGGATGATCGA CGTGCAGAACAGGGCCTACTTCCAGCAGTACGGCTGCACCTTCACCGCTG TCATCCCCACCAACGTCTTCGGGCCCCACGACAACTTCAACATCGAGGAT GGCCACGTGCTGCCTGGCCTCATCCACAAGGTGCACCTGGCCAAGAGCA GCGGCTCGGCCCTGACGGTGTGGGGTACAGGGAATCCGCGGAGGCAGTT CATATACTCGCTGGACCTGGCCCAGCTCTTTATCTGGGTCCTGCGGGAGT ACAATGAAGTGGAGCCCATCATCCTCTCCGTGGGCGAGGAAGATGAGGT CTCCATCAAGGAGGCAGCCGAGGCGGTGGTGGAGGCCATGGACTTCCAT GGGGAAGTCACCTTTGATACAACCAAGTCGGATGGGCAGTTTAAGAAGA CAGCCAGTAACAGCAAGCTGAGGACCTACCTGCCCGACTTCCGGTTCAC ACCCTTCAAGCAGGCGGTGAAGGAGACCTGTGCTTGGTTCACTGACAAC TACGAGCAGGCCCGGAAGTGA
Protein sequence	MHHHHHHSSGVDLGTENLYFQSMRILVTGGSGLVGKAIQKVVADGAGLPG EDWVFVSSKDADLTDTAQTRALFEKVQPTHVIHLAAMVGGLFRNIKYNLDF WRKNVHMNDNVLHSAFEVGARKVVSCLSTCIFPDKTTYPIDETMIHNGPPH NSNFGYSYAKRMIDVQNRAYFQQYGCTFTAVIPTNVFGPHDNFNIEDGHVL PGLIHKVHLAKSSGSALTVWGTGNPRRQFIYSLDLAQLFIWVLREYNEVEPII LSVGEEDEVSIKEAAEAVVEAMDFHGEVTFDTTKSDGQFKKTASNSKLRTY LPDFRFTPFKQAVKETCAWFTDNYEQARK

Figure S8. Oligonucleotide primers used in this study for site-directed mutation of GFS. The mutated sites are underlined.

Primer name	DNA primer sequences (5‘ - 3‘)
C116S_fwd	CTGTCCACCTCTATCTTCCCTGACAAGACGAC
C116S_rev	CAGGGAAGATAGAGGTGGACAGGCAGGACA
C116A_fwd	CCTGCCTGTCCACCGCCATCTTCCCTGACAAG
C116A_rev	CTTGTCAGGGAAGATGGCGGTGGACAGGCAGG
H186K_fwd	GACAACTTCAACATCGAGGATGGCAAAGTGCTGCCTGGCCTCATCCACAAG
H186K_rev	CTTGTGGATGAGGCCAGGCAGCACTTTGCCATCCTCGATGTTGAAGTTGTC
H186A_fwd	CATCGAGGATGGCGCCGTGCTGC
H186A_rev	GCCAGGCAGCACGGCGCCATC
Y143F_fwd	CAATTTTGGGTTCTCGTATGCCAAGAGGATGATC
Y143F_rev	GGCATACGAGAACCCAAAATTGCTGTTGTGG

SUPPORTING REFERENCES

(1) Nishimoto, M.; Kitaoka, M. Identification of *N*-acetylhexosamine 1-kinase in the complete lacto-*N*-biose I/galacto-*N*-biose metabolic pathway in *Bifidobacterium longum*. *Appl. Environ. Microbiol.* **2007**, *73*, 6444–6449.

(2) Pfeiffer, M.; Johansson, C.; Krojer, T.; Kavanagh, K. L.; Oppermann, U.; Nidetzky, B. A Parsimonious mechanism of sugar dehydration by human GDP-mannose-4,6-dehydratase. *ACS Catal.* **2019**, *9*, 2962–2968.

(3) Pfeiffer, M.; Bulfon, D.; Weber, H.; Nidetzky, B. A kinase-independent one-pot multienzyme cascade for an expedient synthesis of guanosine 5′-diphospho-D-mannose. *Adv. Synth. Catal.* **2016**, *358*, 3809–3816.

(4) Ohashi, H.; Wahl, C.; Ohashi, T.; Elling, L.; Fujiyama, K. Effective synthesis of guanosine 5′-diphospho-β-L-galactose using bacterial L-fucokinase/guanosine 5′-diphosphate-L-fucose pyrophosphorylase. *Adv. Synth. Catal.* **2017**, *359* (23), 4227–4234.

(5) Edelheit, O.; Hanukoglu, A.; Hanukoglu, I. Simple and efficient site-directed mutagenesis using two dingle-primer reactions in parallel to generate mutants for protein structure-function studies. *BMC Biotechnol.* **2009**, *9*, 1–8.

(6) *COPASI v 4.44*, available free of charge from http://copasi.org.

(7) Kabsch, W. XDS. *Acta Crystallogr. D Biol. Crystallogr.* **2010**, *66*, 125–132.

(8) Evans, P. R.; Murshudov, G. N. How good are my data and what is the resolution? *Acta Crystallogr. D Biol. Crystallogr.* **2013**, *69*, 1204–1214.

(9) Winter, G. Xia2: an expert system for macromolecular crystallography data reduction. *J. Appl. Crystallogr.* **2010**, *43*, 186–190.

(10) McCoy, A. J.; Grosse-Kunstleve, R. W.; Adams, P. D.; Winn, M. D.; Storoni, L. C.; Read, R. J. Phaser crystallographic software. *J. Appl. Crystallogr.* **2007**, *40*, 658–674.

(11) Emsley, P.; Lohkamp, B.; Scott, W. G.; Cowtan, K. Features and development of COOT. *Acta Crystallogr. D Biol. Crystallogr.* **2010**, *66*, 486–501.

(12) Adams, P. D.; Afonine, P. V.; Bunkóczi, G.; Chen, V. B.; Davis, I. W.; Echols, N.; Headd, J. J.; Hung, L. W.; Kapral, G. J.; Grosse-Kunstleve, R. W.; McCoy, A. J.; Moriarty, N. W.; Oeffner, R.; Read, R. J.; Richardson, D. C.; Richardson, J. S.; Terwilliger, T. C.; Zwart, P. H. PHENIX: A comprehensive python-based system for macromolecular structure solution. *Acta Crystallogr. D Biol. Crystallogr.* **2010**, *66*, 213–221.

(13) Chen, V. B.; Arendall, W. B.; Headd, J. J.; Keedy, D. A.; Immormino, R. M.; Kapral, G. J.; Murray, L. W.; Richardson, J. S.; Richardson, D. C. MolProbity: All-atom structure validation for macromolecular crystallography. *Acta Crystallogr. D Biol. Crystallogr.* **2010**, *66*, 12–21.

(14) Meng, E. C.; Goddard, T. D.; Pettersen, E. F.; Couch, G. S.; Pearson, Z. J.; Morris, J. H.; Ferrin, T. E. UCSF ChimeraX: Tools for structure building and analysis. *Protein Sci.* **2023**, *32*, No. e4792, From NLM.

(15) Laskowski, R. A.; Swindells, M. B. LigPlot+: Multiple ligand-protein interaction diagrams for drug discovery. *J. Chem. Inf. Model.* **2011**, *51*, 2778–2786.

(16) Thompson, J. D.; Higgins, D. G.; Gibson, T. J. CLUSTAL W: Improving the sensitivity of progressive multiple sequence alignment through sequence weighting, position-specific gap penalties and weight matrix choice. *Nucleic Acids Res.* **1994**, *22*, 4673–4680.

(17) Robert, X.; Gouet, P. Deciphering key features in protein structures with the new ENDscript server. *Nucleic Acids Res.* **2014**, *42*, W320–W324.

(18) Ghose, A. K.; Viswanadhan, V. N.; Wendoloski, J. J. Prediction of hydrophobic (lipophilic) properties of Small organic molecules using fragmental methods: an analysis of ALOGP and CLOGP methods. *J. Phys. Chem. A* **1998**, *102*, 3762–3772.

(19) Kizuka, Y.; Nakano, M.; Yamaguchi, Y.; Hsu, T.-L.; Wong, C.-H.; Taniguchi, N. An alkynyl-fucose halts hepatoma cell migration and invasion by inhibiting GDP-fucose-synthesizing enzyme FX, TSTA3. *Cell. Chem. Biol.* **2017**, *24*, 1467-1478.
